# Development, Cultural Adaptation, and Content Validation of Urdu Pain Neuroscience Education Materials for Low Back Pain in Pakistan

**DOI:** 10.3390/medsci14010054

**Published:** 2026-01-22

**Authors:** Muhammad Naseeb Ullah Khan, Aastha Malhotra, Melainie Cameron

**Affiliations:** 1 School of Health and Medical Sciences, University of Southern Queensland, Toowoomba 4350, Australia; lainie.cameron@unisq.edu.au; 2 School of Psychology and Wellbeing, University of Southern Queensland, Toowoomba 4350, Australia; aastha.malhotra@unisq.edu.au; 3 Faculty of Health, Southern Cross University, Lismore 2480, Australia

**Keywords:** low back pain, pain neuroscience education, cultural adaptation, Urdu, educational materials, content validity

## Abstract

**Background**: Pain neuroscience education (PNE) can support understanding of low back pain and facilitate engagement with active care. Most PNE materials have been developed in English, and there is little culturally adapted content for Urdu-speaking populations. Locally relevant educational resources may help improve clarity, acceptability, and communication in clinical settings. **Objective**: To develop, culturally adapt, and content-validate Urdu PNE materials for individuals with LBP and for use by healthcare professionals in Pakistan. **Methods**: A four-stage adaptation process was used. Phase 1 involved drafting a ten-module English PNE booklet and clinician guide based on contemporary pain-science literature. Phase 2 included forward–backward translation into Urdu and cultural adaptation by translators and a bilingual pain researcher. In Phase 3, three focus-group sessions with clinicians and a person with LBP informed iterative revisions. In Phase 4, a multidisciplinary panel (clinicians and individuals with LBP, *n* = 32) assessed seven domains of the final Urdu materials for clarity, relevance, and cultural appropriateness using Lawshe’s content validity ratio (CVR). **Results**: Focus-group feedback led to simplification of Urdu phrasing, refinement of metaphors, and adjustments to illustrations. All seven domains exceeded the minimum CVR threshold (0.30) for *n* = 32, with a mean overall CVR of 0.69 ± 0.12. Cultural appropriateness (CVR = 0.88) and content accuracy (CVR = 0.86) showed the highest agreement. **Conclusions**: The adapted Urdu PNE materials were judged to be clear, relevant, and culturally appropriate by clinicians and individuals with LBP. These materials may be useful for supporting pain-related education in clinical and community settings. These findings establish preliminary content validity; further studies are needed to evaluate feasibility, implementation, and clinical outcomes.

## 1. Introduction

Low back pain (LBP) is one of the leading causes of years lived with disability globally and affects people across regions and socioeconomic groups [1]. Clinical guidelines recommend active care, self-management strategies, and clear information to support recovery, particularly in cases of persistent or recurrent LBP [2]. Pain neuroscience education (PNE) has emerged as an educational approach used to help patients understand the biology of pain, including the roles of the nervous system, sensitisation, and the distinction between tissue injury and pain experience [3]. Evidence from systematic reviews and clinical trials suggests that PNE, when combined with rehabilitation, can reduce pain-related fear, catastrophising, disability, and healthcare utilisation [3,4,5].

However, most existing PNE resources have been developed in English and are shaped by Western communication styles, metaphors, and health beliefs [6]. Given that pain beliefs differ across cultural and linguistic groups, these differences can influence how people interpret and respond to pain [7,8]. Studies exploring pain beliefs in non-Western populations indicate that individuals may draw on culturally embedded explanations involving work patterns, family expectations, or spirituality [7]. Such variations highlight the importance of presenting pain-related information in ways that reflect local language, imagery, and values. Research on educational material development emphasises that interventions relying on stories, analogies, and visual examples are most effective when these elements are familiar and culturally appropriate [9].

Several researchers have developed culturally adapted PNE materials in non-English contexts. For example, Orhan et al. adapted PNE content for first-generation Turkish patients, modifying examples, illustrations, and metaphors to align with cultural norms and everyday experiences [10]. Similarly, Mukhtar et al. developed Hausa language PNE materials for chronic spinal pain, using culturally relevant drawings, simplified language, and narratives suited to local literacy patterns [11]. These culturally adapted tools were primarily evaluated for clarity, cultural fit, and comprehensibility, but most were not formally tested for clinical effectiveness, highlighting the need for ongoing assessment of both content and real-world utility. These studies demonstrate that PNE materials can be successfully adapted through structured processes involving experts, end-users, and iterative refinement [12].

In Pakistan, Urdu is the national language and is widely used across clinical and community settings. As in many low- and middle-income countries, there is limited availability of culturally adapted educational materials related to pain and musculoskeletal health [7]. Although research in South Asian contexts has described common use of structural or mechanical explanations for LBP and noted the influence of cultural beliefs on health-seeking behaviours, published educational tools aligned with contemporary pain science remain scarce [7,12]. Educational materials developed in English may not be easily understood by users with lower literacy levels, and metaphors commonly used in Western PNE resources may not reflect day-to-day experiences in Pakistani communities [10,11,12]. The use of locally meaningful metaphors, familiar imagery, and the use of simple, commonly understood Urdu terminology, avoiding technical or formal linguistic constructions, may support clearer communication of PNE concepts.

Best-practice recommendations for cross-cultural adaptation emphasise a multistep process, including forward–backward translation, expert review, and involvement of individuals from the target population [13,14]. These steps help ensure that translated materials maintain conceptual accuracy while also being linguistically clear and culturally relevant.

Quantitative validation using approaches such as the CVR may be used to evaluate the clarity, relevance, and appropriateness of adapted educational content [15,16]. Such procedures have been used in previous culturally adapted PNE studies and are well-suited for developing materials intended for diverse clinical and community settings.

To date, no Urdu-language PNE resource has been formally developed or validated for people with LBP in Pakistan. Creating such materials may help provide clinicians and patients with a consistent, culturally aligned source of information to support discussion about pain, movement, and recovery. Using commonly understood metaphors and simple illustrations may be particularly relevant for settings where consultation times are short and literacy levels vary. Therefore, the aim of this study was to develop, culturally adapt, and content-validate Urdu PNE materials for individuals with LBP and for use by healthcare professionals in Pakistan.

## 2. Materials and Methods

**Figure 1:**
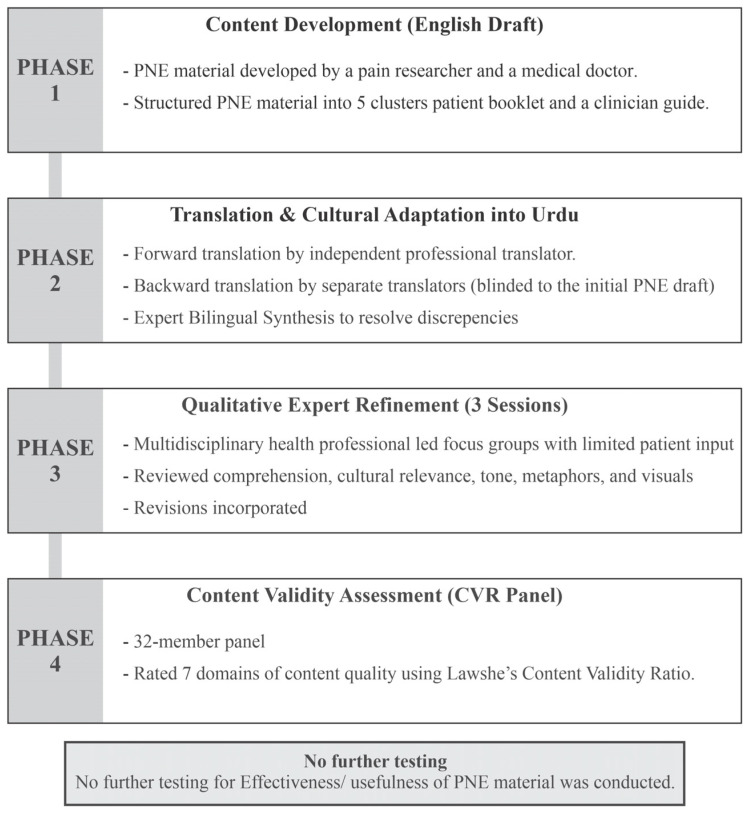
Development, cross-cultural translation, and adaptation process.

Study Design: In this study, we used a sequential, multistage design to develop, culturally adapt, and validate PNE material for people with LBP and healthcare professionals in Pakistan. The process followed established cross-cultural adaptation, measurement, and educational material development frameworks [17,18,19] and comprised four stages: (1) content development, (2) translation and cultural adaptation, (3) expert refinement through focus groups, and (4) quantitative content validation. A summary of the multistep development and adaptation process is provided in Figure 1.

Development of English PNE Material: An initial ten-module PNE was organised in 5 cluster PNE booklets and a clinician training guide in English. The content was derived from contemporary pain science literature, the Explain Pain framework [3], and biopsychosocial principles for chronic pain management. Each module integrated a single key educational concept (e.g., pain protection, movement, fear avoidance, resilience) with relatable metaphors and culturally flexible visuals. Parallel clinician pages included screening tools, including the back pain attitudes questionnaire (Back PAQ) [20], the Oswestry disability index (ODI) [21], the fear avoidance beliefs questionnaire (FABQ) [22], the pain catastrophizing scale (PCS) [23], and practical strategies for patient communication.

Translation and Cultural Adaptation: The materials were translated into Urdu following standard forward–backward translation protocols [13]. Two professional translators independently produced forward translations, which were synthesised by a team consisting of a bilingual pain researcher and a physiotherapist. A backward translation into English was completed by an independent translator blinded to the original text. Discrepancies were resolved by consensus, prioritising conceptual equivalence over literal translation to preserve meaning.

Expert Refinement (Focus Groups): Three face-to-face focus group sessions were conducted (60–90 min each) with a multidisciplinary sample: two physiotherapists, one medical specialist, one clinical psychologist, one pain researcher, and one person with chronic LBP. Participants were recruited through professional networks, local clinics, and direct invitations to clinicians who commonly treat patients with musculoskeletal pain. The LBP participant was recruited via a local physiotherapy clinic. Participants reviewed printed Urdu proofs and visual drafts, discussing accuracy, tone, readability, and cultural relevance. Sessions were transcribed verbatim and analysed using Framework Analysis [24,25]. Feedback-informed iterative revisions. Despite inviting several contributors, only one individual with LBP was available to contribute to these focus groups; accordingly, this phase is considered expert-led refinement with limited patient co-design. Although this is a limited patient representation, their lived experience contributed to identifying unclear or culturally incongruent content; the small number of patient contributors restricts confidence that language and metaphors reflect the breadth of patient perspectives across literacy levels and sociocultural subgroups in Pakistan. The Framework Analysis process involved familiarisation with transcripts, coding of recurrent concepts, development of a thematic framework, and charting of data into matrices to compare feedback across participants. Themes related to clarity, tone, cultural fit, and metaphor acceptability informed iterative revisions. The final English and Urdu versions of the PNE materials are provided in Supplementary Files S1 and S2.

Quantitative content validation (CVR analysis): The final Urdu materials were evaluated by a 32-member content validation panel consisting of eight physiotherapists, six medical specialists, eight clinical exercise professionals, and ten people with LBP. The ten modules were organised into five clusters based on conceptual similarity (protection, mind–body interactions, movement, lifestyle factors, and recovery), and evaluators rated the cluster level rather than the module level. Cluster-level rating was selected to reduce rater burden and improve completion feasibility, given the number of modules and domains, while preserving judgement at the conceptual unit level. Evaluators rated based on seven domains, including content accuracy, clarity, cultural appropriateness, illustration quality, flow, tone, and feasibility using a 3-point scale (1 = Not essential, 2 = Useful but not essential, 3 = Essential). The content validity ratio (CVR) was computed for each domain using Lawshe’s formula [15]:

CVR = (ne − N/2)/(N/2)

where ne = number rating the item as “essential” and *n* = total panel size. For *n* = 32, the minimum acceptable CVR was 0.30 (*p* < 0.05) [11,16]. Although multi-round Delphi processes are sometimes recommended, a single round of content validation was undertaken in this research, as CVR values exceeded the minimum threshold for *n* = 32 across all domains, and iterative rounds were not considered necessary at this stage. The characteristics of the multidisciplinary content validation panel are summarised in Table 1.

Ethical Considerations: The project was approved by the University of Southern Queensland Human Research Ethics Committee (HREC No. ETH2023-0054). All participants provided written informed consent prior to participation.

## 3. Results

The ten-module PNE materials were initially drafted into 5 clusters in English and iteratively refined across three face-to-face focus group sessions (60–90 min each). Participants included two physiotherapists, one medical specialist, one clinical psychologist, one pain researcher, and one person with chronic low back pain. Feedback across sessions consistently centred on four themes: (1) Simplifying language and reducing biomedical terminology; (2) Ensuring metaphors matched everyday Pakistani experiences; (3) Adjusting illustrations to reflect modest attire and gender inclusivity; and (4) Improving flow by placing foundational concepts earlier in the sequence.

Key modifications included simplification of Urdu phrasing, shortening of paragraphs, and substitution of complex biomedical terms (e.g., “sensitisation”) with everyday, non-technical Urdu expressions that are widely understood (e.g., محتاط ہونا نظام کا زیادہ—“system being over-protective”). Illustrations were redrawn with gender-balanced figures in modest attire, and metaphors familiar to Pakistani audiences were adopted, such as “گھر بارش میں” (House in Rain) for over-protective pain responses and “ایمان اور عمل” (Faith and Action) for self-care. By the third session, verbal feedback captured in the focus group transcripts reflected that participants perceived the overall tone as respectful and non-judgmental and that the core messages were generally clear to this stakeholder group. Minor refinements focused on improving the logical order of modules and ensuring that references to faith were inclusive and did not assume a single denomination or level of religious practice. Faith-related framing was included as a culturally grounded communication strategy and was not tested in this study for incremental effects on acceptability or behaviour change. Participants also suggested that future versions could incorporate audio or video explainers (e.g., via QR codes) to support users with low literacy; this feedback informed the structure of the written modules, but multimedia components were not developed or evaluated in this study.

Content validity outcomes (CVR analysis): All seven domains exceeded the minimum CVR threshold (0.30) for *n* = 32. Across the five content clusters, CVR values ranged from 0.50 to 1.00, with an overall mean CVR of 0.69 ± 0.12. The highest levels of agreement were observed for cultural appropriateness and metaphors (mean CVR = 0.88) and content accuracy and clinical relevance (mean CVR = 0.86), indicating strong consensus that the materials were both scientifically sound and contextually appropriate. Clarity and readability also scored highly (mean CVR = 0.71). Lower but still acceptable CVRs were seen for illustration and layout quality (mean CVR = 0.59) and flow and organisation (mean CVR = 0.55). Comments in these domains related mainly to preferences about font size, white space, and the ordering of some examples rather than concerns about the underlying content. Engagement and tone and perceived feasibility and implementation utility showed mean CVRs of 0.64 and 0.50, respectively, suggesting that panellists generally viewed the materials as engaging and perceived them as potentially feasible, while also highlighting areas where additional implementation support (e.g., training, integration into clinic routines) might be helpful. These ratings represent stakeholder judgement rather than direct feasibility or usability testing. Table 2 presents CVR values across domains and clusters.

Final PNE structure and features: The final Urdu PNE framework comprised ten concise modules arranged for progressive learning. Each module paired a core pain-science principle with a culturally resonant metaphor, supported by illustrations and brief clinician prompts. Table 3 presents the final Urdu PNE framework, showing each module, its key concept, and the culturally adapted metaphor.

Clinician notes suggested ways to integrate the concepts into brief consultations (e.g., using one metaphor per visit, linking explanations to patients’ existing beliefs, and reinforcing active self-management strategies). Panellist feedback indicated that the metaphors felt recognisable, the language was understandable, and the structure could be flexibly adapted for use in different clinical settings (e.g., physiotherapy, general practice, exercise rehabilitation).

## 4. Discussion

This study describes the development, cultural adaptation, and content validation of Urdu-language pain neuroscience education (PNE) materials for individuals with low back pain and for use by healthcare professionals in Pakistan. Using a multistep adaptation process, the final ten-module booklet and clinician guide achieved acceptable to high levels of content validity across all evaluated domains. These findings indicate that the adapted materials were considered clear, relevant, and culturally appropriate by a multidisciplinary panel and may offer a useful resource for supporting pain-related education in clinical and community settings [26,27].

The development process involved integrating contemporary pain-science concepts with illustrations, examples, and language familiar to Urdu-speaking users. Focus group participants emphasised the need for simple, everyday wording and advised avoiding technical terminology where possible. They also recommended using metaphors and examples reflecting common routines and communication patterns in Pakistani households. These suggestions guided several refinements, including simplifying sentence structures, modifying illustrations to align with local norms, and selecting examples that would be easily understood across varying literacy levels. This pattern is consistent with previous PNE adaptation studies in Turkish- and Hausa-speaking communities, where participants similarly highlighted the importance of culturally familiar metaphors, appropriate visual content, and straightforward explanations [10,11,12].

Culturally grounded metaphors were a central component of the adaptation. Pakistani communication practices commonly incorporate figurative storytelling and are shaped by collectivist social norms, family-based narrative traditions, and faith-oriented coping responses [28,29,30]. Metaphors such as “House in Rain” or “Over-Protective Guard” mirror these familiar narrative structures and align with local ways of explaining health and adversity. Prior research on South Asian pain narratives shows that concrete, relatable imagery enhances comprehension and engagement [31,32], supporting the inclusion of these metaphors within the adapted Urdu materials and reinforcing their conceptual fit for the target population [33].

The quantitative content-validation stage provided further support for the clarity and relevance of the adapted materials. The overall mean content validity ratio (CVR = 0.69 ± 0.12) exceeded the minimum threshold for a panel size of 32, indicating that raters generally viewed the content as essential for inclusion [16,26]. The highest agreement was observed for cultural appropriateness and content accuracy, consistent with findings from the Turkish and Hausa adaptations [10,11,12], which also reported strong agreement in these domains. Evaluators in the present study noted that the metaphors, illustrations, and Urdu phrasing felt suitable for explaining PNE concepts within local contexts. Although the illustration and layout domains received comparatively lower but still acceptable ratings, comments suggested that these related mainly to preferences for formatting (e.g., spacing, colour contrast) rather than concerns about the images themselves. Similar observations have been described in other studies developing educational materials for use in low- and middle-income countries [34].

The adapted PNE materials were judged clear and culturally appropriate, suggesting potential applicability in clinical communication; however, their effectiveness and real-world uptake remain to be tested. Participants in the focus groups indicated that the modules could be incorporated into brief consultations to support discussions about pain, activity, and recovery expectations. The clinician guide was viewed as helpful for maintaining consistency in messaging and for introducing key topics in a stepwise manner [35,36,37]. Contemporary evidence and guidelines emphasise active physiotherapy and self-management over passive modalities for chronic LBP [38]. The Urdu PNE modules were designed to align with this direction by reinforcing the safety of movement (*Motion Is Medicine*). While the present study did not evaluate clinical outcomes associated with using the materials, the positive content-validation findings suggest that they are acceptable and easy to understand for their intended audience. These characteristics are important prerequisites for future studies assessing the effectiveness of PNE interventions [39].

The process used in this study aligns with recommended frameworks for cross-cultural adaptation of health education materials. Forward–backward translation ensured linguistic equivalence [13], while involvement of clinicians and a person living with low back pain helped identify unclear or inappropriate content early in the process [9]. The combination of qualitative feedback and quantitative validation strengthened the overall development process and helped confirm that the adapted content was both conceptually accurate and culturally appropriate. This approach is consistent with methods used in similar PNE adaptation work in other non-English-speaking communities [10,11,12,28].

Several strengths of this study should be noted. First, it used a structured, multistage adaptation process that incorporated perspectives from multiple stakeholder groups, including physiotherapists, medical professionals, exercise practitioners, and individuals with lived experience of low back pain. This multidisciplinary involvement enhanced the diversity of viewpoints considered during refinement [40]. Second, the sample size for content validation (*n* = 32) was larger than that used in some previous adaptation studies [10,11,12], providing more stable estimates of content validity [15,16]. Third, the development of both a patient-facing booklet and a clinician guide increases the practical utility of the materials, as it supports consistent communication between healthcare providers and patients [36,41].

The study also has limitations. Content validation was conducted in a single round, whereas some adaptation frameworks recommend additional rounds of expert review or Delphi procedures to further refine consensus [28]. Future work may therefore consider multiple rounds of validation, particularly for illustration and layout components. A key limitation is limited patient co-design during qualitative refinement. As noted earlier, due to the contributions of only one patient with LBP, the resultant tool may not fully capture patient priorities regarding wording, metaphors, illustrations, and acceptability across diverse literacy levels and sociocultural contexts in Pakistan. In the quantitative phase, the content validation panel included 10 people with LBP out of 32 raters; therefore, essential ratings may reflect clinician priorities more than end-user preferences. Future work should include larger and more diverse patient samples in co-design and module-level usability testing [9]. The study did not evaluate clinical outcomes associated with using the Urdu PNE materials, so their impact on pain beliefs, fear-avoidance, or disability remains unknown [39]. Effectiveness studies, including pre-post evaluations or randomised trials, are needed to determine whether these materials influence patient outcomes when used in routine practice [39]. Additionally, although Urdu is widely understood across Pakistan, it is not the first language for all communities. Future adaptations into regional languages such as Punjabi, Sindhi, Pashto, and Balochi may help broaden reach and accessibility [42].

Future research should examine how best to integrate these materials into clinical workflows and whether brief training for clinicians enhances consistent delivery [35,36]. Studies exploring patient engagement, comprehension, and preferences for receiving pain-related information would also be valuable [43]. Also, CVR ratings were collected at the cluster level rather than per module, and domain scores may obscure variability between individual modules. Future studies should undertake module-level content validity and end-user usability testing. Further refinement of illustrations, layout, or optional multimedia formats (e.g., audio or video versions) may support use among low-literacy populations [44], although such formats were outside the scope of the present work.

## 5. Conclusions

The present study used a structured, multistep process to develop and culturally adapt pain neuroscience education materials in Urdu for people with low back pain and for use by healthcare professionals in Pakistan. Through translation, qualitative refinement, and quantitative preliminary content validation, the final five cluster booklets and clinician guides were judged to be clear, relevant, and culturally appropriate by a multidisciplinary panel. These findings indicate that the adapted materials are acceptable to intended users and may provide a practical resource to support pain-related education in clinical and community settings. Future work should evaluate clinical feasibility and behavioural outcomes to determine the real-world effectiveness of these Urdu PNE materials.

## Figures and Tables

**Table 1:** Characteristics of the content validation panel (*n* = 32).

Characteristic	Category	n (%) or Mean ± SD
Stakeholder group	Total	32 (100)
	Physiotherapists	8 (25.0)
	Medical practitioners	6 (18.8)
	Exercise professionals	8 (25.0)
	Individuals with LBP	10 (31.2)
Clinical experience (years)	HCPs managing back pain (*n* = 22)	9.4 ± 5.8 years
Age (years)	Overall panel	41.2 ± 10.6
	Healthcare professionals	39.8 ± 9.7
	Individuals with LBP	44.1 ± 11.9
Back pain duration (years)	Individuals with LBP (*n* = 10)	6.3 ± 4.1

**Table 2:** Content validity ratios (CVRs) across domains and clusters (*n* = 32).

Validation Domain	Cluster 1	Cluster 2	Cluster 3	Cluster 4	Cluster 5	Mean CVR (±SD)	Interpretation
Content accuracy and clinical relevance	0.75	0.875	0.875	0.9375	0.875	0.86 ± 0.07	High agreement
Clarity and readability	0.625	0.75	0.75	0.75	0.6875	0.71 ± 0.05	Strong
Cultural appropriateness and metaphors	0.6875	0.9375	0.875	1.00	0.875	0.88 ± 0.11	Very high agreement
Illustration and layout quality	0.5625	0.625	0.625	0.625	0.50	0.59 ± 0.05	Acceptable
Flow and organisation	0.50	0.5625	0.5625	0.5625	0.5625	0.55 ± 0.02	Acceptable
Engagement and tone	0.4375	0.6875	0.6875	0.6875	0.6875	0.64 ± 0.10	Strong
Perceived feasibility and utility	0.375	0.50	0.50	0.5625	0.5625	0.50 ± 0.07	Acceptable perceived feasibility

Mean overall CVR = 0.69 ± 0.12 (threshold for *n* = 32 = 0.30, *p* < 0.05).

**Table 3:** Final Urdu pain neuroscience education (PNE) Framework.

Module	Key Concept	Urdu Metaphor
1	Understanding Pain—Pain is protection, not damage	گھر بارش میں (House in Rain)
2	Mind–Body Connection—Fear and thoughts influence pain	چوکیدار زیادہ محتاط (Over-Protective Guard)
3	Motion Is Medicine—Gentle movement teaches safety	زنگ لگا دروازہ (Rusting Door)
4	Healthy Habits—Sleep, diet, and stress affect pain	توازن کی ترازو (Balance Scale)
5	Recovery and Hope—Brains can re-learn safety	روشنی کا سفر (Journey of Light)
6	Self-care—Supported by personal values/faith	ایمان اور عمل (Faith and Action)
7	Communication—Words as medicine in healing	الفاظ کا مرہم (Words as Medicine)
8	Family Support—Encouragement reduces fear	گھر کی طاقت (Strength of Family)
9	Movement Confidence—Gradual exposure builds trust	پگڈنڈی راستہ (Path to Confidence)
10	Relapse and Resilience—Flare-ups are learning, not failure	سفر جاری ہے (Journey Continues)

## Data Availability

The original contributions presented in this study are included in the article/Supplementary Material. Further inquiries can be directed to the corresponding author.

## Supplementary Materials

The following supporting information can be downloaded at: https://www.mdpi.com/article/10.3390/medsci14010054/s1, S1: PNE Urdu Booklet; S2: PNE English Booklet; S3: Clinician guide PNE Pakistan.

## Abbreviations

The following abbreviations are used in this manuscript:

LBP	Low back pain
PNE	Pain neuroscience education
CVR	Content validity ratio
