# Supplementary material for: Development, Cultural Adaptation, and Content Validation of Urdu Pain Neuroscience Education Materials for Low Back Pain in Pakistan
Source: Med Sci (Basel). 2026 Jan 22;14(1):54. doi: 10.3390/medsci14010054 (PMC12921984; doi:10.3390/medsci14010054)
Supplement: Supplementary file 1 [file medsci-14-00054-s001.zip › S1 - PNE booklet - Urdu.pdf]

کلسٹر 1 – درد کو سمجھنا

درد ≠ نقصان

بارش میں گھر

ذرا تصور کریں کہ آپ کا گھر تیز مون سون بارش میں ہے۔ بارش زور سے برس رہی ہے، گرج چمک ہو رہی ہے، اور اچانک گھر کا الارم بج اٹھتا ہے۔

آپ فوراً گھبرا کر چیک کرنے جاتے ہیں - لیکن چھت مضبوط ہے، دروازے بند ہیں، دیواریں ٹھیک ہیں۔ مسئلہ گھر میں نہیں... الارم بس زیادہ حساس ہوگیا ہے۔

آپ کے جسم کا درد کا نظام بھی کبھی کبھی اسی طرح کام کرتا ہے۔ اس کی اصل ذمہ داری تحفظ ہے، لیکن بعض اوقات یہ بہت تیزی سے بجنے لگتا ہے , حتیٰ کہ جب آپ کا "گھر" یعنی آپ کا جسم بالکل محفوظ ہو۔

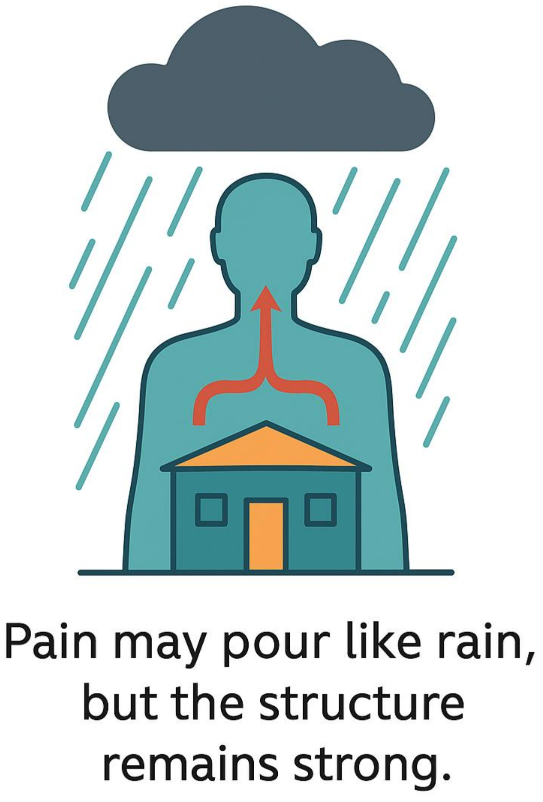

اصل میں درد ہے کیا؟

درد کسی نقصان کی سیدھی نشانی نہیں ہوتا۔ یہ ایک تحفظ کا اشارہ ہوتا ہے۔ آپ کا جسم ہر سیکنڈ میں بے شمار پیغامات دماغ تک بھیجتا ہے۔ دماغ ان پیغامات کو سنتا ہے، سمجھتا ہے، اور فیصلہ کرتا ہے کہ درد پیدا کرنا ہے یا نہیں۔

جب اسے خطرہ محسوس ہو تو یہ سگنل کو تیز کر دیتا ہے، اور جب اسے حفاظت محسوس ہو تو یہ سگنل کو کم کر دیتا ہے۔

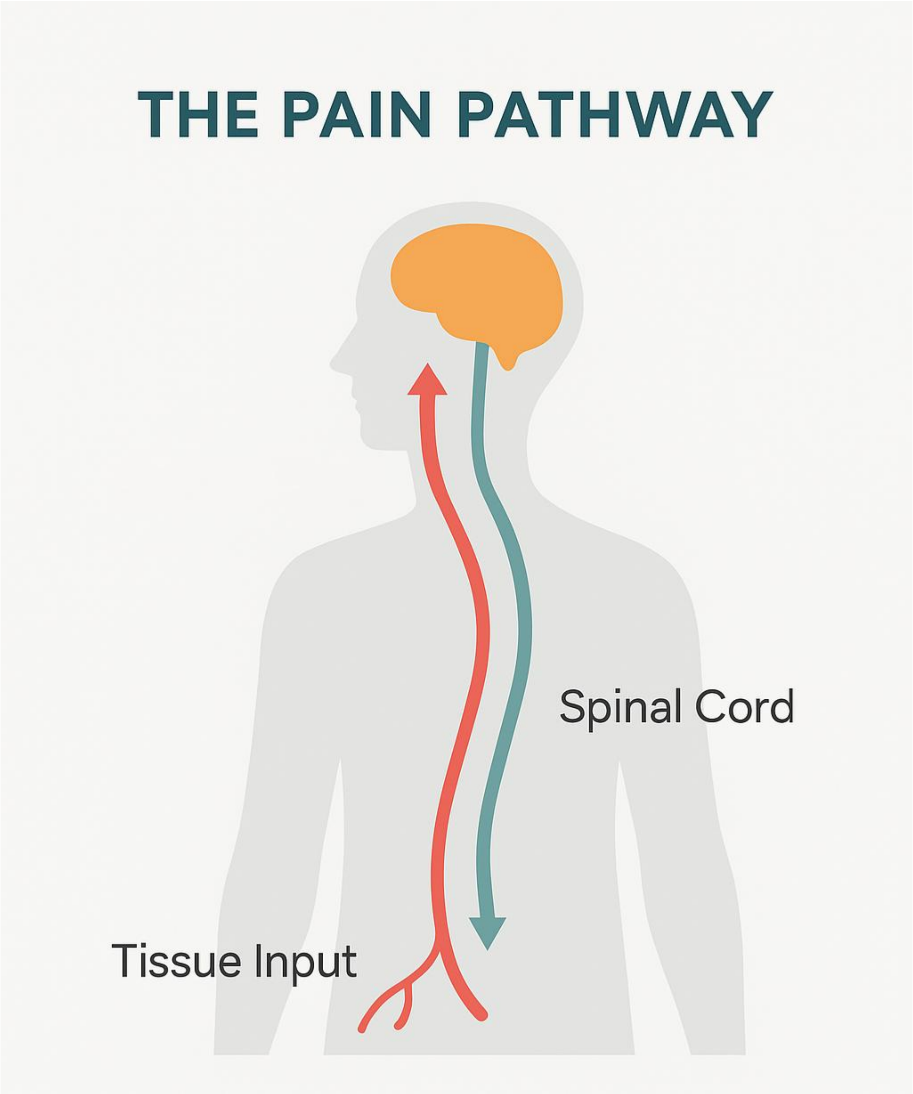

درد ایک ایسا پیغام ہے جو اعصابی نظام کے ہر درجے پر بن کر شکل اختیار کرتا ہے۔

<u>جب جسم کا حفاظتی الارم بند ہی نہ ہو</u>

چوٹ کے بعد کبھی ایسا ہوتا ہے کہ اعصاب اور دماغ کے حصے جاگے ہوئے رہتے ہیں، حالانکہ جسم کے ٹشوز پوری طرح ٹھیک ہو چکے ہوتے ہیں۔

اس کیفیت کو سینسِٹائزیشن کہا جاتا ہے۔ یعنی الارم ضرورت سے زیادہ محتاط ہو گیا ہے۔ اس کا یہ مطلب نہیں کہ کوئی نیا نقصان ہو رہا ہے۔ بلکہ اس کا مطلب ہے کہ آپ کا نظام آپ کی حفاظت بہت زیادہ کرنے لگ گیا ہے۔

اچھی خبر؟ جو چیز سیکھ لی جاتی ہے، اسے بدلا بھی جا سکتا ہے۔

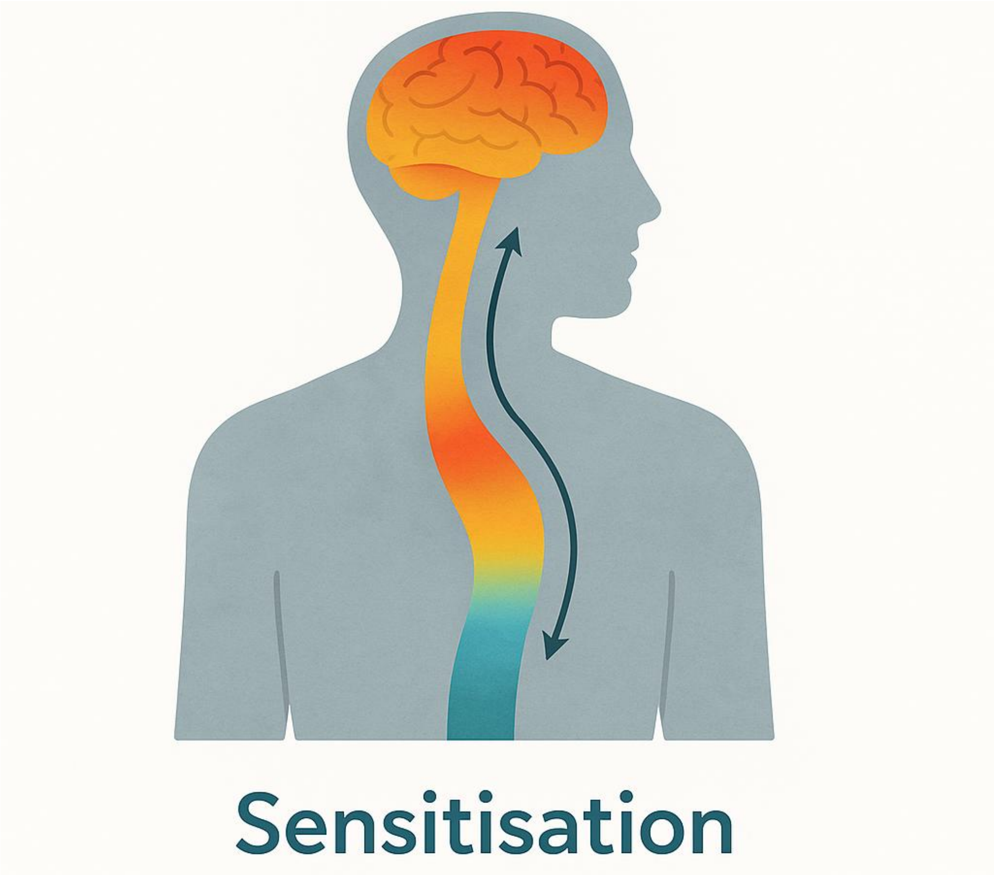

حساسیت بڑھ سکتی ہے، لیکن اس نظام کو دوبارہ سکھایا اور بہتر کیا جا سکتا ہے۔

<u>سمجھنے سے کیسے مدد ملتی ہے</u>

یہ جاننا کہ درد نقصان نہیں، آپ کو زیادہ اعتماد سے حرکت کرنے میں مدد دیتا ہے۔
سمجھ بوجھ خود ہی اعصابی نظام کے "خطرے کے سگنلز" کو کم کر دیتی ہے۔
جب بھی آپ درد کے بارے میں کوئی نیا تصور سیکھتے ہیں، دماغ اپنا "سیفٹی فائل" اپ ڈیٹ کرتا ہے۔
خوف کم ہونا ہی شفا کی طرف پہلا قدم ہے۔

آج یہ آزمائیں

1. جب درد بڑھ جائے تو خود سے پوچھیں: “کیا میں واقعی خطرے میں ہوں... یا صرف زیادہ حساس ہو گیا ہوں؟”
2. تین آہستہ، گہرے سانس لیں اور کہیں: “میرا جسم مضبوط ہے، اور میں سکون سیکھ رہا ہوں۔”
3. دو منٹ ہلکی چہل قدمی کریں - صرف ایک محفوظ سگنل بھیجنے کے لیے۔
4. اپنی کمر کے بارے میں ایک حوصلہ افزا جملہ لکھیں اور آج رات اونچی آواز میں پڑھیں۔

اپنے معالج سے بات کریں

- ان سے پوچھیں کہ آپ کے کیس میں “حساسیت بڑھنا” کیا معنی رکھتا ہے۔
- اس ہفتے کون سی محفوظ سرگرمیاں دوبارہ شروع کی جا سکتی ہیں، یہ ساتھ بیٹھ کر طے کریں۔

خلاصہ پیغام

درد ایک الارم کی طرح ہے - حقیقی، لیکن کبھی کبھی بہت زیادہ اونچی آواز والا۔
آپ کا جسم مضبوط ہے، آپ کا نظام حفاظتی ہے، اور درد کو سمجھنا ہی پہلی دوا ہے۔

کلسٹر 2 – ذہن اور جسم کا تعلق

خیالات، احساسات اور درد ایک ساتھ کام کرتے ہیں

زیادہ محتاط چوکیدار

ذرا تصور کریں کہ آپ کے گھر کے باہر ایک چوکیدار کھڑا ہے۔ وہ برا نہیں، بلکہ آپ کی حفاظت کرنا چاہتا ہے۔

لیکن پچھلے کچھ عرصے سے وہ ضرورت سے زیادہ چوکنا ہو گیا ہے۔ اب ہلکی سی آواز , بلی کی چلنے کی، یا ٹرک کے گزرنے کی , پر بھی وہ فوراً الارم بجا دیتا ہے۔ چوکیدار کی نیت خراب نہیں ہوتی , وہ بس حد سے زیادہ خبردار رہنے لگا ہے۔

مسلسل درد میں جسم کا نظام بھی اسی طرح کام کر سکتا ہے۔ آپ کا اعصابی نظام، یعنی جسم کا چوکیدار, جب بھی اسے ذرا سا بھی خطرہ محسوس ہوتا ہے . تو وہ تحفظ کے لیے سگنل بڑھا دیتا ہے , خواہ اصل خطرہ موجود نہ بھی ہو۔ آپ کے خیالات، خوف اور جذبات اس چوکیدار کو کبھی پُرسکون اور کبھی بے چین بنا سکتے ہیں۔

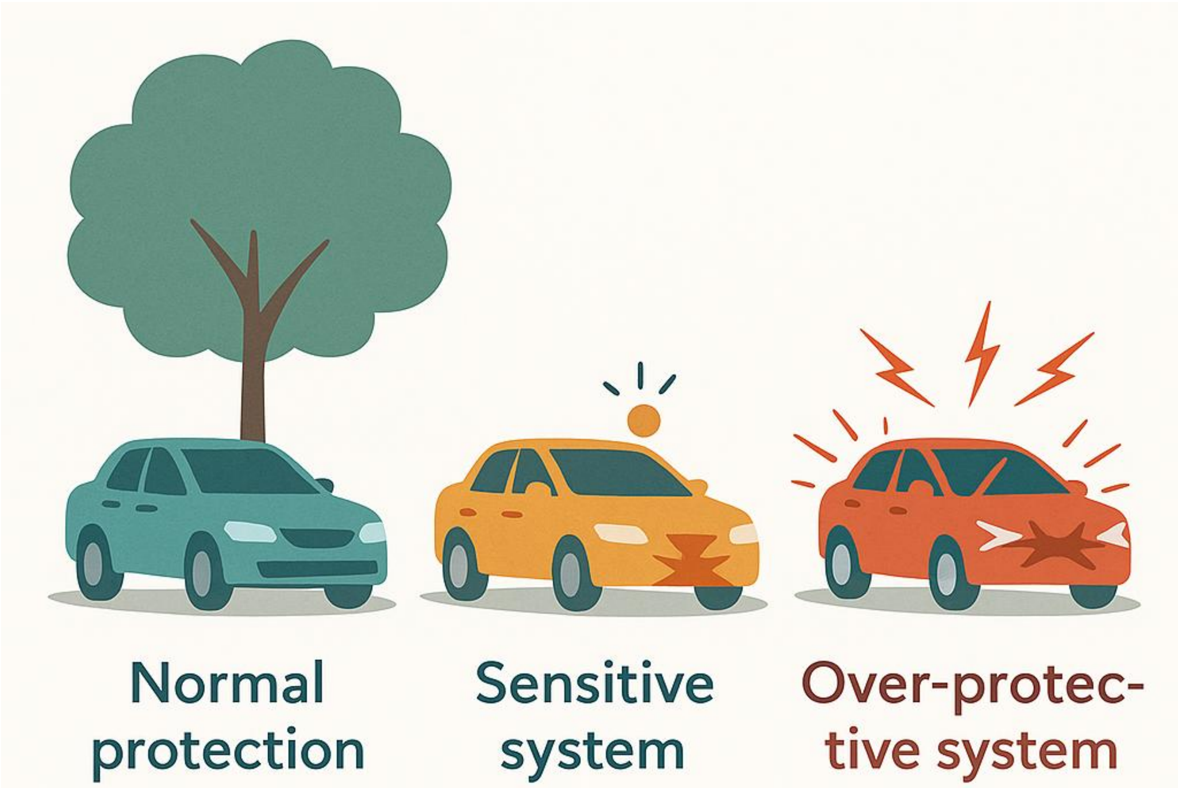

ضرورت سے زیادہ محتاط الارم اُس وقت بھی بج سکتا ہے جب کوئی خطرہ موجود نہ ہو۔

ذہن اور جسم ایک دوسرے سے کیسے بات کرتے ہیں

- جسم مختلف قسم کے سگنلز دماغ تک بھیجتا رہتا ہے۔
- دماغ فیصلہ کرتا ہے کہ یہ سگنلز خطرے کی نشانی ہیں یا محفوظ صورتحال کی۔
- اگر دماغ کو نقصان یا خطرے کی توقع ہو تو درد بڑھ جاتا ہے۔
- اور اگر اسے اعتماد اور حفاظت محسوس ہو تو درد کم ہو جاتا ہے۔

خوف، غصہ اور فکر اس "آواز" کو تیز کر سکتے ہیں، جبکہ امید، سکون اور یقین اسے آہستہ کر دیتے ہیں۔

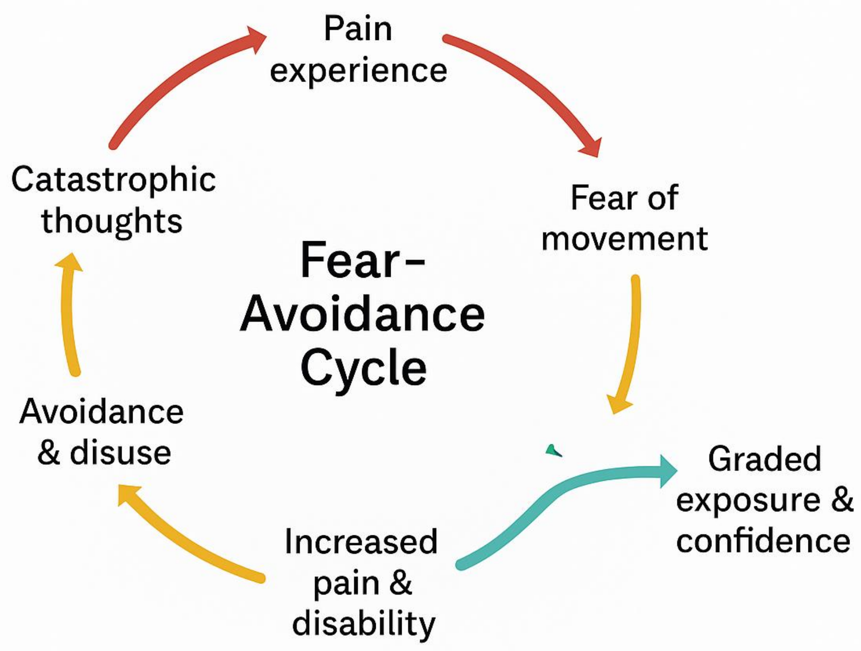

Fear strengthens the loop; confidence breaks it.

خوف اور درد کے چکر کو کیسے توڑا جائے

جب ہمیں درد کا ڈر ہوتا ہے تو ہم عام طور پر کم حرکت کرتے ہیں۔ کم حرکت سے جسم سخت، کمزور اور زیادہ حساس ہو جاتا ہے۔ اسی کو خوف اور پرہیز کا چکر کہا جاتا ہے۔

اس چکر سے نکلنے کا واحد راستہ ہلکی، آہستہ آہستہ بڑھائی جانے والی حرکت ہے ۔ ایسی حرکت جو آپ کے اعصابی نظام کو یہ ثابت کرے کہ آپ محفوظ ہیں، اور آپ کا جسم حرکت برداشت کر سکتا ہے۔

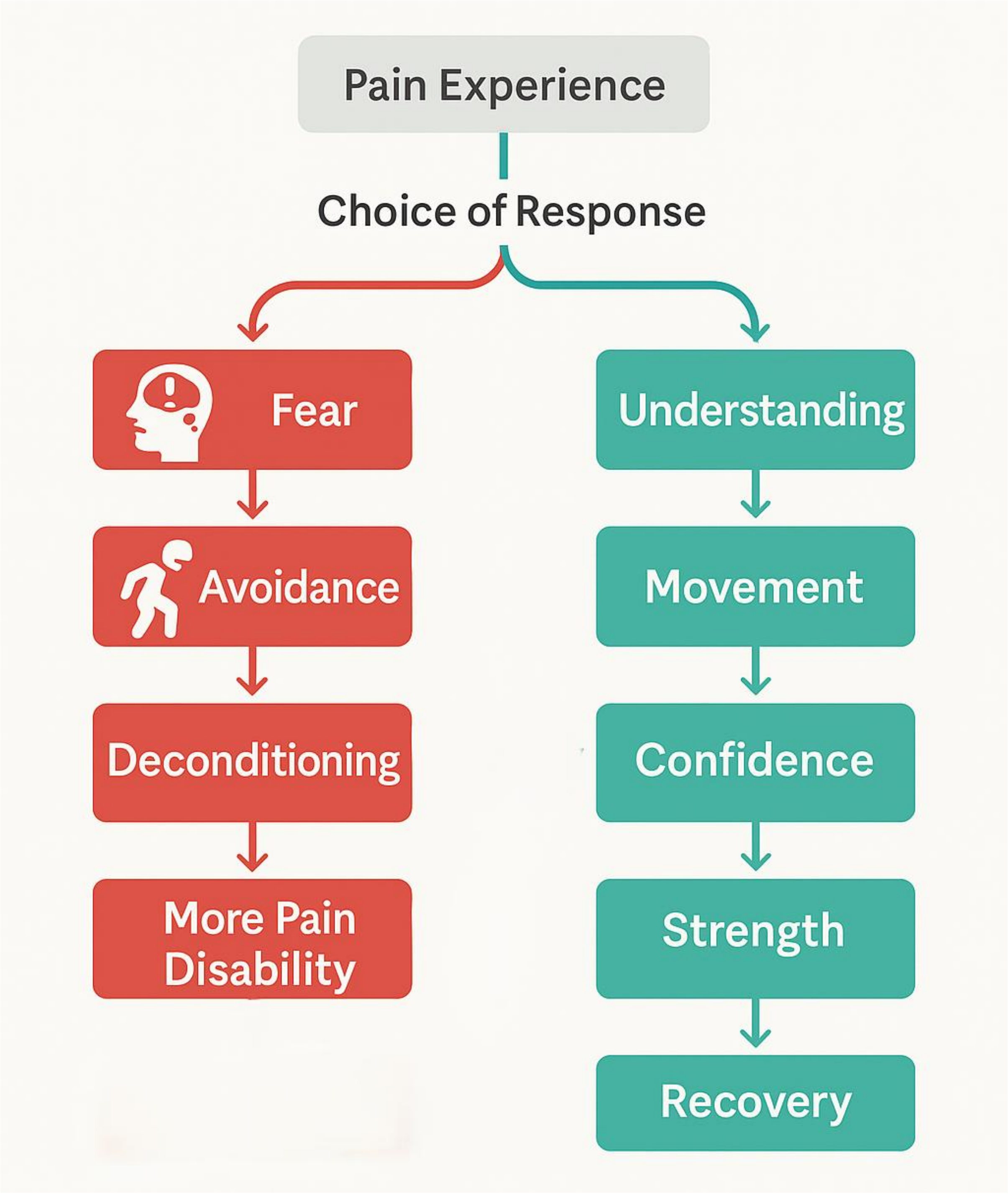

دو راستے بنتے ہیں: خوف درد کو بڑھاتا ہے، اعتماد زندگی کو دوبارہ بناتا ہے۔

چوکیدار کو کیسے پرسکون کیا جائے

- علم: یہ سمجھ لینا کہ درد نقصان نہیں۔
- حرکت: ہلکی سی حرکت چوکیدار کو پیغام دیتی ہے کہ "سب ٹھیک ہے۔"
- سانس لینا: آہستہ، گہرے سانس الارم کے سگنلز کو کم کرتے ہیں۔
- سپورٹ: گھر والوں یا معالج کے حوصلہ افزا الفاظ خطرے کے احساس کو کم کرتے ہیں۔

حتیٰ کہ تھوڑی سی بہتری - موڈ میں، امید میں، یا اعتماد میں دماغ کو الارم آہستہ کرنے میں مدد دیتی ہے۔

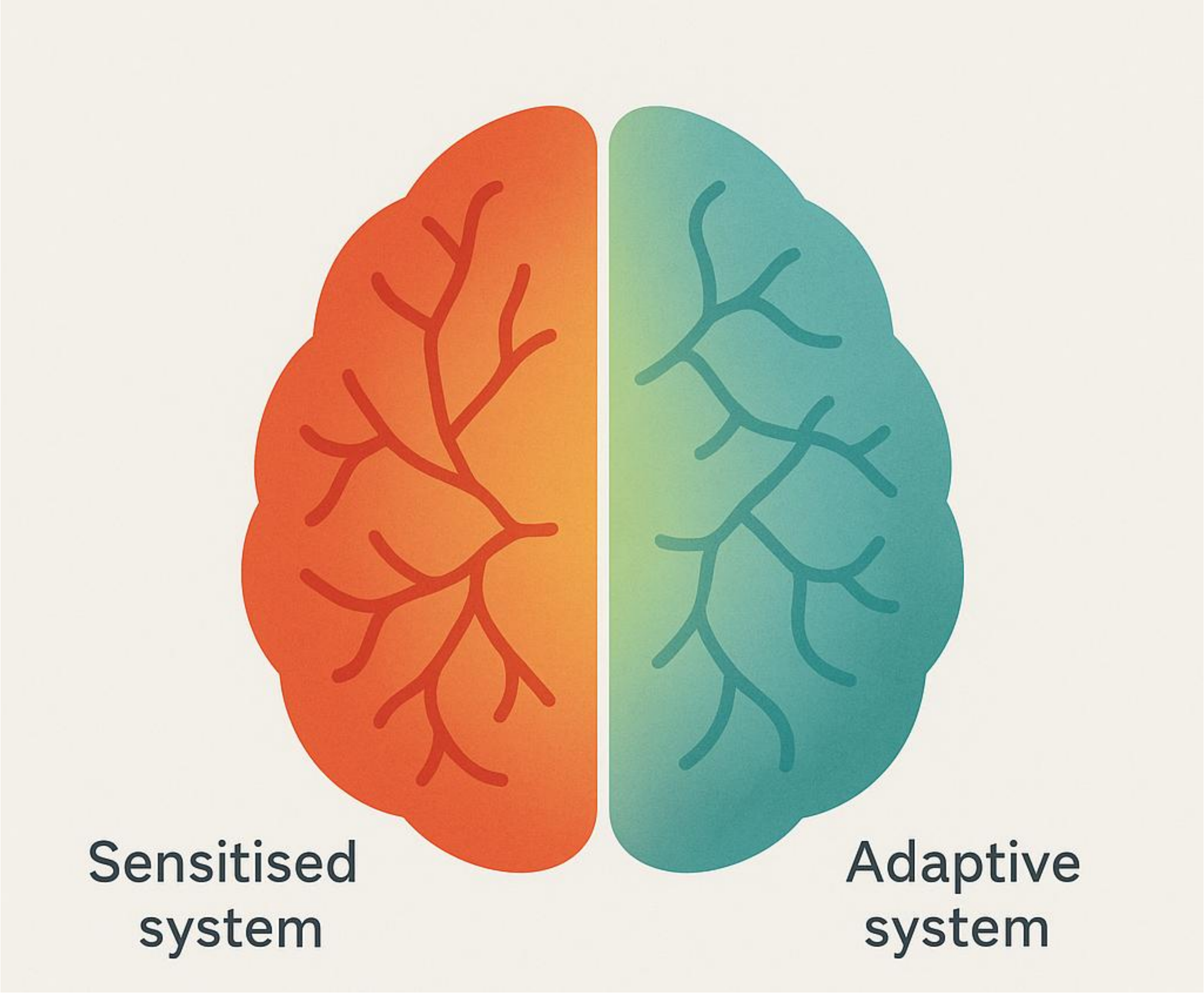

سیکھنے اور پرسکون خیالات سے نظام دوبارہ ایسی سمت میں بنتا ہے جو حفاظت کو مضبوط کرتی ہے۔

آج یہ آزمائیں

1. جب درد محسوس ہو تو آہستہ سے خود سے کہیں "میں محفوظ ہوں، میرا جسم سیکھ رہا ہے"۔

2. اپنے جسم کا وہ چھوٹا سا حصہ حرکت دیں جس سے آپ بچ رہے تھے - ہلکی سی اسٹریچ، ہلکی موڑ، یا نرم جھکاؤ۔

3. ناک کے ذریعے تین آہستہ، گہرے سانس لیں اور صرف سکون پر توجہ رکھیں۔

4. حرکت کے بارے میں اپنا ایک خوف لکھیں، پھر اس کے سامنے ایک حقیقت لکھیں جو اس خوف کو چیلنج کرتی ہو۔

اپنے معالج سے بات کریں

- پوچھیں: کون سی حرکتیں میں دوبارہ محفوظ طریقے سے شروع کر سکتا ہوں؟
- انہیں وہ ڈر بتائیں جو آپ کو چلنے پھرنے یا کام کرنے سے روکتا ہے۔
- ان سے گریڈیڈ ایکسپوژر کے بارے میں سیکھنے کی درخواست کریں - یعنی اعتماد کو آہستہ آہستہ دوبارہ کیسے بنایا جائے۔

خلاصہ پیغام

درد جسم اور ذہن دونوں میں محسوس ہوتا ہے۔ خوف درد کو تیز کرتا ہے، جبکہ اعتماد اسے کم کرتا ہے۔
آپ اپنے نظام کو دوبارہ محفوظ محسوس کرنا سکھا سکتے ہیں۔

کلسٹر 3 – حرکت ہی دوا ہے

ہر قدم آپ کے جسم کو "محفوظ" ہونا سکھاتا ہے

زنگ لگا ہوا دروازہ

ذرا ایک ایسے دروازے کا سوچیں جو کئی مہینوں سے نہیں کھولا گیا۔ نہ حرکت، نہ استعمال - نتیجہ؟ اس کے قبضوں میں زنگ لگ جاتا ہے۔

پھر جب آپ اسے پہلی بار دھیرے سے دھکا دیتے ہیں تو وہ چرچراتا ہے۔ یہ اس لیے نہیں کہ دروازہ ٹوٹا ہوا ہے بلکہ اس لیے کہ وہ لمبے عرصے سے ہلا نہیں۔ پھر جب آپ اسے چند بار آہستہ آہستہ کھولتے اور بند کرتے ہیں تو وہ دوبارہ آسانی سے چلنے لگتا ہے۔

آپ کی کمر بھی بالکل ایسے ہی کام کرتی ہے۔ جب خوف یا درد کی وجہ سے حرکت رک جائے تو جسم سخت اور زیادہ حساس ہو جاتا ہے۔ ہلکی، نرمی سے کی گئی حرکت - قبضوں میں تیل ڈالنے کی طرح ہے یہ جسم کے نظام کو یاد دلاتی ہے کہ آپ مضبوط ہیں اور حرکت محفوظ ہے۔

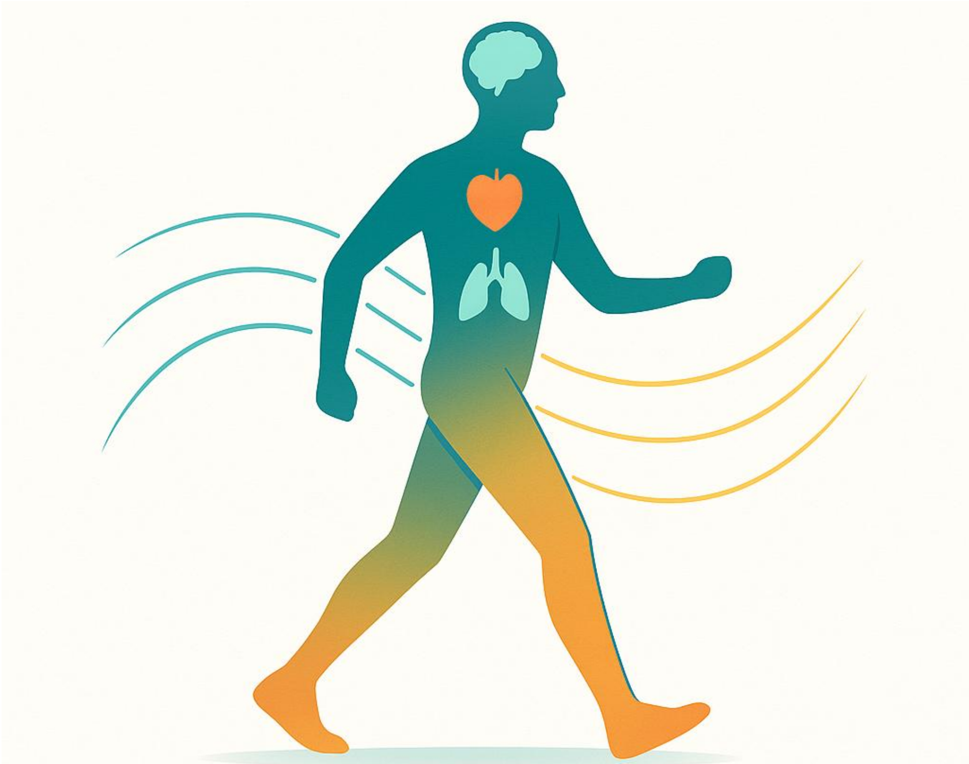

حرکت سکون اور اعتماد واپس لاتی ہے۔

کیوں حرکت دوا کی طرح کام کرتی ہے

- ”حرکت دماغ کو یہ محفوظ پیغام بھیجتی ہے کہ “جسم کا یہ حصہ ٹھیک ہے، نقصان میں نہیں ہے۔
- حرکت سے خون کی روانی بڑھتی ہے پٹھے نرم ہوتے ہیں اور سکون دینے والے کیمیکلز (اینڈورفن) جسم میں جاری ہوتے ہیں۔
- دماغ دوبارہ سیکھتا ہے کہ جھکنا، بیٹھنا، نماز کے رکوع و سجود، یا چلنا پھرنا سب محفوظ اور قابلِ اعتماد حرکات ہیں۔

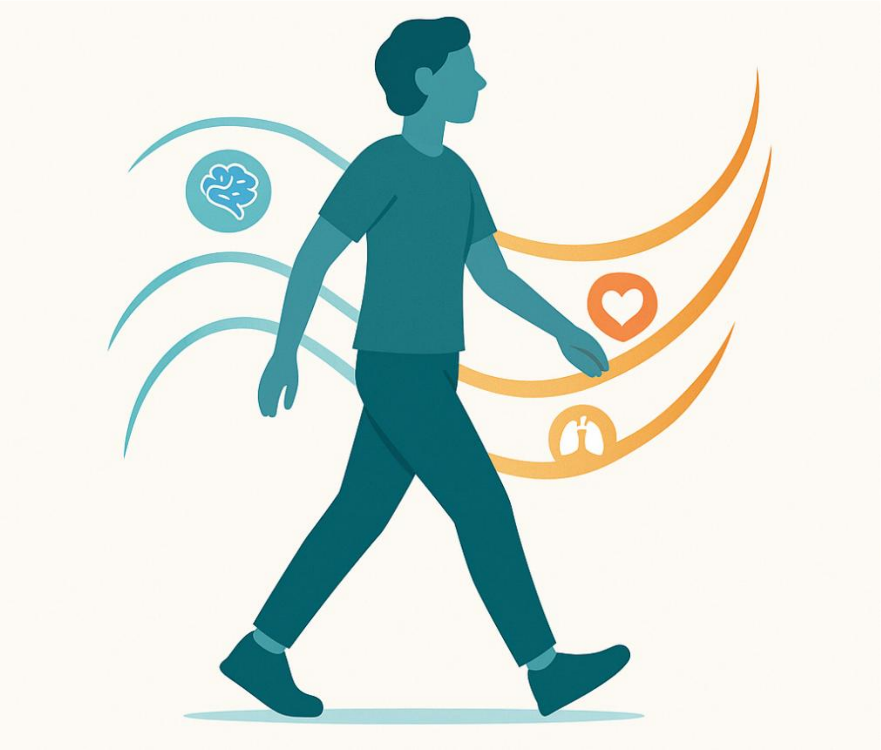

تھوڑا سا چلنا پھرنا بھی آپ کی ٹھیک ہونے کی رفتار بڑھا دیتا ہے۔

مثال

بہت سے لوگ کہتے ہیں : آرام کروں گا تو ٹھیک ہو جاؤں گا۔ لیکن زیادہ دیر کا آرام نظام کو کمزور اور زیادہ خوفزدہ کر دیتا ہے۔

جیسے ہماری روزانہ کی نمازوں میں باقاعدہ حرکات شامل ہوتی ہیں اسی طرح جسم کو بھی تسلسل اور روانی کی ضرورت ہوتی ہے۔ نرم، باقاعدہ جسمانی حرکت عبادت (جسم کی نگہداشت) اور شکر (جسم کی صلاحیت کا اعتراف) دونوں کا حصہ ہے۔

محفوظ طریقے سے کیسے چلیں پھریں

1. آغاز کریں: روز گھر کے اندر صرف 5 منٹ چہل قدمی کریں۔
2. سنبھالیں: درد بڑھنے سے پہلے وقفہ لیں، بعد میں نہیں۔
3. سانس اور حرکت ساتھ رکھیں: آہستہ سانس لیتے ہوئے نرمی سے حرکت کریں۔
4. آہستہ آہستہ اضافہ کریں: ہر دن ایک منٹ یا کوئی ہلکی اسٹریچ بڑھا دیں۔
5. اپنی پیشرفت نوٹ کریں: ہر رات ایک چھوٹی کامیابی لکھیں، جیسے: میں آج 10 منٹ آرام سے بیٹھ سکا۔

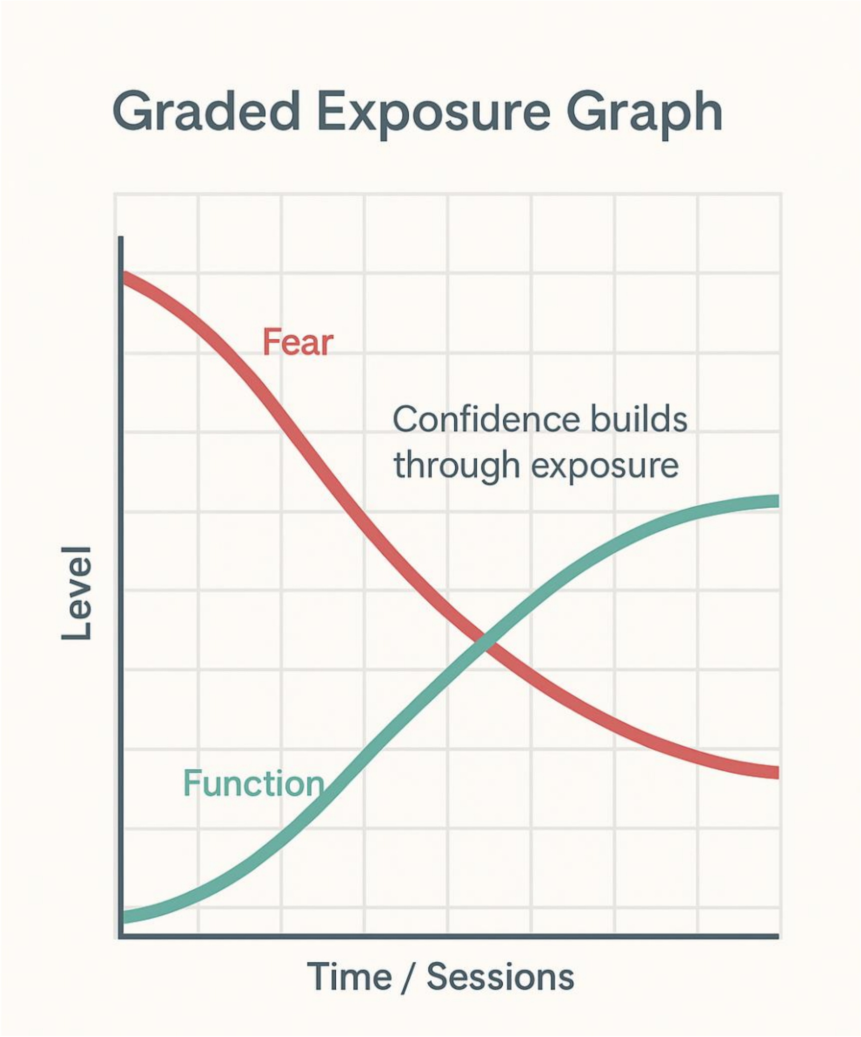

جب آپ چیزوں کو محفوظ طریقے سے بار بار آزماتے ہیں تو اعتماد بڑھتا جاتا ہے۔

آج یہ آزمائیں

- ہر نماز کے بعد 2 منٹ آہستہ چہل قدمی کریں اور ہلکی اسٹریچ کریں۔
- جب درد محسوس ہو تو خود سے کہیں: "یہ میرا نظام حفاظت سیکھ رہا ہے۔"
- "میں نہیں کر سکتا" کی جگہ "میں نرمی سے شروع کروں گا" کہیں۔
- اپنی حرکت کے اہداف کسی دوست یا گھر والے سے شیئر کریں تاکہ حوصلہ ملے۔

اپنے معالج سے بات کریں

- ان سے کہیں کہ وہ آپ کے لیے ورزش کا منصوبہ بنائیں
- انہیں بتائیں کہ کون سی حرکات سے آپ سب سے زیادہ ڈرتے ہیں وہ آپ کو محفوظ طریقے سے دوبارہ شروع کرنا سکھا سکتے ہیں۔
- پیسنگ پر بات کریں: یعنی زیادہ کرکے تھک جانے اور پھر کئی دن کچھ نہ کرنے کے چکر سے کیسے بچنا ہے۔

خلاصہ پیغام

حرکت شفا ہے۔ ہر نرم قدم الارم کو دھیما کرتا ہے اور اعتماد بناتا ہے۔ آپ کا جسم حرکت کے لیے بنایا گیا ہے اور حرکت ایک امانت ہے جسے سنبھال کر رکھنا ضروری ہے۔

کلسٹر 4 – صحت مند عادات اور سپورٹ

چھوٹی روزمرہ عادتیں الارم کو پرسکون کر سکتی ہیں

مصروف گھر

گھروں میں صبح کا وقت ہمیشہ حرکت اور سرگرمی سے بھرا ہوتا ہے ناشتہ بن رہا ہوتا ہے، بچے اسکول کی تیاری میں اور بڑے دفتر جانے کے لیے مصروف ہوتے ہیں۔

لیکن جب گھر میں کوئی ایک فرد بیمار یا کمزور ہو جائے تو پورا گھر اپنا معمول بدل لیتا ہے۔ اکثر گھر والے پیار سے کہتے ہیں: زیادہ مت چلو پھرو، حالت خراب ہو جائے گی۔ ان کی نیت اچھی ہوتی ہے مگر ضرورت سے زیادہ حفاظت کبھی کبھی صحت یابی کو روک بھی دیتی ہے۔

شفا تب بہتر ہوتی ہے جب آپ کے آس پاس کے لوگ یہ مانیں کہ آپ دوبارہ مضبوط ہو سکتے ہیں اور وہ آپ کو اعتماد اور ہمت دیں نہ کہ ڈر اور پرہیز۔

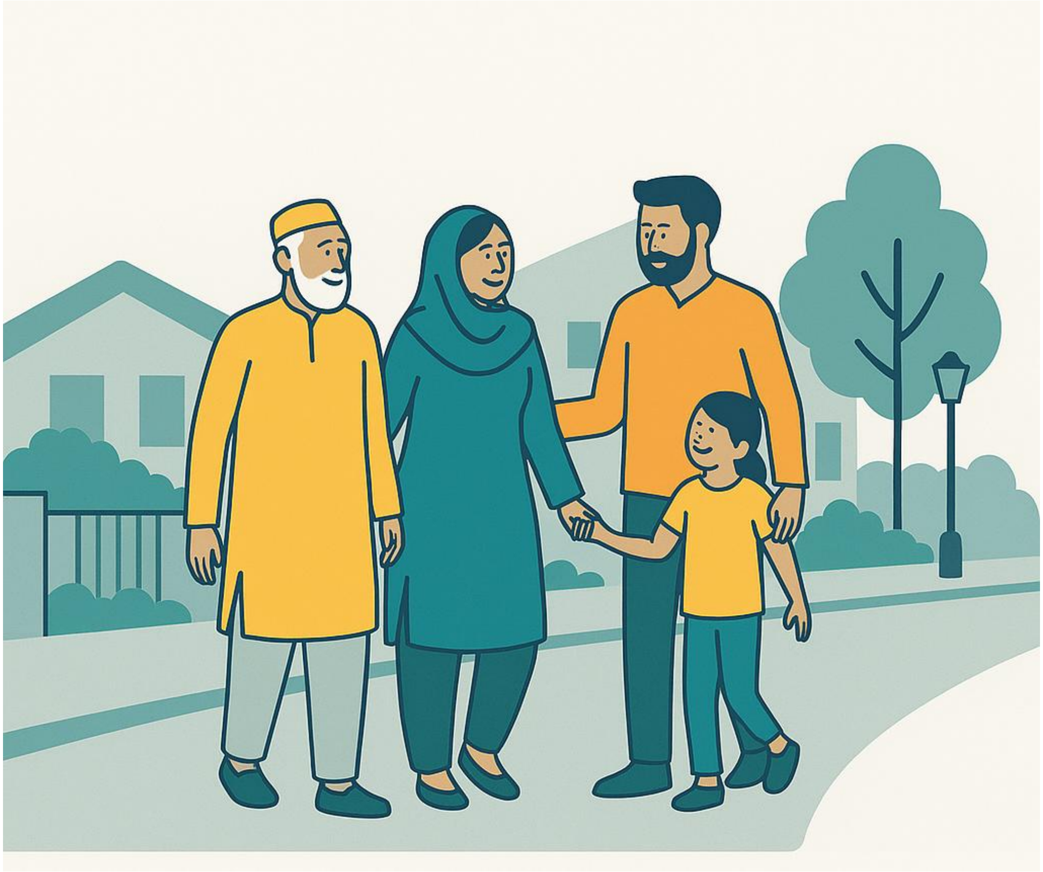

لوگوں کا ساتھ صحت یابی کو آسان بناتا ہے۔

نیند اور درد

اچھی نیند دوا کی طرح کام کرتی ہے۔ جب نیند پوری نہ ہو تو درد زیادہ تیز محسوس ہوتا ہے توانائی کم ہو جاتی ہے اور ذہن میں ڈَر بڑھ جاتا ہے۔

نیند بہتر کرنے سے درد کی حساسیت کم ہو سکتی ہے۔

کوشش کریں

- ہر رات ایک ہی وقت پر سونے کی کوشش کریں۔
- سونے سے پہلے بھاری کھانا، کیفین، اور موبائل فون سے پرہیز کریں۔
- لیٹنے سے پہلے تین گہرے سانس لیں، "الحمدللہ" کہیں اور اپنے جسم کے پٹھوں کو آہستہ آہستہ ڈھیلا کریں۔

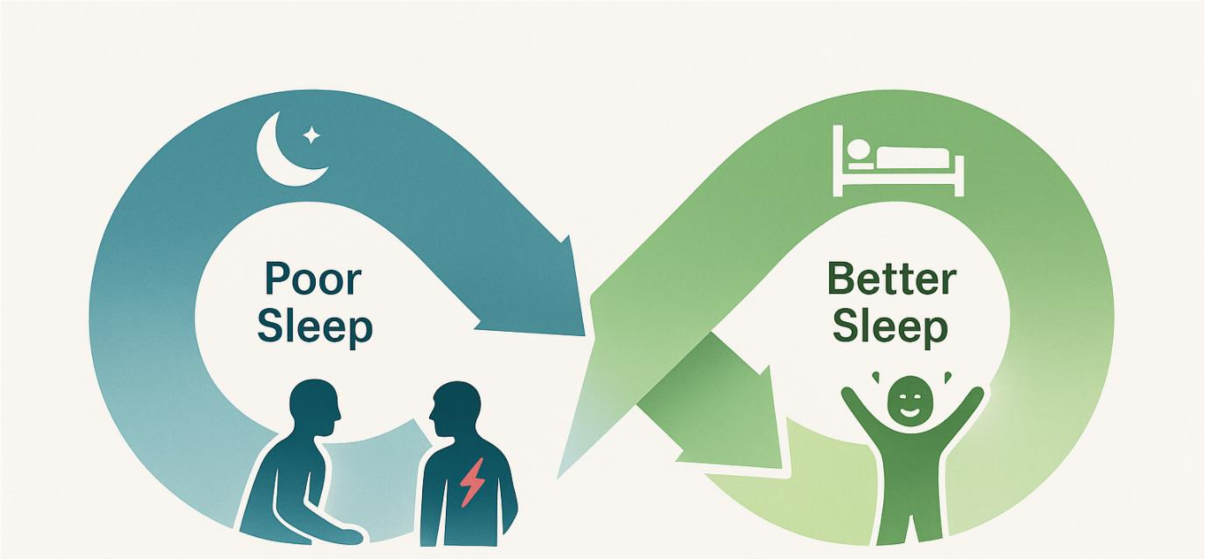

جب یہ چکر ٹوٹتا ہے تو جسم اور ذہن دونوں میں توازن بحال ہوتا ہے۔

تناؤ اور اعصابی نظام

تناؤ کے ہارمون آپ کے جسم کے الارم سسٹم کو مسلسل چلتا ہوا رکھتے ہیں۔ مالی پریشانی، گھر کے جھگڑے یا درد کی فکر یہ سب آپ کی حساسیت کو بڑھا سکتے ہیں۔

لیکن پرسکون روٹینز جیسے نماز، گہرے سانس، اور باہر قدرت میں کچھ وقت گزارنا آپ کے جسم کے "آرام اور مرمت" نظام کو آن کر دیتے ہیں۔

یاد رکھیں: تناؤ کم کرنا علاج کا حصہ ہے، کمزوری نہیں۔

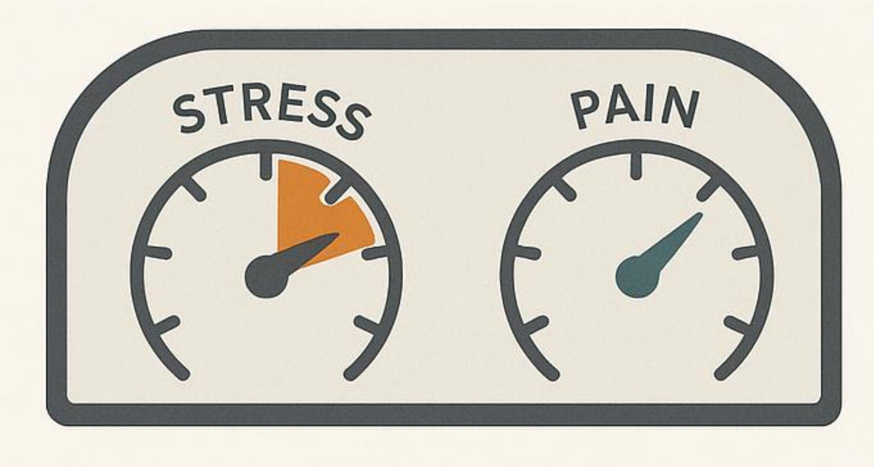

آپ کا درد کا ڈیش بورڈ روزمرہ عادتوں سے بہتر ہو سکتا ہے۔

کھانا اور شفا

آپ جو کھاتے ہیں، وہ آپ کے جسم کی کیمسٹری پر اثر ڈالتا ہے۔
پھل ، سبزیاں، دالیں، میوہ جات، مچھلی، اور ہول گرین سوزش کو کم کرتے ہیں۔ زیادہ شکر، تیل، یا پراسیسڈ کھانا سوزش کو بڑھا دیتا ہے۔

آپ کو کوئی "خاص ویسٹرن ڈائٹ" کی ضرورت نہیں۔ بس متوازن کھانے اعتدال میں رکھ کر کھائیں۔

مثال

- پراٹھے میں زیادہ تیل کی جگہ اولیو آئل یا کینولا آئل استعمال کریں۔
- روز ایک وقت میں دال، سبزی یا کوئی پھل ضرور شامل کریں۔
- پانی مناسب مقدار میں پئیں پانی کی کمی پٹھوں کو سخت اور درد کو زیادہ محسوس کرواتی ہے۔
- لمبے کام یا تناؤ کے دوران کھانا مت چھوڑیں۔

الفاظ بھی دوا ہیں

لوگ آپ کے بارے میں جو الفاظ بولتے ہیں، وہ شفا بھی دے سکتے ہیں اور نقصان بھی پہنچا سکتے ہیں۔

تمہاری کمر ختم ہو گئی ہے - سننے سے خوف اور درد بڑھتا ہے۔ جبکہ "تمہارا جسم مضبوط ہے اور ٹھیک ہو رہا ہے" ہمت، امید اور اعتماد پیدا کرتا ہے۔

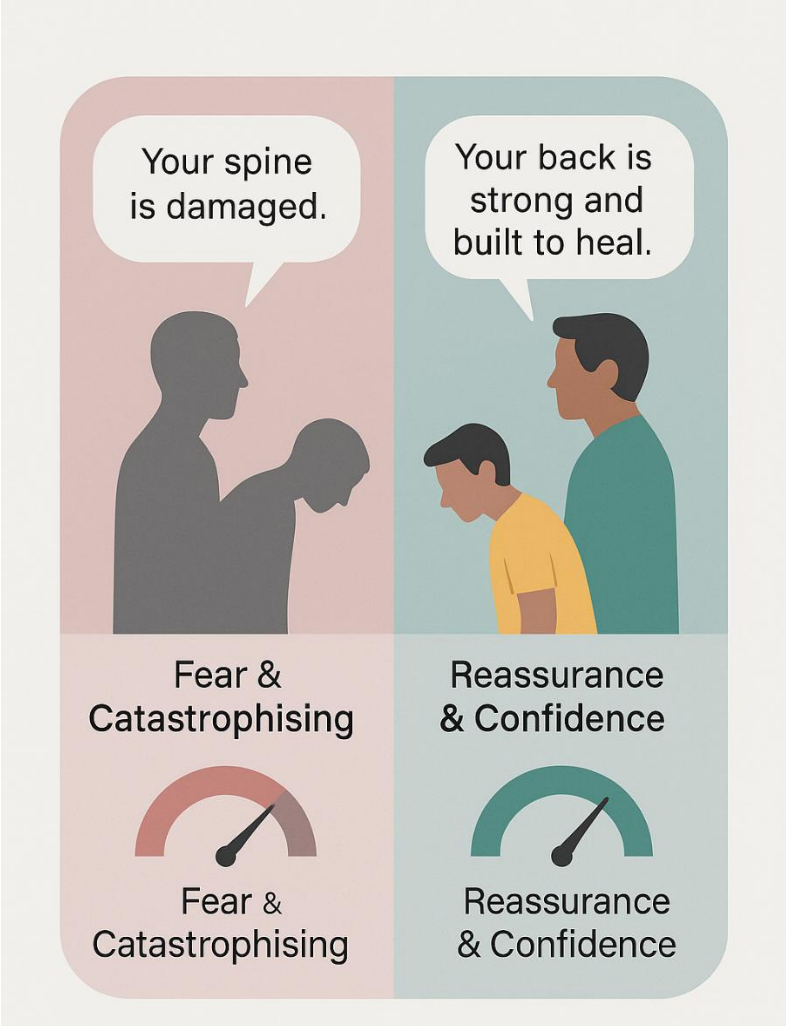

زبان شفا بھی دے سکتی ہے اور نقصان بھی

گھر والوں کو حوصلہ افزا الفاظ استعمال کرنے کی ترغیب دیں ۔ مثلاً: "تم بہتر ہو رہے ہو " بجائے "یہ درد کبھی نہیں جائے گا"۔ ایسے الفاظ دل کو مضبوط کرتے ہیں اور درد کو کم محسوس کرواتے ہیں۔

خاندان اور کمیونٹی کی سپورٹ

پاکستان میں خاندان قریبی ہوتے ہیں اور یہ ایک نعمت ہے۔ جب گھر والے درد کو صحیح طرح سمجھتے ہیں، تو وہ صحت یابی میں شریک بن جاتے ہیں۔

- ان سے کہیں کہ صرف تیمار داری نہ کریں , آپ کے ساتھ چلیں بھی۔
- انہیں بتائیں کہ کون سے الفاظ آپ کو ہمت دیتے ہیں۔
- انہیں اپنی ورزش یا ریلیکسیشن ٹائم میں شامل کریں۔

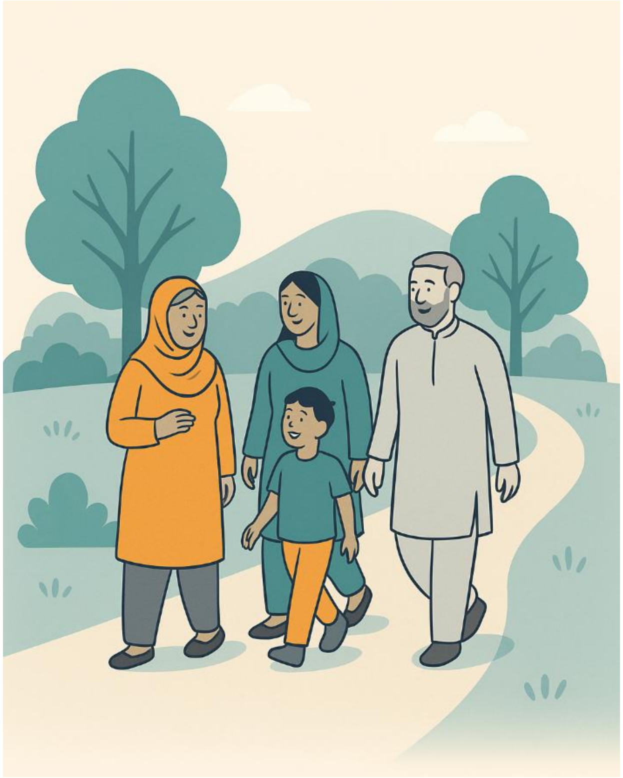

شفا تعلق اور ربط سے بڑھتی ہے

آج یہ آزمائیں

1. رات کے کھانے کے بعد کسی گھر والے کے ساتھ 5 منٹ چہل قدمی کریں۔
2. ایک منفی جملے کی جگہ حوصلہ افزا جملہ استعمال کریں۔
3. آج ایک ایسا کھانا کھائیں جس میں رنگ ہو , جیسے تازہ سبزیاں، دال، یا کوئی پھل۔
4. دن میں دو منٹ خاموشی سے دعا، سانس یا مراقبہ کریں۔
5. سونے سے 30 منٹ پہلے موبائل فون بند کر دیں۔

اپنے معالج سے بات کریں

- انہیں اپنی نیند، کھانے پینے یا تناؤ کی مشکلات بتائیں , یہ سب دوا جتنے ہی اہم ہیں۔
- پوچھیں کہ مصروف دنوں یا روزوں کے دوران محفوظ اور معتدل ورزش کیسے کی جا سکتی ہے۔
- گھر والے آپ کی پیسنگ اور سرگرمی کے اہداف میں کیسے مدد کر سکتے ہیں، اس پر بھی گفتگو کریں۔

خلاصہ پیغام

شفا خاندان اور کمیونٹی کے ساتھ مل کر بڑھتی ہے۔ نیند، تناؤ، الفاظ، کھانا، اور ایمان , یہ سب مل کر آپ کے جسم کے الارم سسٹم کو متوازن کرتے ہیں۔ جب آپ کا گھر، دل، اور عادات ایک ساتھ کام کریں تو درد خود بخود ہلکا اور پرسکون ہو جاتا ہے۔

کلسٹر 5 – بحالی اور امید

آپ اپنے نظام کو دوبارہ سکھا سکتے ہیں

طوفان کے بعد کی صبح

ایک طویل رات کی مسلسل بارش کے بعد باالآخر سورج نکلتا ہے۔ دیواریں نم ہوتی ہیں، لیکن گھر اپنی جگہ مضبوط کھڑا رہتا ہے۔ آپ کھڑکیاں کھولتے ہیں، تازہ ہوا اندر آتی ہے، اور گھر آہستہ آہستہ خشک اور بہتر ہونے لگتا ہے۔

آپ کا درد کا سفر بھی کچھ ایسا ہی ہے۔ طوفان آتے ہیں- خوف، سختی، تناؤ- لیکن وہ ہمیشہ گزرتے ہیں۔ جسم اور دماغ دوبارہ سکون سیکھ سکتے ہیں، بالکل ویسے جیسے آسمان ہر طوفان کے بعد صاف ہونا سیکھ لیتا ہے۔

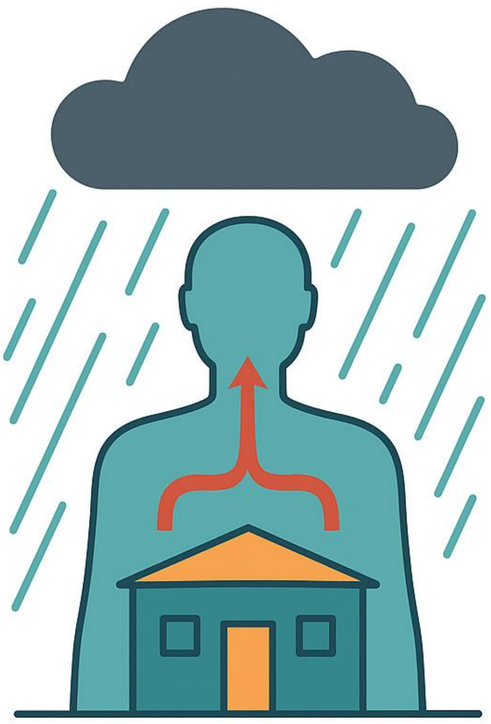

درد بارش کی طرح برس سکتا ہے، مگر بنیاد پھر بھی مضبوط رہتی ہے۔

شفا کے تین مراحل

شفا کوئی جادو نہیں - یہ **تربیت** ہے۔ آپ اپنے اعصابی نظام کو چھوٹے مگر مسلسل عمل سے دوبارہ سکھا سکتے ہیں۔

شفا کو تین ایسے مرحلوں کے سفر کی طرح سمجھیں

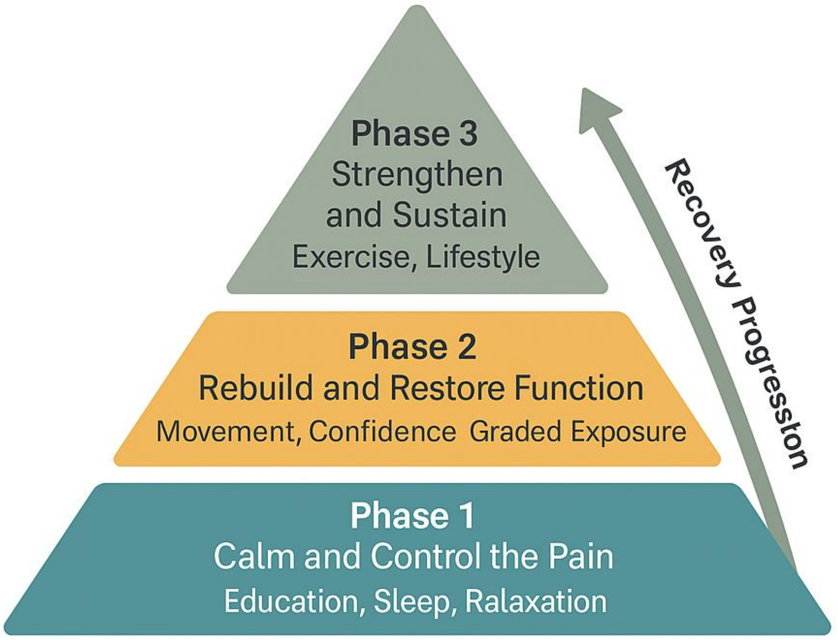

محفوظ ہونے سے مضبوط ہونے تک کا سفر

1. درد کو پرسکون کریں اور کنٹرول حاصل کریں. درد کے بارے میں سیکھیں؛ سمجھداری سے آرام کریں اور گہرے سانس لیں۔ نیند بہتر کریں؛ نرمی سے حرکت کریں۔ خود سے کہیں: میں محفوظ ہوں۔

2. بحالی اور کارکردگی : باقاعدگی سے چلنا یا اسٹریچنگ شروع کریں۔ چھوٹی، محفوظ سرگرمیوں کے ذریعے خوف کو آہستہ آہستہ چیلنج کریں۔ یقین رکھیں: میرا جسم سیکھ رہا ہے۔

3. مضبوطی پیدا کریں اور اسے برقرار رکھیں : ورزش کریں، صحت مند کھائیں، اور لوگوں سے جڑے رہیں۔ سرگرمی اور آرام کے درمیان توازن رکھیں۔ سوچیں: میں یہ سنبھال سکتا ہوں۔

غیرفعال سے فعال بحالی کی طرف سفر

بہت سے لوگ انتظار کرتے ہیں کہ کوئی دوسرا انہیں ٹھیک کر دے۔ لیکن اصل شفا تب ہوتی ہے جب آپ اپنی صحت یابی میں خود حصہ لیتے ہیں۔ فعال حصہ لینا ہی آپ کے اعصابی نظام کو دوبارہ سکون، طاقت، اور اعتماد سکھاتا ہے۔

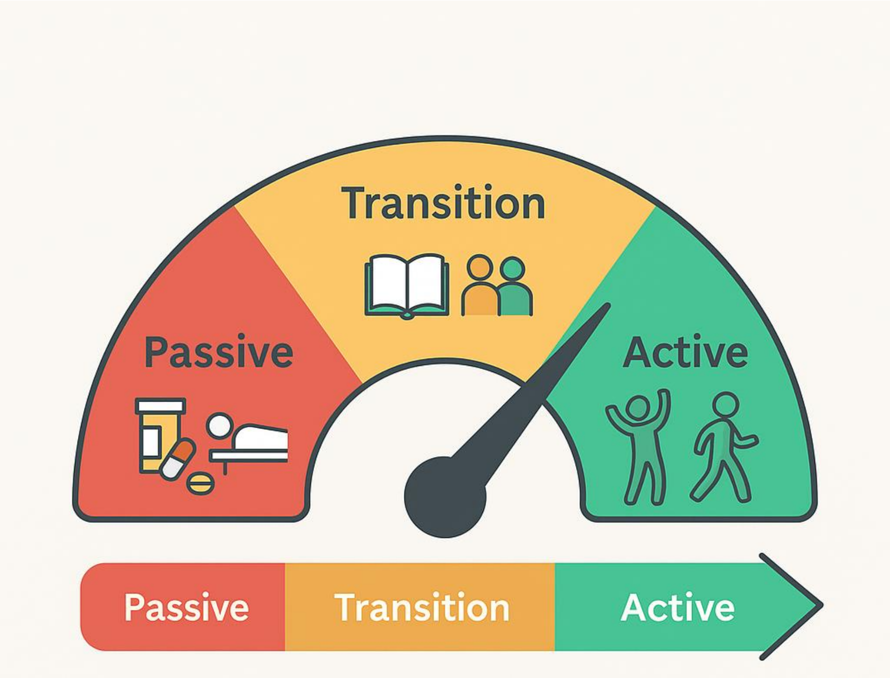

انحصار سے شمولیت کی طرف قدم بڑھائیں

- غیرفعال علاج - جیسے دوائیں، انجیکشن، یا بستر پر رہنا عارضی سکون دے سکتے ہیں۔
- فعال علاج - جیسے علم حاصل کرنا، حرکت کرنا، اور پیسنگ دیرپا تبدیلی لاتے ہیں۔
- جب آپ اپنے مددگار خود بنتے ہیں تو آپ کا دماغ بھی بدلنے لگتا ہے۔ دوا فائدہ دیتی ہے لیکن دعا اور عمل مل کر اسے مؤثر بناتے ہیں۔

دماغ کیسے شفا پاتا ہے

درد صرف پٹھوں یا ہڈیوں میں نہیں رہتا - یہ زیادہ تر اعصابی نظام میں بن اور بدل رہا ہوتا ہے۔

جب آپ اعتماد کے ساتھ حرکت کرتے ہیں، مثبت سوچتے ہیں، اور درد کے بارے میں نیا علم سیکھتے ہیں، تو دماغ اپنے راستے دوبارہ بناتا ہے - اسی عمل کو نیورو پلاسٹیسٹی کہا جاتا ہے۔ یعنی دماغ سیکھنے کے ذریعے خود کو زیادہ محفوظ، مضبوط، اور پُرسکون بنا سکتا ہے۔

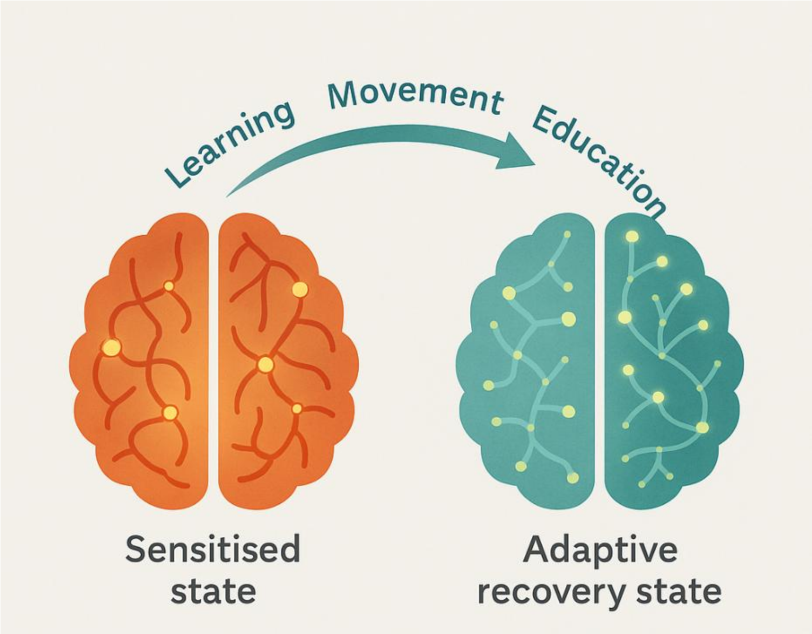

آپ کا ہر مثبت خیال، ہر ہلکی اسٹریچ، ہر چلنا پھرنا، اور ہر دعا دماغ میں نئے "محفوظ" راستے بناتی ہے۔

ایمان اور مضبوطی

درد آپ کے صبر کو آزماتا ہے، لیکن یہی آزمائش آپ کی قوت کو بھی گہرا کر سکتی ہے۔ اسلام ہمیں صبر بھی سکھاتا ہے اور عمل بھی۔ آپ بے بس نہیں ہیں آپ کی کوشش شفا کا حصہ ہے۔

بے شک مشکل کے ساتھ آسانی ہے۔" (سورۃ الشرح 94:6)"

جب آپ درد کے باوجود چلتے ہیں، سانس لیتے ہیں، مسکراتے ہیں، یا کسی کی مدد کرتے ہیں، تو آپ اپنے آپ کو ثابت کر رہے ہوتے ہیں کہ امید، تکلیف سے زیادہ طاقتور ہے۔

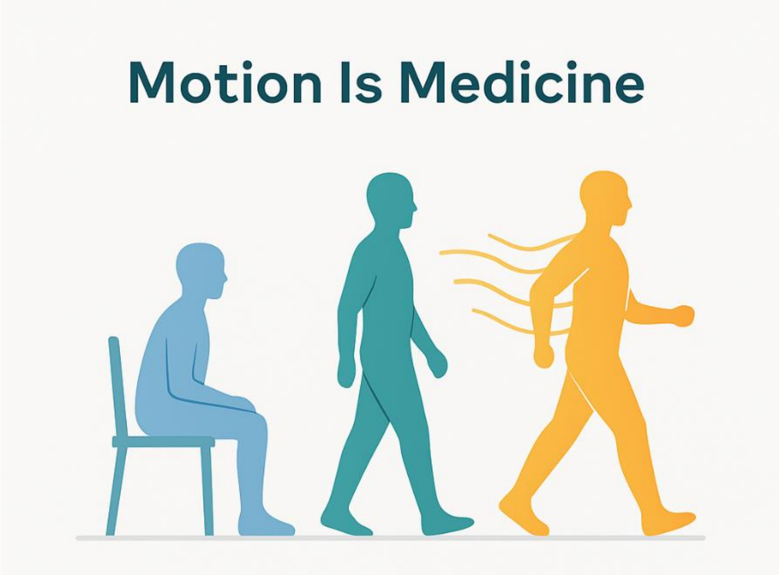

آج یہ آزمائیں

1. آج کل کی نسبت پانچ منٹ زیادہ چلیں۔
2. "میں ٹوٹا ہوا ہوں" کی جگہ کہیں: "میں دوبارہ بن رہا ہوں۔"
3. ہر صبح اللہ کا شکر کریں کسی ایک عضو کے لیے جو اچھی طرح کام کر رہا ہے۔
4. کسی ایک شخص کی مدد کریں - یہ عمل آپ کی اپنی شفا کو بھی مضبوط کرتا ہے۔
5. سونے سے پہلے تصور کریں کہ آپ کے جسم کا الارم آہستہ آہستہ پرسکون ہو رہا ہے۔

اپنے معالج سے بات کریں

- اپنے روزانہ کے اہداف بتائیں اور پیسنگ و ورزش پر ان کی رائے لیں۔
- پوچھیں کہ دوبارہ درد بڑھنے سے بچنے کے طویل مدتی طریقے کیا ہو سکتے ہیں۔
- اگر دوبارہ تکلیف آنے کا ڈر ہو تو اس پر بات کریں۔ مل کر مشکل دنوں کا ایک سادہ پلان بنائیں۔

خلاصہ پیغام

بحالی ممکن ہے - انتظار سے نہیں، شمولیت سے۔ درد بدل سکتا ہے کیونکہ دماغ بدل سکتا ہے۔ ایمان، علم، اور حرکت مل کر شفا کی بنیاد رکھتے ہیں۔ جیسے ہر سورج کی پہلی کرن اندھیری رات کے بعد آتی ہے، اسی طرح ہر شفا کا سفر امید سے شروع ہوتا ہے۔
