# Supplementary material for: Development, Cultural Adaptation, and Content Validation of Urdu Pain Neuroscience Education Materials for Low Back Pain in Pakistan
Source: Med Sci (Basel). 2026 Jan 22;14(1):54. doi: 10.3390/medsci14010054 (PMC12921984; doi:10.3390/medsci14010054)
Supplement: Supplementary file 1 [file medsci-14-00054-s001.zip › S2 - PNE booklet - English.pdf]

<u>Cluster 1 – Understanding Pain</u>

Pain ≠ Damage

The House in the Rain

Imagine your home during a heavy monsoon.
Rain pours, thunder rolls, and the alarm suddenly rings.
You rush to check, but the roof is strong, the doors are locked, the walls safe.
The alarm isn't wrong, it's just too sensitive.
Your body's pain system can work the same way.
It's there to protect you, but sometimes it rings loudly even when your house is safe.

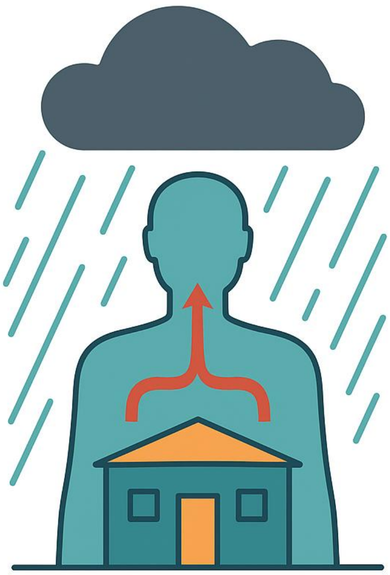

Pain may pour like rain,
but the structure
remains strong.

What Pain Really Is?

Pain is not a direct measure of damage. It's a protective signal.
Your body sends many messages to the brain every second.
The brain listens, interprets, and decides when to produce pain.
It can turn the signal up when it feels danger or down when it feels safe.

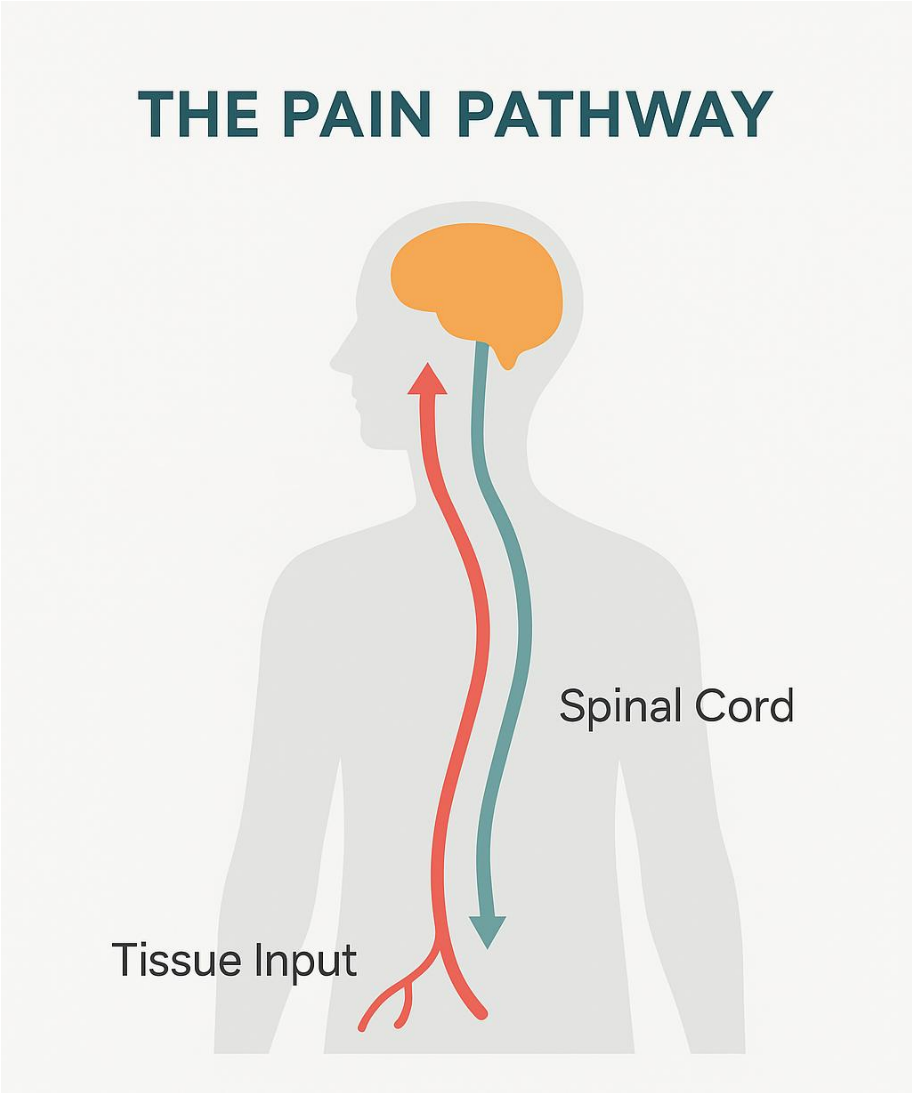

Pain is a message shaped at every level of the nervous system.

When the Alarm Gets Stuck On

After an injury, nerves and brain areas can stay alert even when tissues have healed. This state is called sensitisation. The alarm has become over-protective. It does not mean new damage; it means your system has learned to guard you too well.

The good news? What is learned can be unlearned.

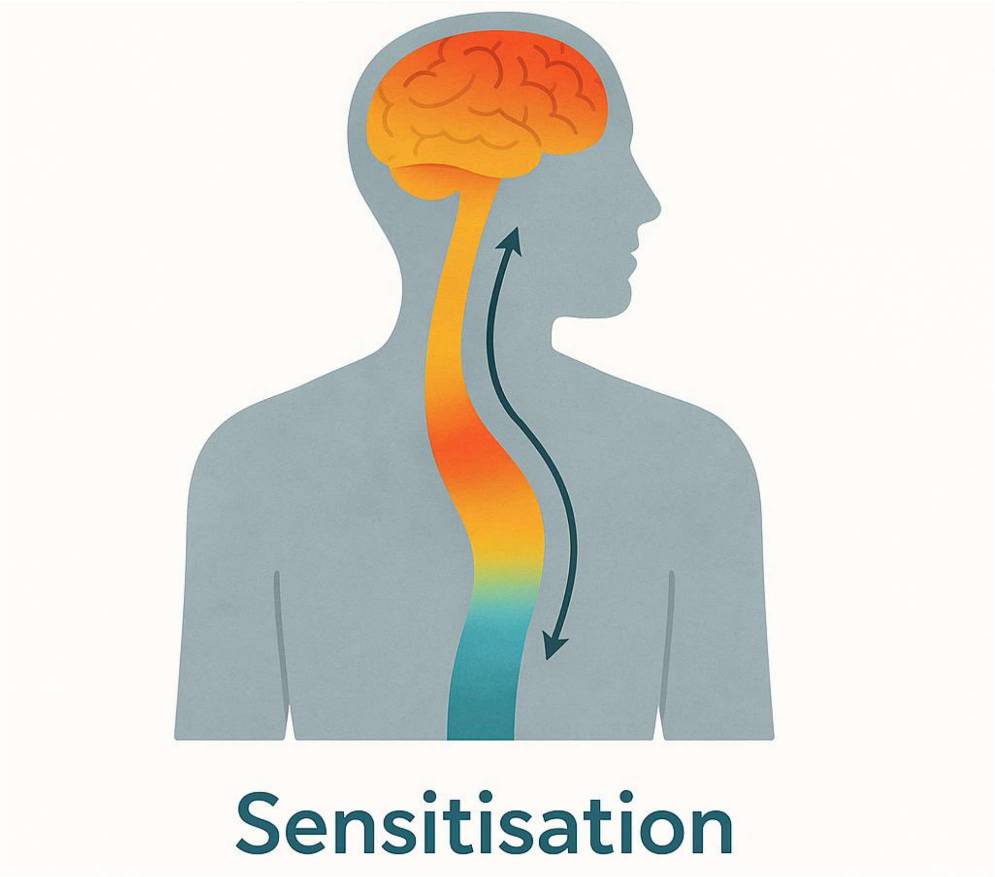

Sensitivity can rise, but the system can be retrained.

How Understanding Helps

- Knowing pain ≠ damage helps you move with confidence.
- Understanding itself lowers nervous-system threat levels.
- Each time you learn something new about pain, your brain updates its safety file.
- You start to feel less afraid and that is the first step toward healing.

Try This Today

1. Notice when pain rises: ask yourself, “Am I in danger, or just sensitive?”
2. Take three slow breaths and remind yourself, “My body is strong and learning to calm.”
3. Walk for two minutes, gently, as a signal of safety.
4. Write one reassuring sentence about your back and read it aloud tonight.

Talk with Your Clinician

- Ask them to explain what sensitivity means in your case.
- Discuss safe movements or activities you can restart this week.

Summary Message

Pain is like an alarm. Real, but sometimes too loud. Your body is strong, your system is protective, and understanding pain is the first medicine.

Cluster 2 – The Mind–Body Connection

Thoughts, Feelings, and Pain Work Together

The Over-Protective Guard

Imagine a guard outside your home. He means well. He wants to protect you. But lately, he's been jumpy. Even the sound of a cat or a passing truck makes him ring the alarm. The guard isn't bad, he's just too alert.

That's what happens in persistent pain. Your nervous system, the body's guard, becomes over-protective when it thinks you're in danger, even if you're not.

Your thoughts, fears, and emotions can either calm or excite that guard.

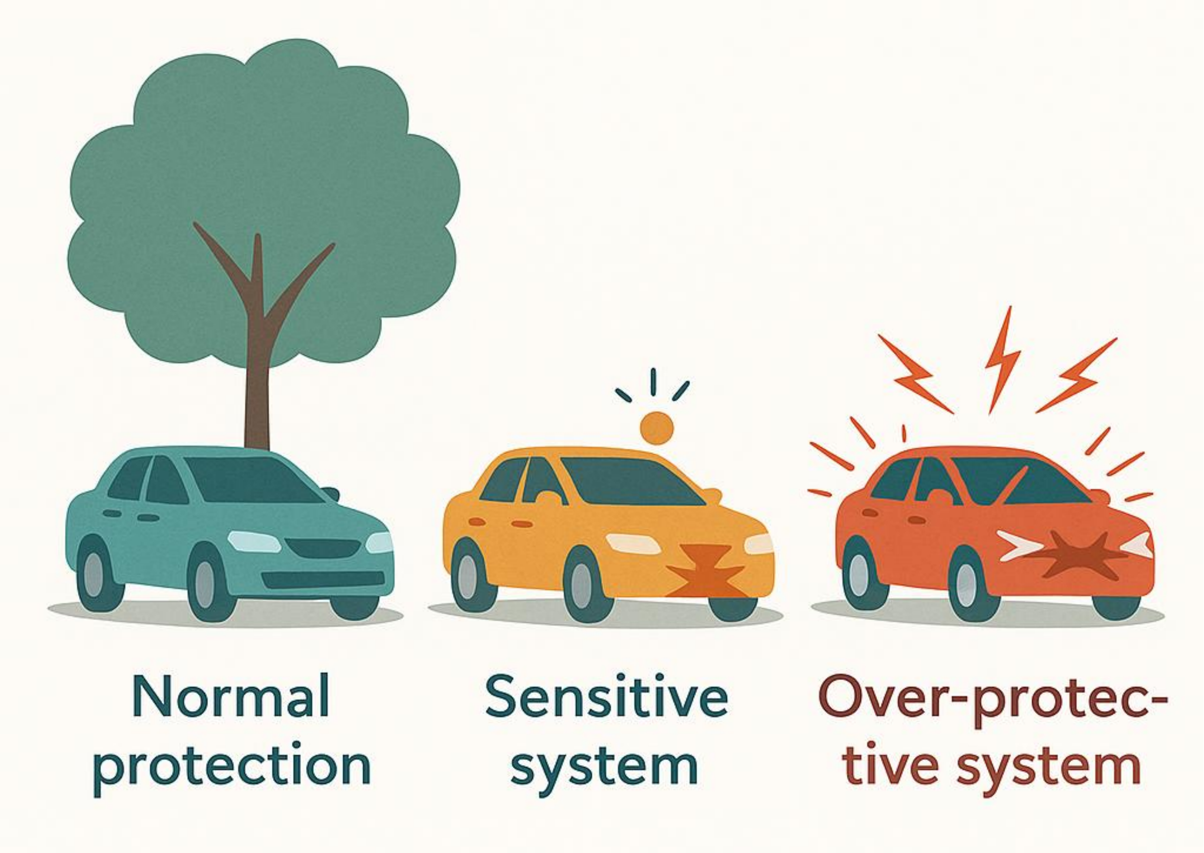

An over-protective alarm sounds even when there's no danger.

How the Mind and Body Talk to Each Other

- The body sends signals to the brain.
- The brain decides if those signals mean danger or safety.
- If the brain expects harm, pain increases.
- If it feels confident and safe, pain decreases.

Emotions like fear, anger, and worry can turn up the volume.
Hope, calm, and reassurance can turn it down.

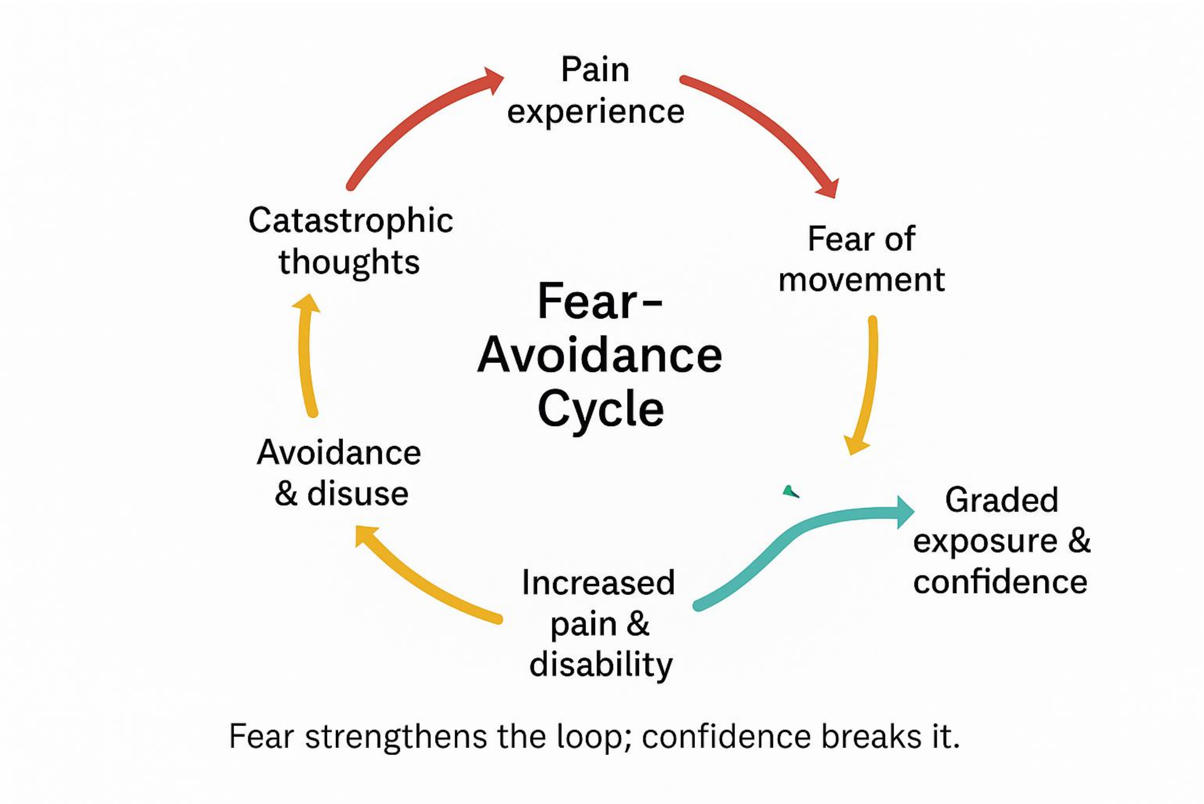

Breaking the Fear–Pain Cycle

When we fear pain, we often move less. When we move less, the body becomes stiff, weak, and more sensitive. This is called the fear-avoidance cycle.

The only way out is gentle, graded movement, proving to your nervous system that you are safe.

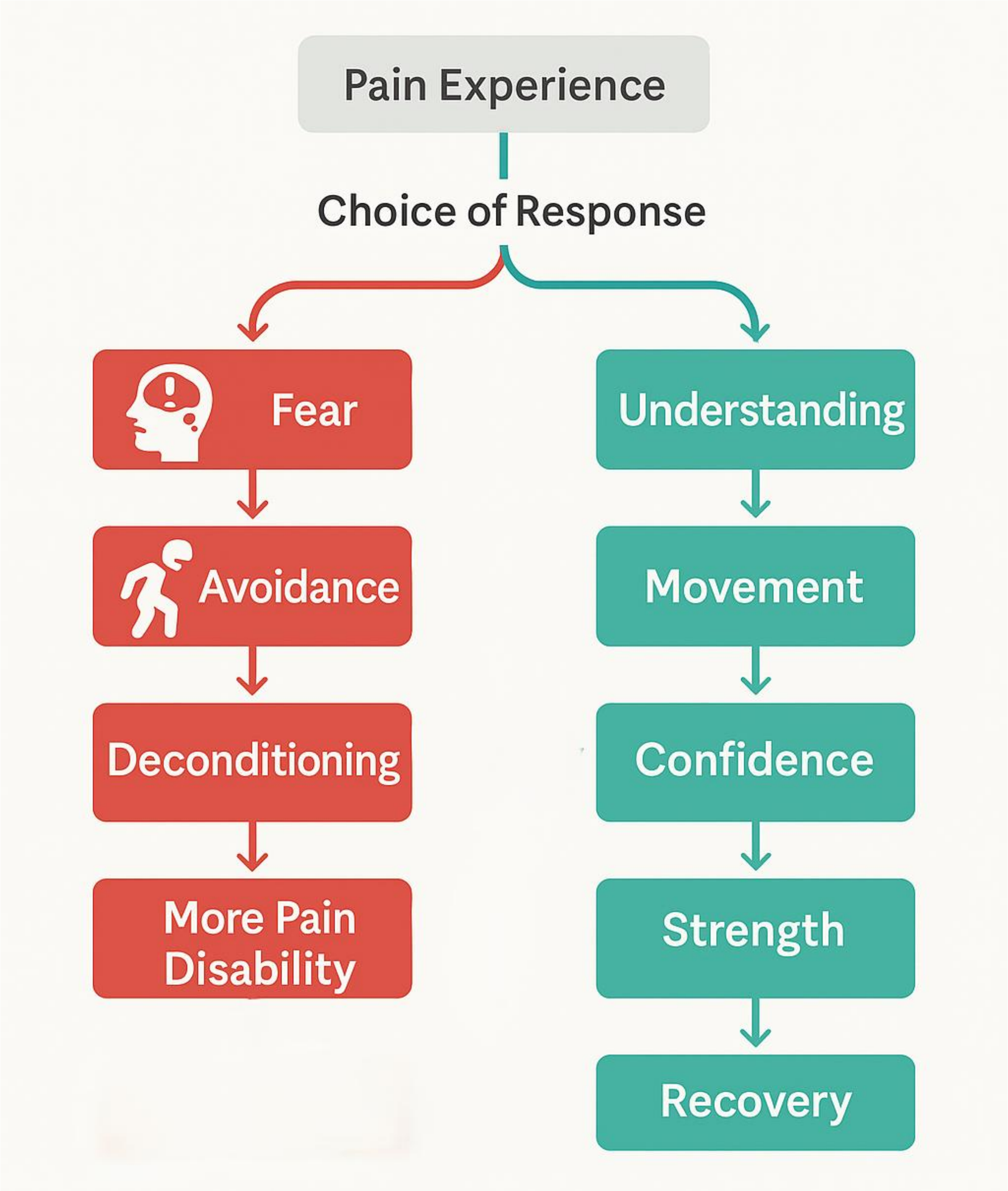

Two paths diverge: fear fuels pain, confidence can rebuild.

What Helps the Guard Calm Down

- Knowledge: Understanding that pain ≠ damage.
- Movement: Gentle motion tells the guard, “All is well.”
- Breathing: Slow, deep breaths reduce the alarm signals.
- Support: Encouraging words from family or your clinician lower the threat.

Even a small improvement in mood, hope, or confidence helps your brain quiet the alarm.

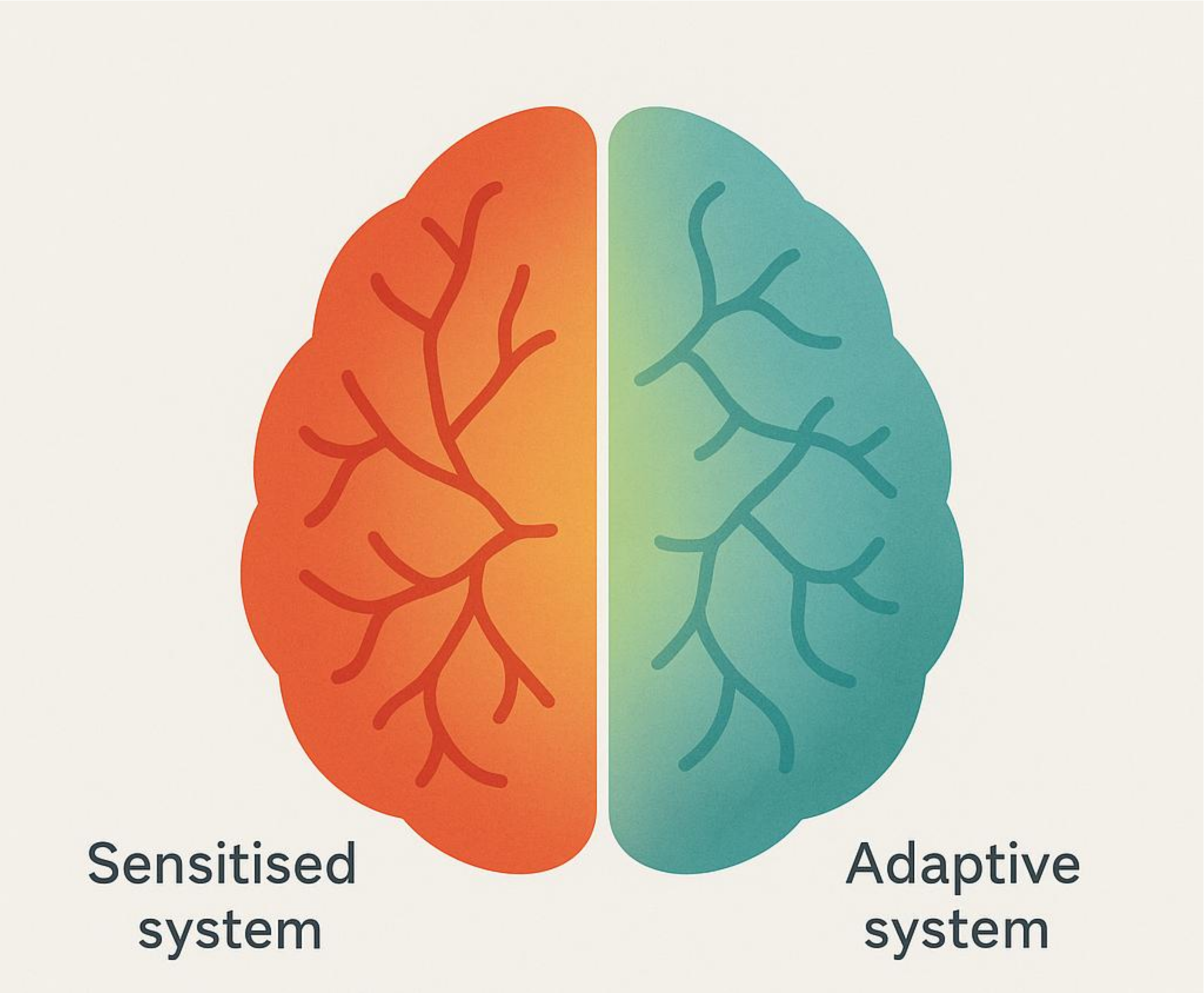

Learning and calm thoughts rewire the system toward safety.

Try This Today

1. When you feel pain, whisper to yourself: “I am safe; my body is learning.”
2. Move one small body part you’ve been avoiding. Stretch, turn, or bend gently.
3. Do three slow breaths through your nose, focusing on relaxation.
4. Write one fear you have about movement, then one fact that challenges it.

Talk With Your Clinician

- Ask: “Which activities are safe for me to restart?”
- Share any fears that stop you from moving or working.
- Request to learn about graded exposure, how to retrain confidence step-by-step.

Summary Message

Pain lives in both the body and the mind. Fear makes pain louder; confidence turns it down. You can retrain your system to feel safe again.

Cluster 3 – Motion Is Medicine

Every Step Teaches Safety

The Rusting Door Hinge

A door in your home that hasn't moved for months becomes rusty. When you finally push it open, it squeaks, not because it's broken, but because it hasn't moved. Once you open and close it gently a few times, it moves freely again.

Your back works the same way. When you stop moving from fear or pain, your body becomes stiff and sensitive.

Gentle movement "oils the hinges" and reminds your system that it is strong and safe.

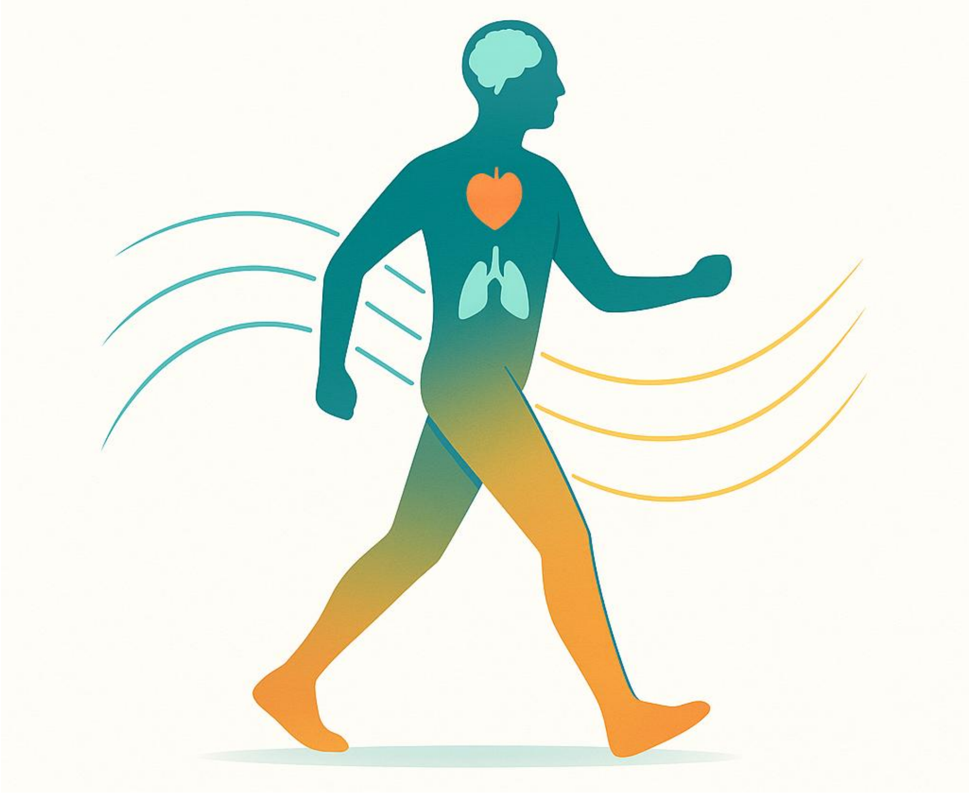

Movement restores calm and confidence.

Why Movement Is Medicine

- Movement sends healthy signals to the brain: “This part of the body is safe.”
- Blood flow increases, muscles relax, and calming chemicals (endorphins) are released.
- The brain learns again that bending, sitting, praying, or walking are safe actions.

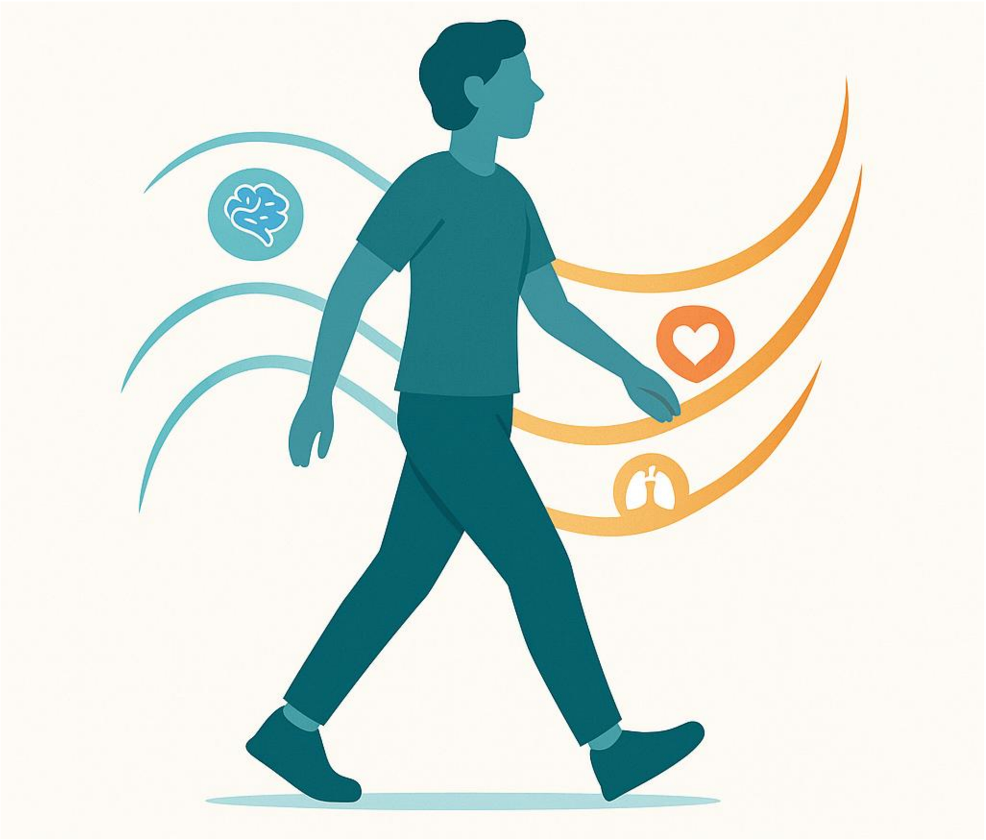

Small movements build big recovery.

Example

Some people say: I'll get better by resting. But long rest can make the system weaker and more fearful. Just as our daily prayers include movement, your body also needs rhythm and routine.

Gentle, regular movement is part of both ibadat (care of the body) and shukr (gratitude for function).

How to Move Safely

1. Start small: Walk inside your home for 5 minutes daily.
2. Pace yourself: Take breaks before pain spikes, not after.
3. Breathe and move: Pair slow breathing with gentle motion.
4. Increase gradually: Add a minute or a small stretch each day.
5. Track progress: Write one small success each evening (e.g., "I sat for 10 minutes pain-free.")

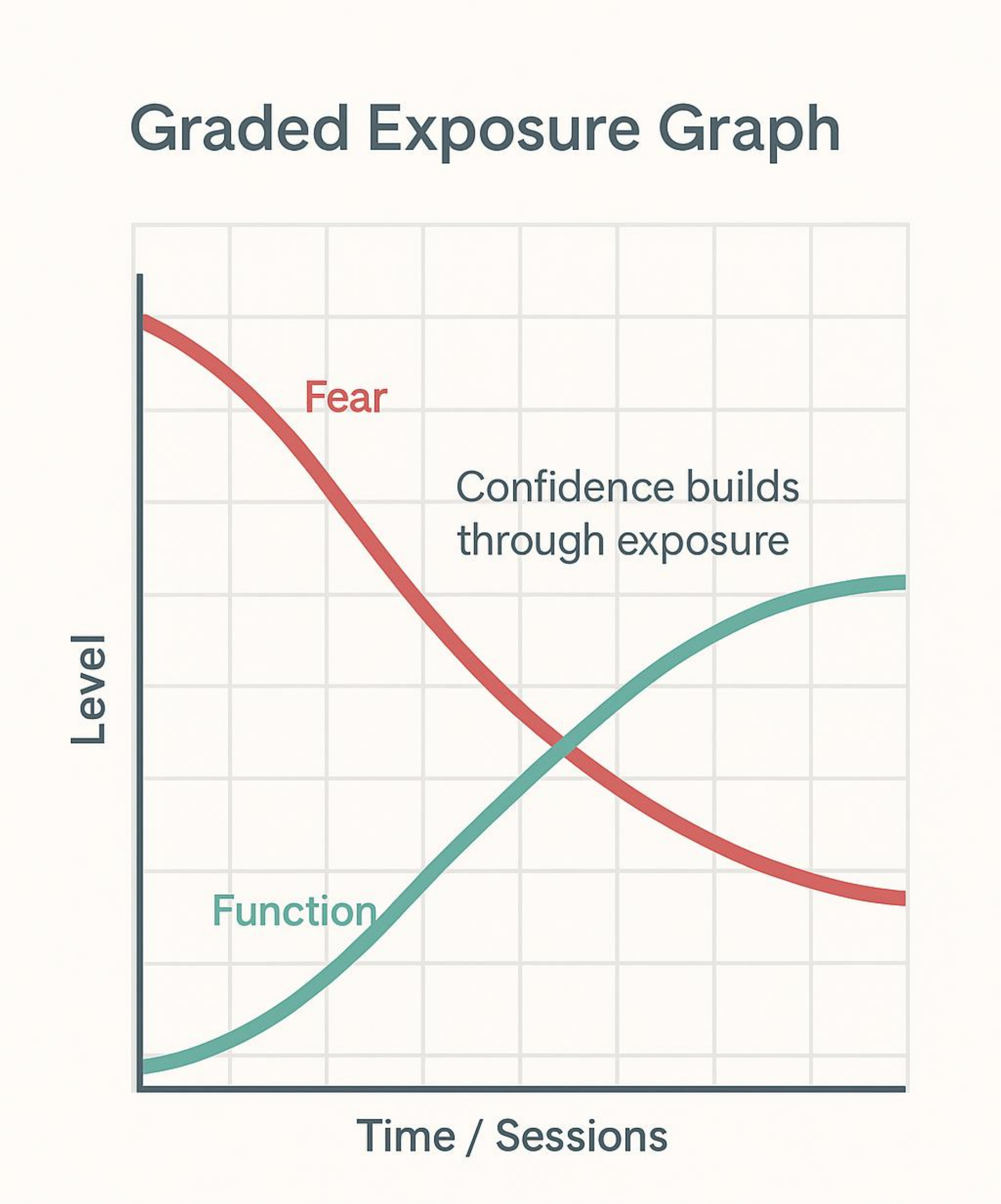

Confidence grows through repeated safe exposure.

Faith and Movement

The Prophet ﷺ encouraged moderation and strength, saying:

“A strong believer is better and more beloved to Allah than a weak one.” (Sahih Muslim)

Movement is not defiance of pain. It is trust in the body that Allah designed to heal.

Try This Today

- After every prayer, take 2 minutes to walk slowly and stretch.
- When pain appears, remind yourself: “This is my system learning safety.”
- Replace “I can’t” with “I’ll start gently.”
- Share your movement goals with a friend or family member for motivation.

Talk With Your Clinician

- Ask them to help design a safe movement or walking plan.
- Tell them what movements you fear most; they can show safe ways to re-introduce them.
- Discuss pacing: how to avoid the “boom and bust” cycle.

Summary Message

Movement is healing. Each gentle step quiets the alarm and rebuilds confidence. Your body is designed to move.

Cluster 4 – Healthy Habits & Support

Small Daily Choices Can Calm the Alarm

The Busy Home

In every household, mornings are full of movement. Breakfast cooking, school rush, office preparation. But when one person in the home is unwell, everyone changes their routine.

Sometimes family members say:

"Don't move too much, you'll make it worse."
They mean well, but too much protection can stop recovery. Healing happens best when everyone around you believes you can get stronger again.

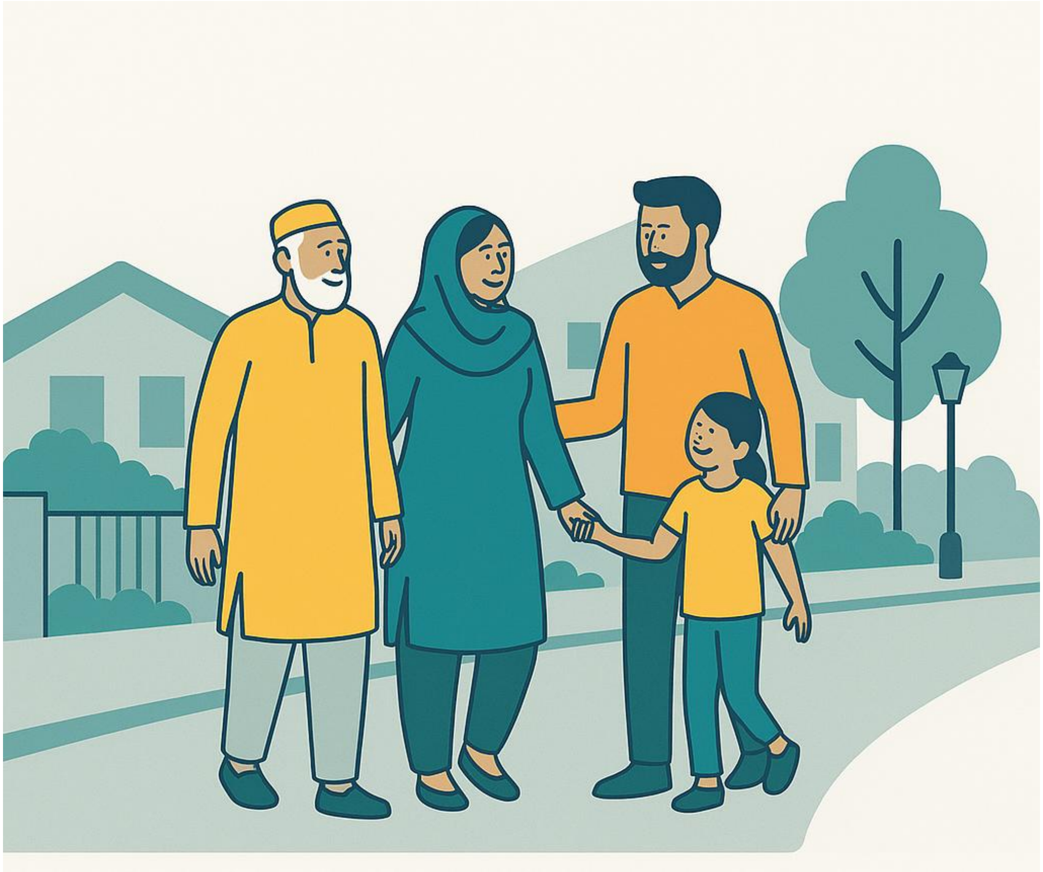

Community helps recovery.

Sleep and Pain

Good sleep can help in recovery. When sleep is poor, pain may feel sharper, energy drops, and the mind becomes fearful. Fixing sleep may help in reducing pain sensitivity.

Try:

- Go to bed at the same time every night.
- Avoid heavy food, caffeine, and phone use before bed.
- Take 3 deep breaths before lying down; say “Alhamdulillah” and consciously relax your muscles.

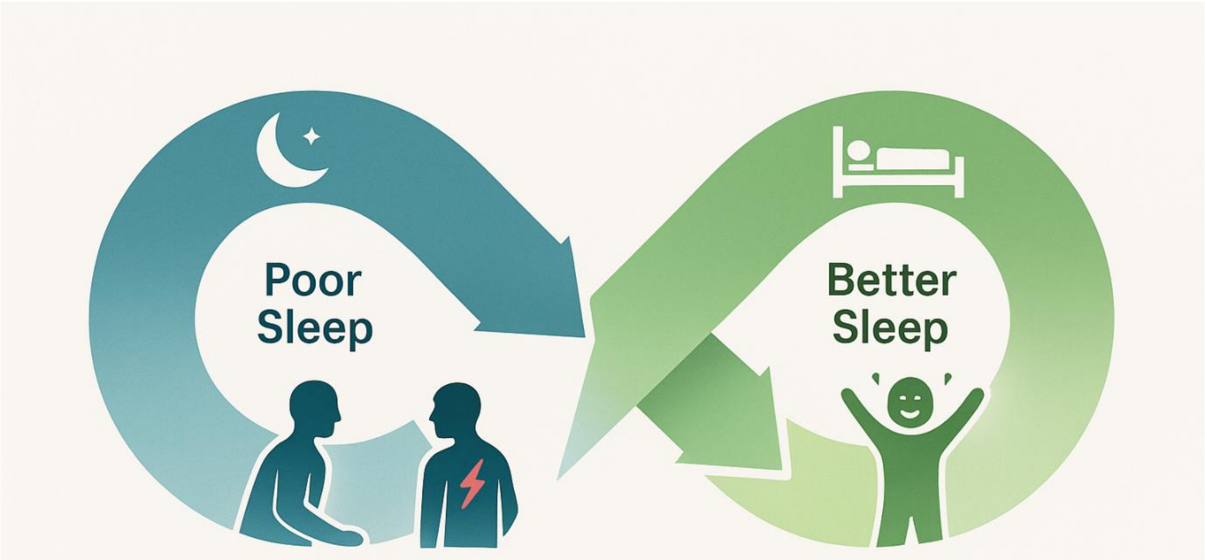

Breaking the cycle restores balance.

Stress and the Nervous System

Stress hormones keep your alarm system active. Financial pressure, family arguments, or worry about pain can all raise your sensitivity. But calm routines such as prayer, deep breathing, spending time outdoors help switch on your body's "rest and repair" system.

Reducing tension is part of treatment, not weakness.

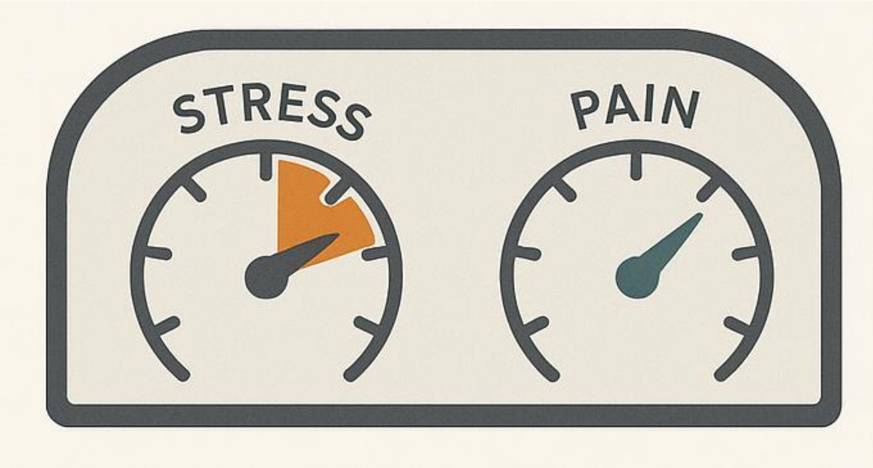

Your pain dashboard can be tuned through daily choices.

Food and Healing

What you eat influences your body's chemistry. Fruits, vegetables, nuts, fish, and whole grains lower inflammation. Too much sugar, oil, or processed foods increase it. You don't need a special western diet. Just balanced meals in moderation.

Example:

- Replace paratha oil with olive or canola oil.
- Add daal, sabzi, or fruit daily.
- Drink enough water. Dehydration makes muscles tight.
- Avoid skipping meals during stress or long work hours.

Words as Medicine

The words people use around you can heal or harm. Hearing “Your back is finished” increases fear and pain. Hearing “Your body is strong and healing” gives courage.

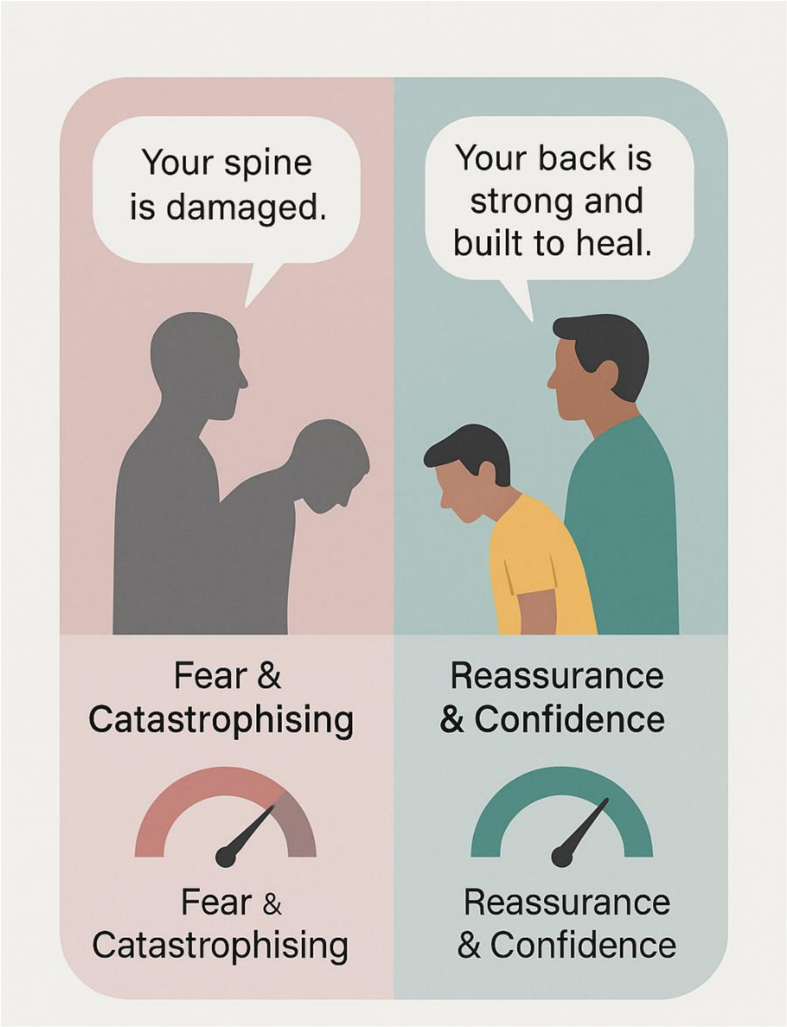

Language can help in healing.

Encourage family to say,

“You are improving” instead of “This pain will never go away”.

Family and Community Support

Our families are close and that is a blessing. When family members understand pain correctly, they become partners in recovery.

- Ask them to walk with you, not just care for you.
- Tell them what words encourage you.
- Invite them to your exercise or relaxation time.

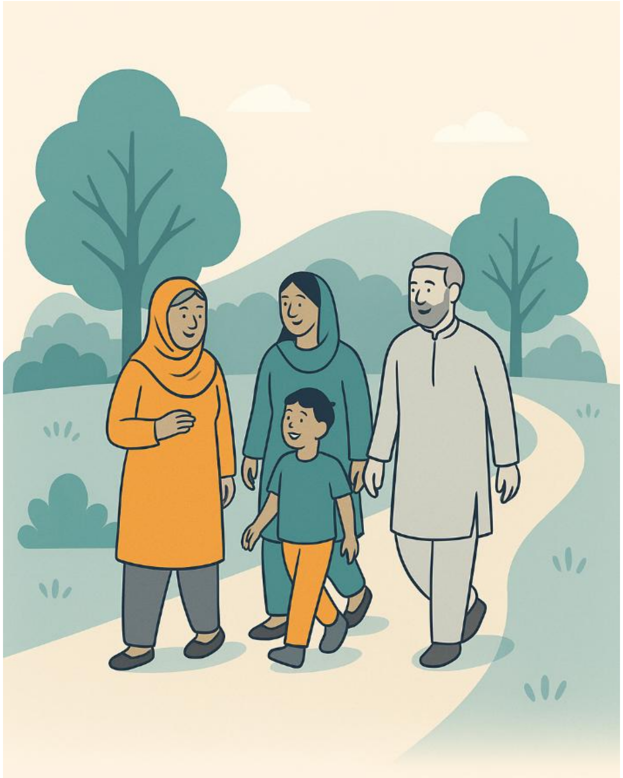

Healing grows through connection.

Try This Today

1. Walk with a family member after dinner, even for 5 minutes.
2. Replace one negative sentence with a hopeful one.
3. Eat one meal rich in colour (fresh vegetables, daal, or fruit).
4. Pray, breathe, or meditate for 2 quiet minutes during the day.
5. Switch off your phone 30 minutes before sleep.

Talk With Your Clinician

- Tell them about your sleep, diet, or stress challenges. They matter as much as medicine.
- Ask for advice about safe, moderate exercise during busy days or fasting periods.
- Discuss how family can support your pacing and activity goals.

Summary Message

Healing is a family and community journey. Sleep, stress, words, food, and faith all tune the alarm system. When your home, heart, and habits work together, pain becomes quieter.

Cluster 5 – Recovery and Hope

You Can Retrain Your System

The Sunrise After the Storm

After a long night of rain, the sun finally appears. The walls are damp, but the house still stands. You open the windows, let the air in, and the house begins to dry.

Your pain journey is the same. Storms come, fear, stiffness, stress, but they pass. The body and brain can learn calm again, just like the sky learns to clear.

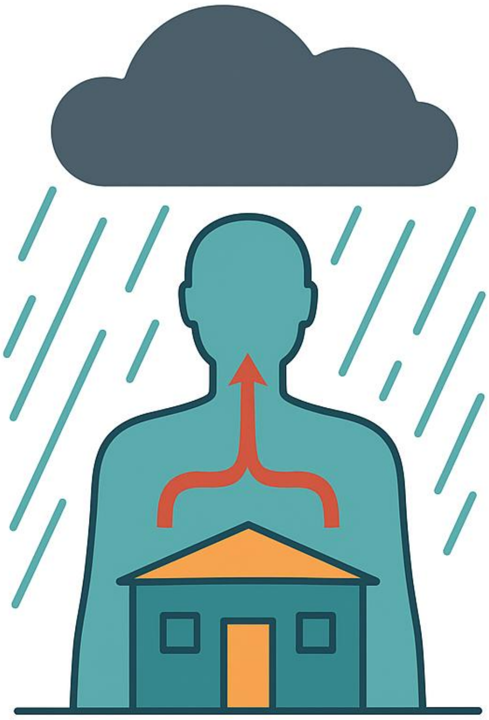

Pain may pour like rain, but the structure remains strong.

The Three Phases of Recovery

Recovery is not magic, it's training. You can retrain your nervous system through small, consistent actions. Think of recovery as three steps that build on each other:

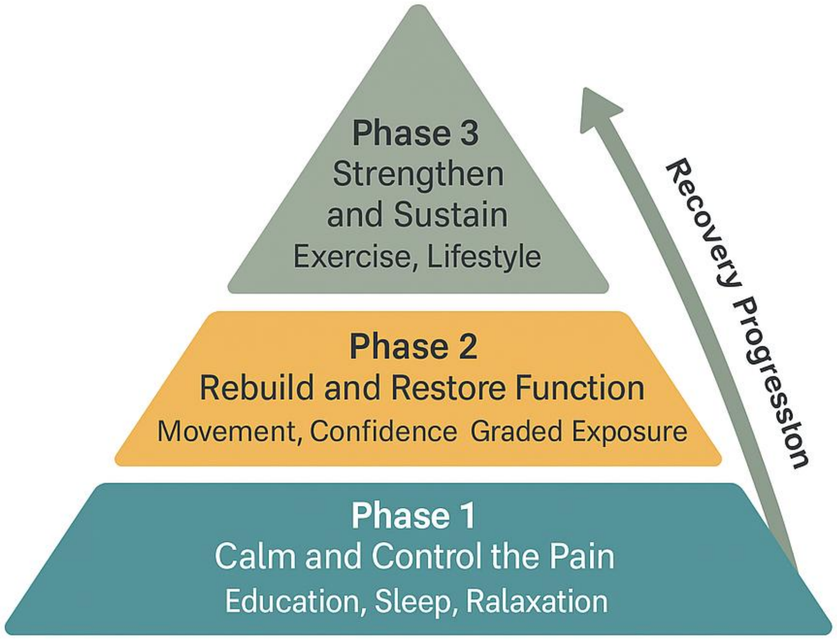

Progress from safety to strength.

1. **Calm and Control the Pain**
   - Learn about pain; breathe and rest wisely.
   - Improve sleep; move gently.
   - Tell yourself: "I am safe."
2. **Rebuild and Restore Function**
   - Start walking or stretching regularly.
   - Challenge fear through small, safe activities.
   - Believe: "My body is learning."
3. **Strengthen and Sustain**
   - Exercise, eat well, connect socially.
   - Balance activity with rest.
   - Think: *"I can handle this."*

Shifting from Passive to Active Recovery

Many people wait for someone else to “fix” them. But real recovery happens when you *participate in your own healing*.

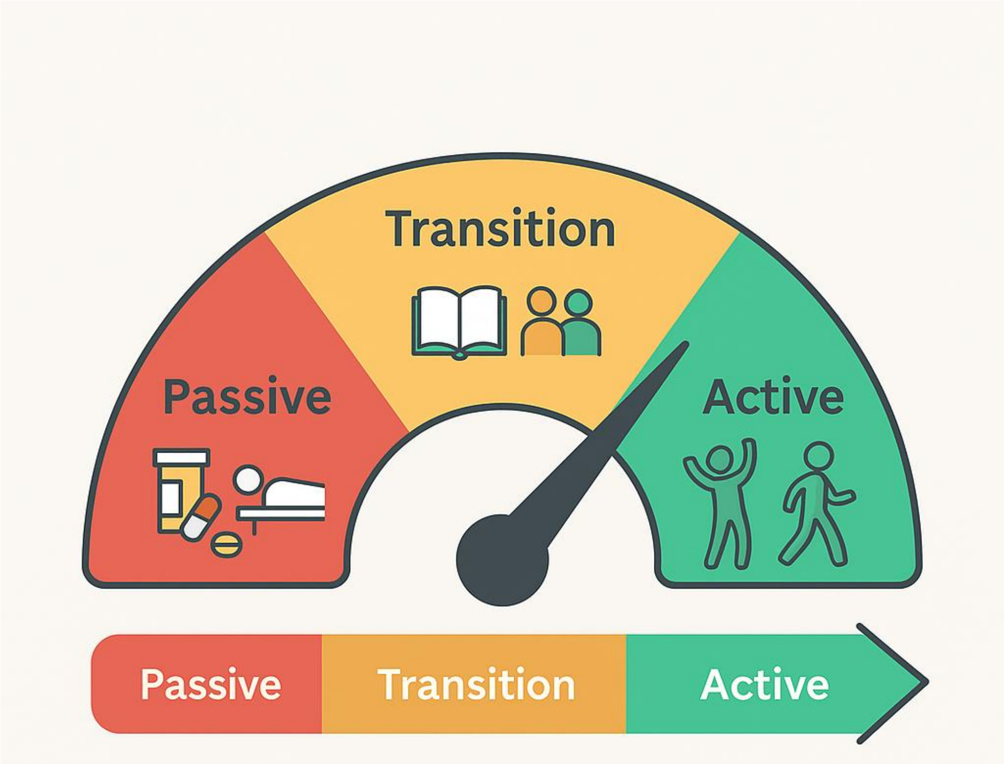

Shift from dependence to participation.

- Passive care (pills, injections, bed rest) can give short-term relief.
- Active care (education, movement, pacing) brings lasting change.
- When you become your own helper, your brain changes too.

How the Brain Heals

Pain lives in the nervous system, not only in the muscles or bones. When you move confidently, think positively, and learn about pain, the brain rewires its own pathways, a process called neuroplasticity.

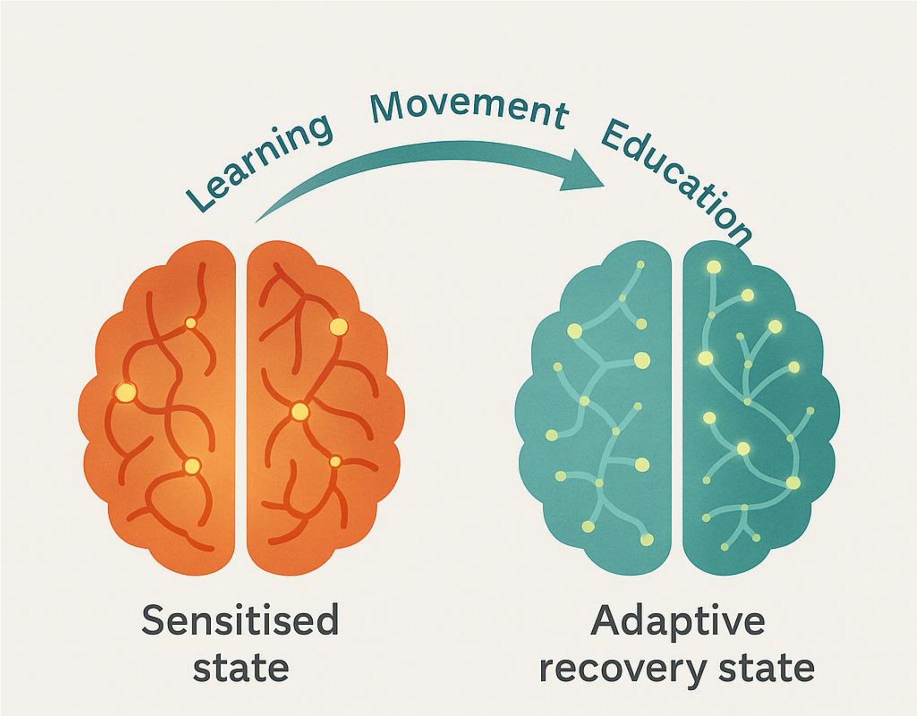

The brain can change and adapt.

Optimism strengthens calm circuits. Every positive thought, stretch, walk, or prayer builds new safe connections in your brain.

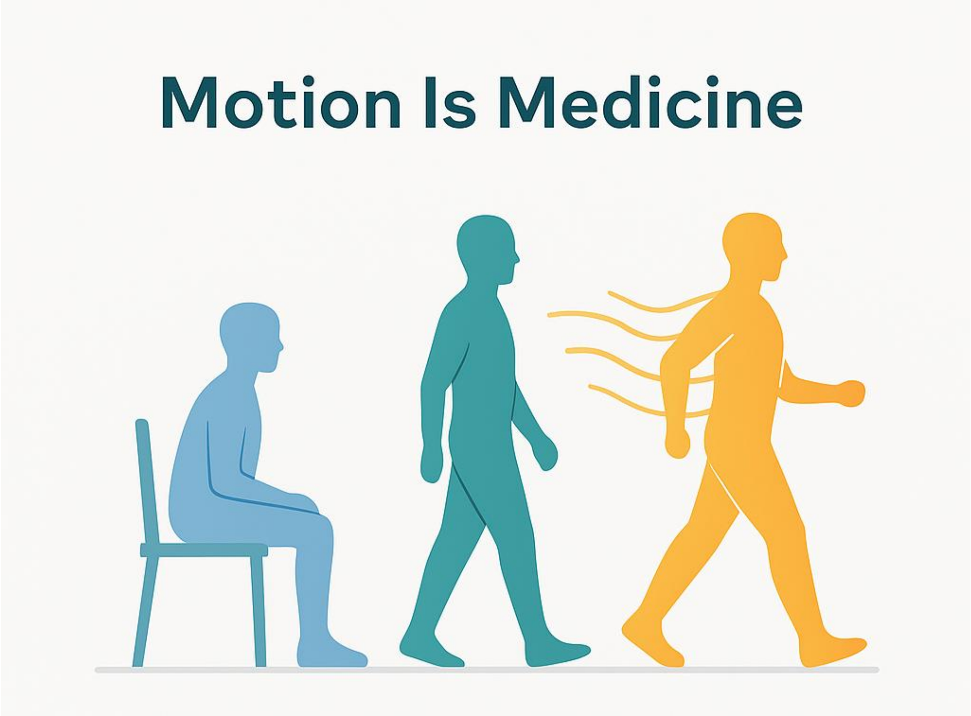

Positive expectation activates healing circuits.

Try This Today

1. Walk for five minutes longer than yesterday.
2. Replace the phrase “I’m broken” with “I’m rebuilding.”
3. Each morning, thank Allah for one part of your body that works well.
4. Do one kind thing for another person, it strengthens your healing too.
5. Before bed, imagine your body’s alarm becoming calm and quiet.

Talk With Your Clinician

- Share your daily goals and ask for feedback on pacing and exercise.
- Ask about long-term strategies to prevent flare-ups.
- Discuss any fears of relapse; make a simple plan for tough days.

Summary Message

Recovery is possible. Not by waiting, but by participating. Pain can change because the brain can change. Faith, knowledge, and movement together build healing. Every sunrise begins in darkness and every recovery begins with hope.
