# Supplementary material for: Development, Cultural Adaptation, and Content Validation of Urdu Pain Neuroscience Education Materials for Low Back Pain in Pakistan
Source: Med Sci (Basel). 2026 Jan 22;14(1):54. doi: 10.3390/medsci14010054 (PMC12921984; doi:10.3390/medsci14010054)
Supplement: Supplementary file 1 [file medsci-14-00054-s001.zip › S3 - Clinician Guide PNE Pakistan.pdf]

Section 1 – Introduction for Clinicians

"Educating pain is treating pain." – Moseley & Butler (2017)

Why Pain Neuroscience Education Matters

Low back pain (LBP) is not only a mechanical or structural condition. It is an experience shaped by biology, psychology, and social context.
In Pakistan, as in many low- and middle-income countries (LMICs), pain management still largely follows a biomedical model: medication, imaging, and rest. While these interventions have their place, evidence consistently shows that education, reassurance, and movement form the foundation of sustainable recovery (O'Sullivan et al., 2018; Louw et al., 2016).

Pain Neuroscience Education (PNE) helps patients understand that pain does not always mean damage, and that the nervous system can be retrained through learning, movement, and lifestyle change.

When taught effectively, PNE reduces fear, increases confidence, and encourages active participation - even in settings with limited resources.

The Challenge in the Pakistani Context

In Pakistan, pain beliefs are often shaped by:

- Cultural norms of protection and rest ("درد میں آرام ہی علاج ہے۔" - rest is the cure).
- Fear-based medical communication (patients being told "your disc is slipped" "آپ کی ڈسک سلپ ہو گئی ہے۔" or "Your back is damaged" "آپ کی کمر کو نقصان پہنچا ہے۔").
- Family overprotection: loved ones restricting the patient from moving.
- Religious interpretations of suffering: pain viewed as punishment rather than a physiological signal.
- Limited access to exercise-based rehabilitation in rural or low-income communities.

These beliefs, while compassionate in intent, often reinforce fear avoidance and dependence on passive care (e.g., injections, heat, electrotherapy).

By contrast, biopsychosocial pain education reframes pain as a protective but adaptable process, encouraging movement, hope, and autonomy.

The Role of Clinicians in Delivering PNE

Clinicians are not just providers of treatment; they are translators of science into understanding.
Each conversation is an opportunity to reshape beliefs, calm fears, and empower patients.
The way we explain pain can either:

- Activate the threat system ("Your spine is damaged" "آپ کی کمر کو نقصان پہنچا ہے۔"), or
- Activate the safety system ("Your back is strong and built to heal" " آپ کی پیٹھ مضبوط ہے اور خود کو ٹھیک کر سکتی ہے۔").

Evidence shows that brief educational encounters, even as short as 5–10 minutes, can significantly improve outcomes when clinicians use consistent, reassuring language (Louw et al., 2016; Traeger et al., 2019).

Culturally Sensitive PNE Delivery

Effective communication in Pakistan requires respect, empathy, and cultural fluency.
Clinicians should:

1. Use relatable metaphors, such as the House in Rain (body remains strong despite pain) or the Over-Protective Guard (nervous system sensitivity).
2. Incorporate faith-aligned language, e.g., "The body is an امانت (trust) that we must care for."
3. Engage family members, they are often decision-makers in care and can either hinder or facilitate recovery.
4. Respect modesty and gender norms during demonstrations or movement sessions.
5. Highlight the balance between صبر (patience) and عمل (action), linking effort to faith, not defiance of pain.

Aims of This Clinician Guide

This guide equips physiotherapists, exercise professionals, and physicians with:

- Simplified, evidence-based scripts for explaining key PNE concepts.
- Cultural and faith-based communication strategies suited to Pakistani patients.
- Practical visual tools to support education sessions.
- Five patient-centred modules that align with the PNE Patient Booklet for consistent messaging.

Key Learning Outcomes for Clinicians

After completing this training guide, clinicians will be able to:

1. Explain pain in clear, non-threatening language.
2. Identify and address unhelpful beliefs about pain.
3. Use visuals and metaphors to improve patient understanding.
4. Encourage active, self-managed recovery.
5. Deliver culturally sensitive education across clinical and community settings.

Suggested Readings

- Louw A, Zimney K, Puentedura EJ, Diener I. (2016). The clinical application of teaching people about pain: A systematic review. Physiotherapy Theory and Practice, 32(5), 332–355.
- O'Sullivan PB, Caneiro JP, O'Sullivan K, et al. (2018). Back to basics: 10 facts every person should know about back pain. British Journal of Sports Medicine, 52(24), 1547–1548.
- Traeger AC, Lee H, Hubscher M, et al. (2019). Effect of intensive pain education and physiotherapy on chronic low back pain. JAMA, 321(4), 292–301.
- Moseley GL & Butler DS. (2017). Explain Pain Supercharged. Noigroup Publications.

Summary Message for Clinicians

"Every sentence you speak about pain can either fuel fear or build freedom.
Educate, reassure, and empower because understanding is treatment."

Section 2 – Cluster 1: Understanding Pain

Theme for Patients: "Pain is protection, not damage."

Theme for Clinicians: "Explain the alarm before you treat the pain."

Clinical Learning Objectives

By the end of this session, clinicians will be able to:

1. Explain pain as a protective output of the nervous system, not a direct marker of tissue damage.
2. Deliver this concept using simple, culturally meaningful metaphors.
3. Reduce fear by shifting language from structural to functional explanations.
4. Engage patients and families in understanding that learning itself is part of healing.

Key Concept Summary

Pain arises when the brain perceives threat, not necessarily when damage exists.
After an injury, nerves and brain networks can become over-protective - sending pain signals even when tissues are healed.
This is known as sensitisation, and it is reversible through education, movement, and reassurance.

Patients often say: "But I still feel pain, so something must be wrong."

Our role is to reply: "Pain means your system is alert, not broken. It's trying to protect you, but we can help it calm down."

Teaching Metaphor: The House in the Rain

"Your body is like a house during a monsoon."
The rain (pain) pours heavily, but the structure (your body) is strong. The alarm rings loudly because the system wants to protect you - not because the walls are falling."

This metaphor works powerfully in the Pakistani context because:

- Rain and flooding are familiar experiences.
- It shifts focus from "broken parts" to "protective systems."
- It naturally invites discussion around safety, maintenance, and reassurance.

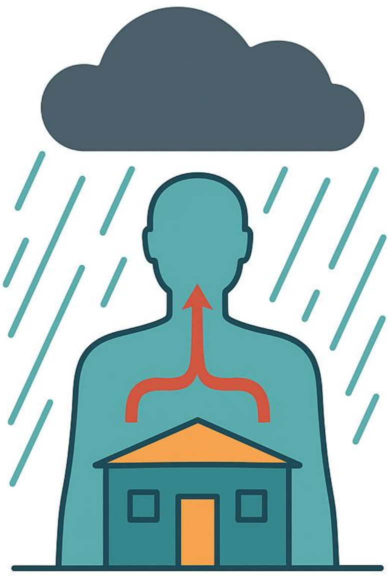

Pain may pour like rain, but the structure remains strong.

Clinician Tip: Use your hand as the “roof” to demonstrate how alarms protect, not destroy.

<u>Key Phrases to Use</u>

**Helpful Language**	**Rationale**
“Pain is your body’s alarm system.”<br>”درد آپ کے جسم کا الارم سسٹم ہے۔“	Creates a safety framework.
“Your back is strong and built to heal.”<br>”آپ کی پیٹھ مضبوط ہے اور خود کو ٹھیک کر سکتی ہے۔“	Counters the cultural belief that pain equals damage.
“Sometimes the alarm becomes too sensitive, but we can retrain it.”<br>” بعض اوقات الارم بہت حساس ہو جاتا ہے، لیکن ہم اسے دوبارہ تربیت دے سکتے ہیں۔“	Introduces neuroplasticity without jargon.
“Pain is real, but it’s also changeable.”<br>”درد حقیقی ہے، لیکن یہ بدل سکتا ہے“	Validates patient experience.

Phrases to Avoid

**Unhelpful Language**	**Why It's Harmful**
"Your disc has slipped."<br>"آپ کی ڈسک سلپ ہو گئی ہے۔"	Creates fear and catastrophising.
"You have wear and tear."<br>"آپ کی کمر میں ٹوٹ پھوٹ ہے۔"	Reinforces ageing as damage.
"It's just psychological."<br>"یہ صرف نفسیاتی ہے۔"	Invalidates pain experience and increases stigma.
"You'll have to live with it."<br>"آپ کو اس کے ساتھ ہی رہنا پڑے گا۔"	Reduces hope and self-efficacy.

Mini Clinical Activity (5 minutes)

Objective: Help patients experience the concept of *pain ≠ damage.*

Instructions:

1. Ask the patient to recall a time when they felt pain without injury - e.g., touching a hot cup or having a headache.
2. Ask: "Did that pain mean you were injured?"
3. Explain: "That same alarm can sometimes keep ringing when the body is already safe."
4. Draw a quick sketch of an alarm system or use the House in Rain image.

Reflection Prompt for Clinicians:
"What every day analogies work best in your own language or region; rain, security alarms, or home repairs?"

Cultural Communication Tips

1. Respect faith and fatalism:
   If a patient says, "It's Allah's test," affirm it, then gently add: "Yes, and Allah also gives us knowledge to manage the test wisely" "جی ہاں، اور اللہ ہمیں علم بھی دیتا ہے کہ ہم امتحان کو سمجھداری سے سنبھال سکیں".
2. Engage the family:
   Turn to relatives and say: "Encouraging movement and calm helps the healing, rest

alone slows it down" " حرکت اور سکون کی حوصلہ افزائی سے شفا یابی میں مدد ملتی ہے، صرف آرام اسے سست کر دیتا ہے۔"

3. Use relatable activities:
   Replace "sports" examples with daily tasks; cooking, walking to mosque, lifting a grandchild.

4. Validate pain:
   Say, "Your pain is real, but your body is not broken." " آپ کا درد حقیقی ہے، لیکن آپ کا جسم ٹوٹا نہیں ہے۔"
   This bridges the gap between empathy and empowerment.

Supporting Visuals

- **Primary Visual:** *Figure: House in Monsoon Rain*
- **Secondary Visual:** *Figure: Pain Loop* (for more analytical patients)
- **Optional Clinician Sketch:** quick "brain-spine-body" loop drawn on a whiteboard.

Note: Show Pain Loop only after the metaphor, it reinforces rather than confuses.

Session Summary for Clinicians

**Concept**	**Clinician Takeaway**
Pain is an alarm, not a damage report.	Teach safety, not fear.
Patients need reassurance, not repair talk.	Use strength-based language.
Cultural metaphors work better than medical jargon.	Use rain, light, or home analogies.
Learning changes biology.	Education is treatment.

Suggested Script (Example Delivery)

"When we see pain lasting longer than expected, it doesn't always mean something is damaged. It's more like a house alarm that became over-sensitive, it rings even when the wind blows. Your spine is strong, but your system is alert.
The good news is that alarms can be reset, and that's what we'll do together through

education, gentle movement, and confidence.”
” جب ہم دیکھتے ہیں کہ درد توقع سے زیادہ دیر تک رہتا ہے، تو اس کا ہمیشہ یہ مطلب نہیں ہوتا کہ کچھ خراب ہو گیا ہے۔

یہ گھر کے الارم کی طرح ہے جو حد سے زیادہ حساس ہو گیا ہے، یہ اس وقت بھی بجتا ہے جب ہوا چلتی ہے۔

آپ کی ریڑھ کی ہڈی مضبوط ہے، لیکن آپ کا نظام الرٹ ہے۔

اچھی خبر یہ ہے کہ الارم کو دوبارہ ترتیب دیا جا سکتا ہے، اور یہی ہم تعلیم، نرم حرکت اور اعتماد کے ذریعے مل کر کریں گے۔”

End-of-Session Clinician Reflection

“What metaphors or phrases helped your patient’s face relax today?”
“Did your language invite hope or reinforce fear?”

Key Teaching Summary for Cluster 1

Learning Goal	Patient Message	Clinician Action
Explain that pain ≠ damage	“Pain protects you.”<br>“درد آپ کی حفاظت کرتا ہے۔”	Use *House in Rain* metaphor.
Introduce sensitisation	“Your alarm is too sensitive.”<br>“آپ کا الارم بہت حساس ہے۔”	Show *Pain Loop* diagram.
Rebuild confidence	“Your body is strong and learning to calm.”<br>“ آپ کا جسم مضبوط ہے اور پرسکون رہنا سیکھ رہا ہے۔”	Use strength-based reassurance.

Section 3 – Cluster 2: Mind–Body Connection

Theme for Patients: "Fear makes pain louder; calm turns it down."

Theme for Clinicians: "Change the story: fear feeds the pain, safety soothes it."

Clinical Learning Objectives

By the end of this session, clinicians will be able to:

1. Explain the fear-pain relationship using simple metaphors.
2. Recognise when patients display fear-avoidance behaviours.
3. Teach emotional regulation (calm, confidence) as a physical treatment.
4. Use faith- and family-sensitive communication to reduce fear and increase engagement.

Core Clinical Concept

Fear and anxiety amplify pain because they activate the body's protective alarm systems. When a patient feels threatened by movement, stress, or even medical explanations, the brain turns up the "pain volume." Conversely, when the person feels safe, supported, and confident, the nervous system quiets down.

"Pain is not in the head, but the head can help turn it down."

This is not psychology replacing biology, it's biology and psychology working together.

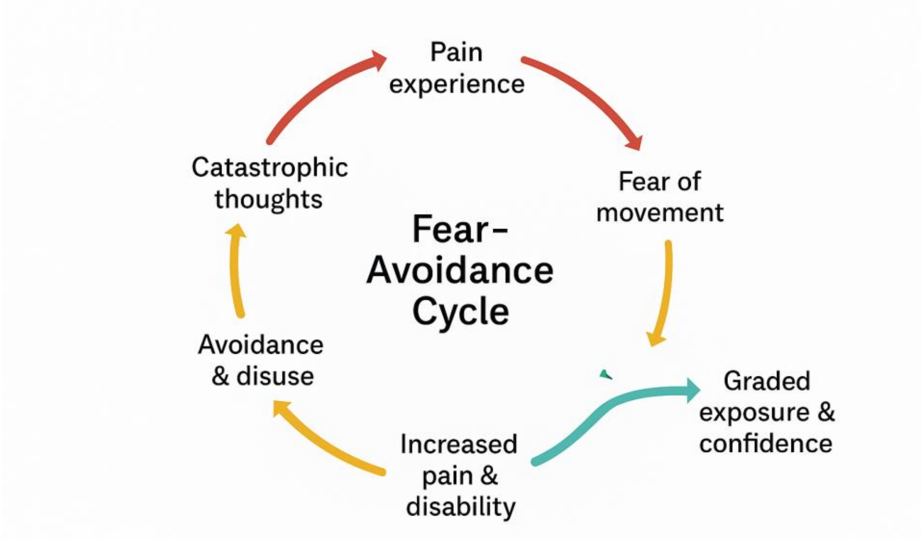

Fear strengthens the loop; confidence breaks it.

Clinician Tip: Draw the loop on paper: Fear → Avoidance → Weakness → More Pain → More Fear. Then draw an "exit arrow" labelled "Confidence and Movement."

Teaching Metaphor: The Over-Protective Guard

"Think of your nervous system as a guard outside your house. He's loyal, but jumpy. He used to respond only to real danger, but now he shouts even when a cat walks by."

This metaphor is relatable in Pakistan because:

- "چوکیدار" or night guard is a familiar cultural figure.
- It externalises pain, helps patients see it as protection, not damage.
- It invites empathy toward their own body rather than frustration.

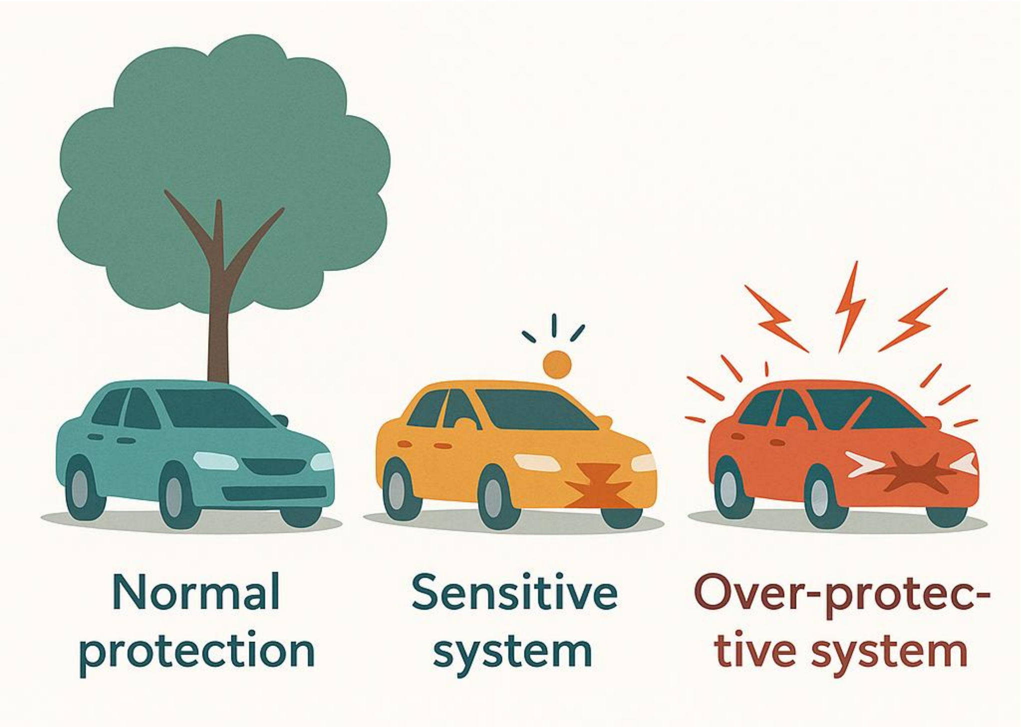

An over-protective system can be retrained through calm and confidence.

Key Phrases to Use

Helpful Language	Purpose
"Fear makes the alarm louder."<br>"خوف خطرے کی گھنٹی کو تیز کرتا ہے۔"	Links emotion directly to pain.
"Your body is learning safety again."<br>"آپ کا جسم دوبارہ حفاظت سیکھ رہا ہے۔"	Encourages hope and agency.
"You can't always control pain, but you can control what it learns."<br>"آپ ہمیشہ درد کو کنٹرول نہیں کر سکتے، لیکن آپ اس پر قابو پا سکتے ہیں کہ آپ کا جسم کیا سیکھتا ہے۔"	Strengthens self-efficacy.
"The brain protects you, it just needs better information."<br>"دماغ آپ کی حفاظت کرتا ہے، اسے صرف بہتر معلومات کی ضرورت ہے۔"	Removes blame and stigma.

Phrases to Avoid

Unhelpful Language	Why It's Harmful
"It's all psychological." "یہ سب نفسیاتی ہے۔"	Invalidates the experience.
"It's just in your head."<br>"یہ صرف آپ کے ذہن میں ہے۔"	Reinforces stigma about mental health.
"Ignore it and it will go away."<br>"اسے نظر انداز کریں اور یہ دور ہوجائے گا۔"	Oversimplifies and frustrates.
"You need to be stronger mentally."<br>"آپ کو ذہنی طور پر مضبوط ہونے کی ضرورت ہے۔"	Implies weakness instead of protection.

Mini Clinical Activity (5 minutes)

Objective: Help the patient recognise the fear–pain connection.

Instructions:

1. Ask: "What activities do you avoid because you're afraid of hurting yourself?"
2. Write them down together.
3. Draw a circle labelled Fear → Avoidance → Weakness → More Pain → Fear.
4. Say: "This is what your brain is doing, protecting you too much. Let's break this loop."
5. Circle the word Movement and say: "This is your way out."

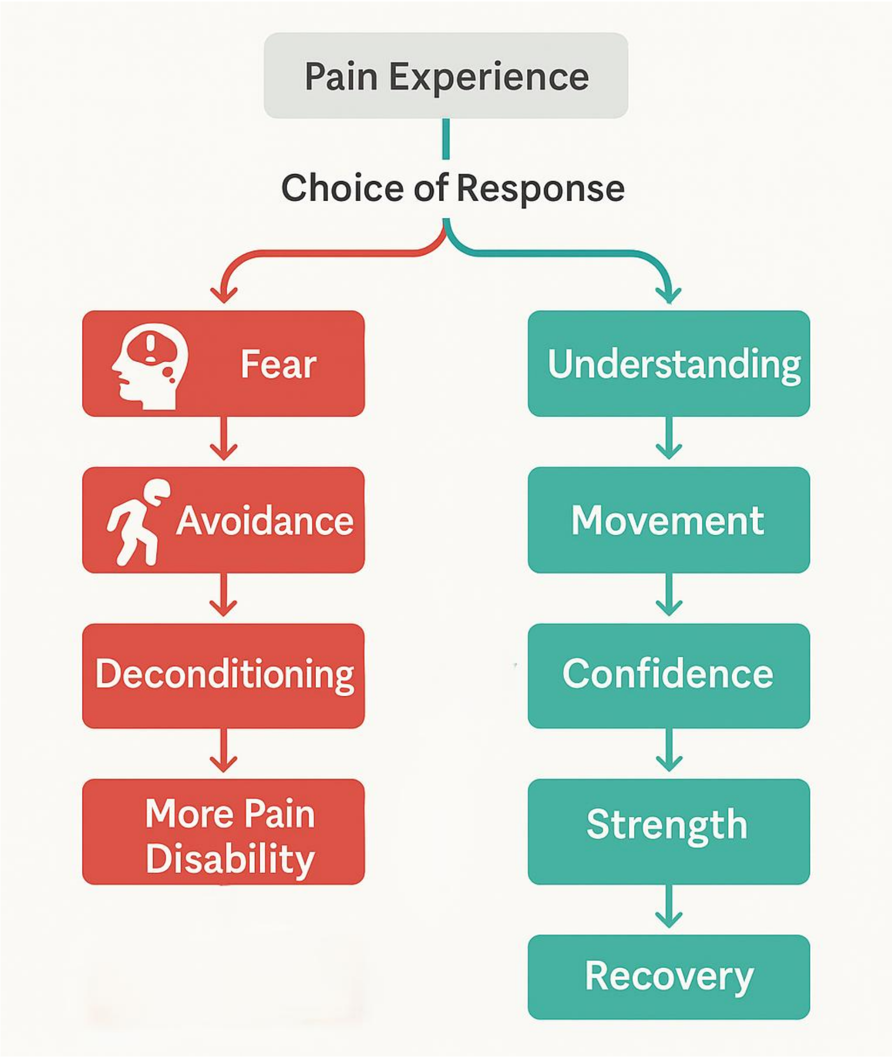

Confidence leads to recovery; fear keeps the loop going.

Faith and Cultural Integration

When patients express fear or hopelessness through religious phrases, e.g., "Maybe Allah wants me to suffer" *"شاید اللہ چاہتا ہے کہ میں تکلیف میں رہوں"* - clinicians should respectfully reframe:

"Allah also gives knowledge and courage to handle it. Moving wisely is not defiance, it's part of Shukr (gratitude) for the body's strength."

"اللہ اس کو سنبھالنے کا علم اور ہمت بھی دیتا ہے۔

عقلمندی سے حرکت کرنا نافرمانی نہیں، یہ جسم کی طاقت کے لیے شکر کا حصہ ہے"

Family involvement is critical:

- Encourage families to use positive, reassuring words (“You can do it” “آپ یہ کر سکتے ہیں۔”) instead of restrictions (“Don’t move or you’ll damage it more” “حرکت نہ کریں ورنہ آپ اسے مزید نقصان پہنچائیں گے۔”).
- When treating women, acknowledge modesty concerns, suggest private or home-based exercises.
- When treating elders, frame movement as regaining independence, not disobedience to advice.

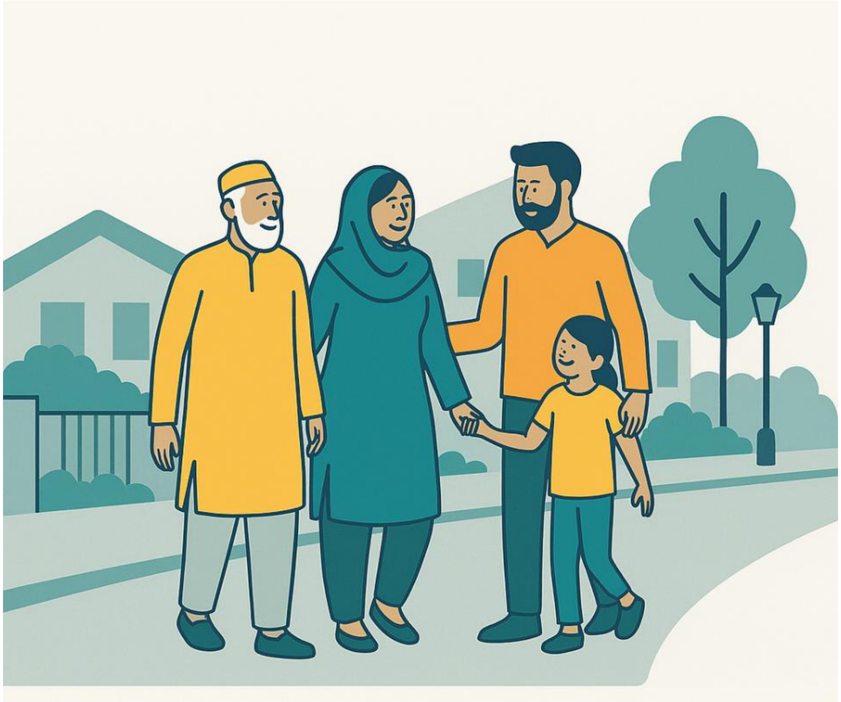

Recovery is stronger when families walk together.

<u>Clinician Demonstration: The Breath–Mind Reset</u>

Demonstrate the 3-3-3 Calm Reset technique:

- 3 slow breaths in through the nose,
- 3 seconds hold,
- 3 seconds exhale.
  Explain that breathing calms the *threat response* in the nervous system.
  Encourage patients to use this during flare-ups or anxious moments.

Sample Clinician Script

“Your body and brain are trying to protect you, not punish you.
When we feel scared, the brain tightens the alarm. But when we understand pain, breathe, and move, the brain learns that we are safe. Every calm breath and small movement is a lesson in safety.”

“آپ کا جسم اور دماغ آپ کی حفاظت کرنے کی کوشش کر رہے ہیں، آپ کو سزا دینے کی نہیں۔

جب ہم خوف محسوس کرتے ہیں تو دماغ خطرے کی گھنٹی بجاتا ہے۔ لیکن جب ہم درد کو سمجھتے ہیں، سانس لیتے ہیں اور حرکت کرتے ہیں تو دماغ کو معلوم ہوتا ہے کہ ہم محفوظ ہیں۔ ہر پرسکون سانس اور چھوٹی حرکت حفاظت کا سبق ہے۔”

Cultural Communication Tips

Situation	Suggested Clinical Response
Patient says: “I’m scared to bend; my disc will slip.” “ میں جھکنے سے ڈرتا ہوں؛ میری ڈسک پھسل جائے گی۔”	“I understand, many people think that. But the spine is strong and built to move. Let’s test it gently together.” “ میں سمجھتا ہوں، بہت سے لوگ ایسا سوچتے ہیں۔ لیکن ریڑھ کی ہڈی مضبوط ہے اور حرکت کرنے کے لیے بنائی گئی ہے۔ آئیے مل کر اسے آہستہ سے جانچیں۔”
Family says: “Doctor, don’t let her move much.” “ڈاکٹر، اسے زیادہ ہلنے نہ دیں۔”	“Actually, gentle movement helps healing. Resting too long can delay recovery.” “ درحقیقت، ہلکی حرکت سے شفا یابی میں مدد ملتی ہے۔ زیادہ دیر آرام کرنے سے صحت یابی میں تاخیر ہو سکتی ہے۔”

Supporting Visuals

- *Fear-Avoidance Cycle*
- *Two Paths: Fear vs Recovery*
- *Car Alarm / Over-Protective Guard*
- *Cultural Strengths*

Clinician Tip: Let the patient describe what the image reminds them of; their personal metaphor may be even stronger.

Session Summary for Clinicians

**Concept**	**Clinician Takeaway**
Pain sensitivity rises with fear.	Teach calm confidence, not toughness.
Emotions are biological.	Validate emotional experience as part of healing.
The nervous system learns from reassurance.	Use supportive tone and eye contact.
Family words matter.	Teach caregivers to “speak safety.”

End-of-Session Reflection

“What words did my patient hear today, fear or safety?”
“Did my explanation create calm, or confusion?”

Key Teaching Summary for Cluster 2

**Learning Goal**	**Patient Message**	**Clinician Action**
Explain fear–pain link	“Fear makes pain louder.”	Use the Over-Protective Guard metaphor.
Introduce graded confidence	“Small steps teach safety.”	Plan a safe movement the same day.
Engage family & faith	“Support brings calm.”	Include caregivers in education.

Section 4 – Cluster 3: Motion Is Medicine

Theme for Patients: "Every step teaches safety."

Theme for Clinicians: "Movement is the treatment, not the test."

Clinical Learning Objectives

By the end of this section, clinicians will be able to:

1. Explain why *movement is safe and essential* for recovery.
2. Teach graded exposure and pacing to avoid "boom-bust" flare cycles.
3. Integrate faith-based and modesty-sensitive guidance for physical activity.
4. Use simple home-based examples (walking, prayer movements, chores) to encourage confidence and function.

Core Clinical Concept

Persistent pain leads many patients to stop moving out of fear, which weakens the body, increases sensitivity, and reinforces the fear-pain loop. Movement, when introduced gradually and confidently, retrains the nervous system to perceive safety again.

This approach, known as graded exposure, helps patients re-engage with meaningful activities rather than "exercises for pain."

"Every safe movement is a message of safety to the brain."

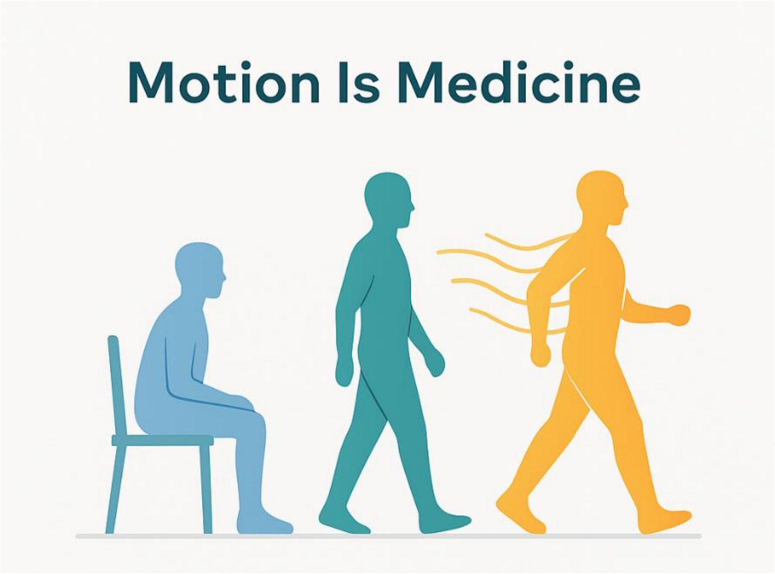

Clinician Tip: Trace the energy lines with your hand: “This is how motion restores calm and flow.”

Teaching Metaphor: The Rusting Door Hinge

“A door that hasn’t moved in months squeaks when it’s opened, not because it’s broken, but because it needs to move again.” This metaphor resonates strongly with Pakistani patients who value practical, domestic examples.
It reinforces that stiffness and pain often come from disuse, not damage.

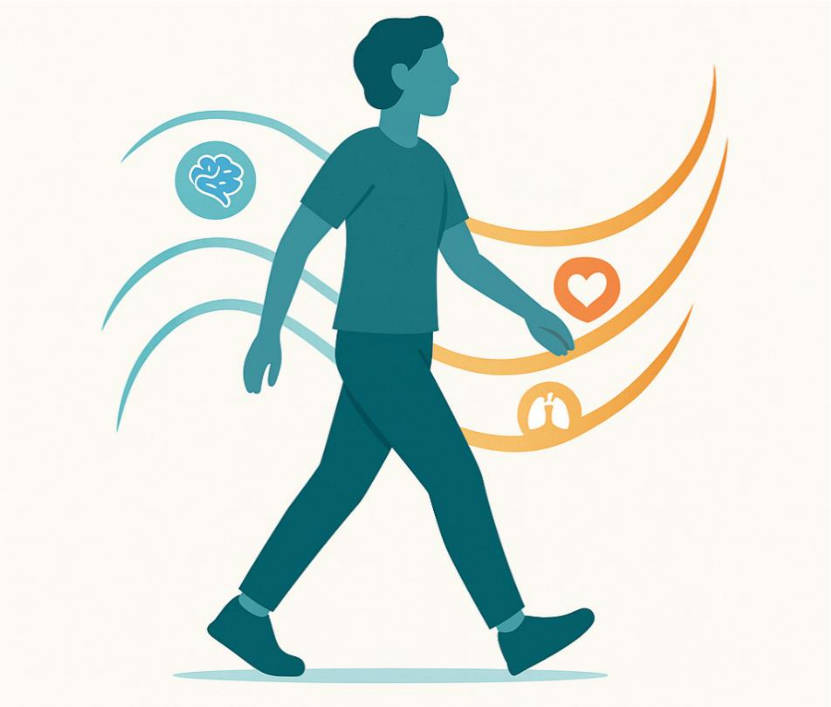

Small movements build big recovery.

Mini Clinical Activity (5–7 minutes)

Objective: Help the patient experience success with movement in-session.

Instructions:

1. Identify one movement the patient fears (e.g., bending, walking, praying).
2. Demonstrate the smallest, safest version (e.g., partial bow, one-minute walk).
3. Ask: “How much pain did that cause?” “اس سے کتنی تکلیف ہوئی؟”
4. Reflect together: “You moved safely, this tells your brain it can trust the body again” “آپ نے یہ حرکت محفوظ طریقے سے کی، یہ آپ کے دماغ کو بتاتا ہے کہ آپ دوبارہ جسم پر بھروسہ کر سکتے ہیں۔”.

5. Set a micro-goal: repeat this 2–3 times a day until confident.

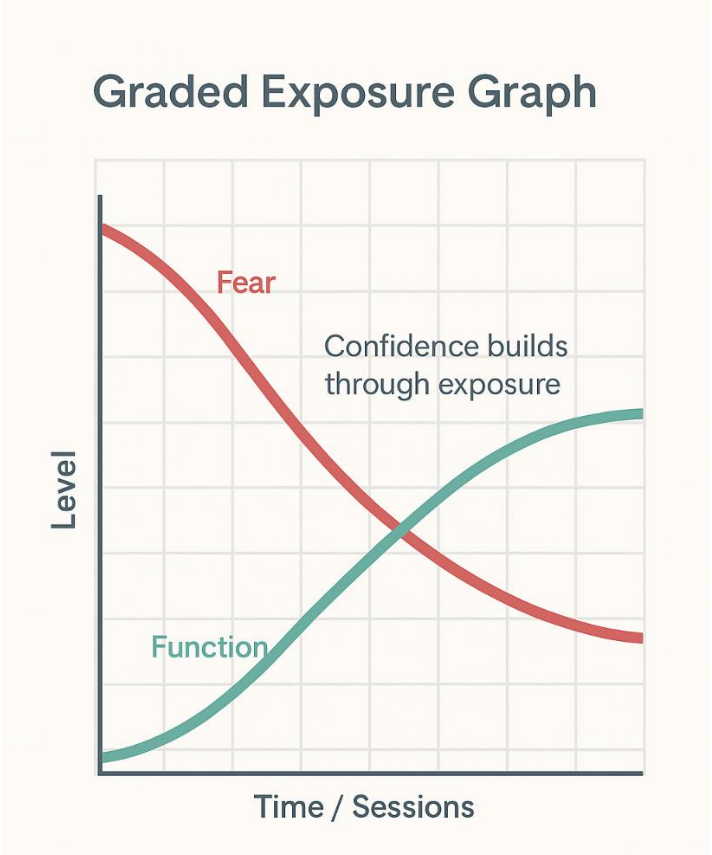

Confidence grows through repeated safe exposure.

<u>Explaining Pacing (Avoiding the Boom–Bust Cycle)</u>

"Boom-bust" pattern: Doing too much on a "good day" → flare-up → days of rest → fear and frustration. Explain pacing as the middle path, consistent, manageable movement that builds endurance and confidence.

Teach with a simple chart:

**Day**	**Time Walking**	**Pain (0–10)**	**Comment**
Mon	5 mins	4	Felt safe
Tue	6 mins	4	Good
Wed	7 mins	5	Slight ache, normal
Thu	7 mins	4	Better tolerance
Fri	8 mins	4	More confident

"We don't chase pain-free days, we build steady, safe days."

"ہم درد سے پاک دنوں کی تلاش نہیں کرتے، ہم مستحکم، محفوظ دن بناتے ہیں۔"

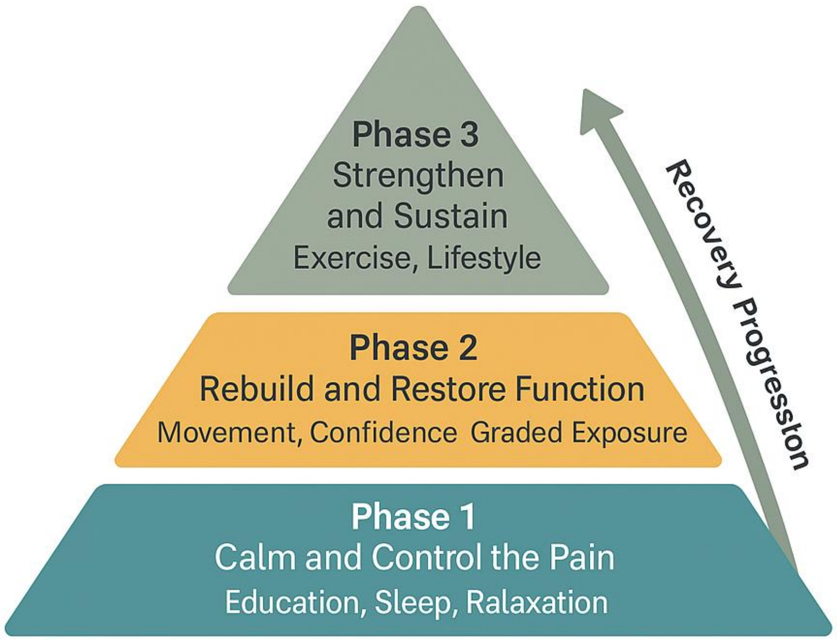

Progress from safety to strength.

Integrating Faith and Culture

Faith and daily routines can serve as built-in rehabilitation opportunities.

- Prayer (Namaz): Movements in prayer such as standing, bowing, sitting are natural mobility exercises. Encourage patients to approach them mindfully, not fearfully.

  "Each prayer is a chance to teach your system calm."

  "ہر نماز آپ کے نظام کو پرسکون سکھانے کا موقع ہے۔"

- Gender and modesty considerations: For female patients, suggest private indoor walking or simple stretches. For male patients, walking to mosque or the market is practical, culturally valued movement.

- Possible faith framing:

  "Allah has designed the body to heal through use, care for it, don't fear it."

  "اللہ تعالیٰ نے جسم کو اس لیے بنایا ہے کہ اس کے استعمال سے ٹھیک ہو، اس کی دیکھ بھال کرو، اس سے ڈرو نہیں۔"

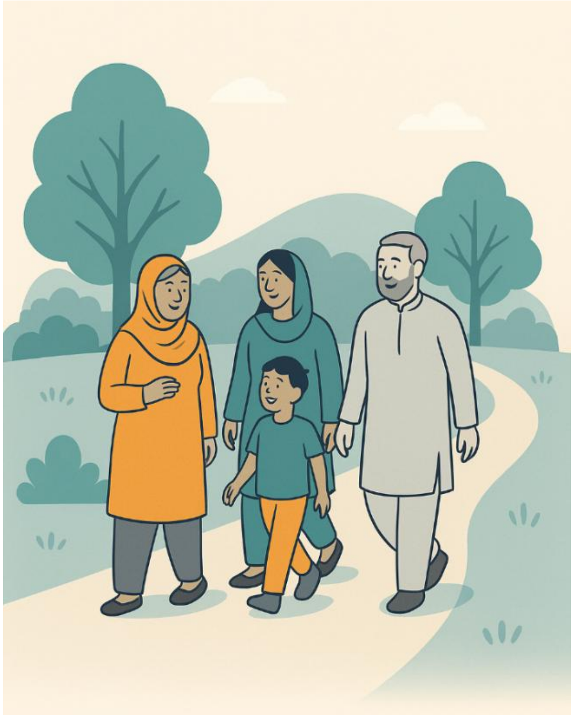

Healing grows through connection and daily movement.

Key Phrases to Use

**Helpful Language**	**Purpose**
“Movement is medicine.” "حرکت دوا ہے۔"	Reinforces active participation.
“Pain with movement doesn’t always mean harm.”<br>"حرکت کے ساتھ درد کا مطلب ہمیشہ نقصان نہیں ہوتا ہے۔"	Builds safety.
“The body learns safety through repetition.”<br>"جسم دہرانے سے حفاظت سیکھتا ہے۔"	Explains neuroplasticity simply.
“Let’s find your safe starting point.”<br>"جسم دہرانے سے حفاظت سیکھتا ہے۔"	Empowers self-efficacy.

Phrases to Avoid

**Unhelpful Language**	**Why It’s Harmful**
“Push through the pain.”<br>“تکلیف کے باوجود سرگرمیاں جاری رکھیں”	Encourages fear or overexertion.
“No pain, no gain.”<br>"اگر درد نہیں، تو فائدہ نہیں."	Reinforces toughness, not safety.
“Rest until it’s gone.”<br>"اس کے ختم ہونے تک آرام کرو۔"	Promotes deconditioning and fear.
“It’s just stiffness.”<br>"یہ صرف پٹھوں کی سختی ہے۔"	Minimises patient concern; breaks trust.

Cultural Communication Tips

1. Frame movement as “normal life,” not “exercise.”
   E.g., walking to market, watering plants, cooking, or sweeping.
2. Encourage family involvement. Invite a spouse, sibling, or child to walk with the patient for reassurance.
3. Respect elders and authority: Use respectful terms like “ چلیے، ہم آپ کے ساتھ مل کر شروع کرتے ہیں۔” (Let’s start together).
4. Acknowledge weather and safety: Suggest indoor walking or gentle household movement during extreme heat.

Clinician Demonstration

Demonstrate 2–3 gentle mobility movements that can be performed in any clothing:

- Shoulder rolls
- Trunk rotation in standing
- Sit-to-stand from a chair
  Use phrases like: “This is not an exercise test; it’s a signal of safety” “ یہ کوئی امتحان نہیں ہے، یہ حفاظت کا اشارہ ہے۔”

Sample Clinician Script

“Many people think movement will make their pain worse. But actually, the body heals through movement. Like a door that rusts when left closed, your body needs gentle use to feel safe again. We’ll start small, a few steps, a few bends, and each one will teach your brain that you are strong.”
“ بہت سے لوگ سوچتے ہیں کہ حرکت ان کے درد کو مزید خراب کر دے گی۔ لیکن درحقیقت، جسم حرکت کے ذریعے ٹھیک ہو جاتا ہے۔ ایک دروازے کی طرح جو بند ہونے پر زنگ لگ جاتا ہے، آپ کے جسم کو دوبارہ محفوظ محسوس کرنے کے لیے نرم استعمال کی ضرورت ہے۔ ہم چھوٹے، چند قدم، چند موڑ شروع کریں گے، اور ہر ایک آپ کے دماغ کو سکھائے گا کہ آپ مضبوط ہیں”

Session Summary for Clinicians

Concept	Clinician Takeaway
Movement retrains sensitivity.	Teach pacing, not rest.
Fear of pain is the real barrier.	Build safety first, then load.
Family support multiplies adherence.	Involve caregivers.
Faith reinforces motivation.	Link movement to gratitude.

End-of-Session Reflection

"Did my patient leave more confident to move today?"
"Did I link movement to meaning: faith, family, or purpose?"

Key Teaching Summary for Cluster 3

Learning Goal	Patient Message	Clinician Action
Explain that movement is safe	"Every step teaches safety."	Use the Rusting Door or Energy Flow metaphor.
Teach pacing	"Start small, stay steady."	Introduce Graded Exposure Graph.
Integrate daily function	"Movement is Amanat (trust)."	Connect exercise to gratitude.

Section 5 – Cluster 4: Healthy Habits & Support

Theme for Patients: "Small daily choices calm the alarm."

Theme for Clinicians: "Healing is a team effort, lifestyle is the long-term medicine."

Clinical Learning Objectives

By the end of this section, clinicians will be able to:

1. Explain how sleep, stress, and nutrition affect pain and healing.
2. Engage families and communities as active participants in recovery.
3. Teach patients how positive language and connection reduce pain sensitivity.
4. Use faith-based, culturally respectful language to promote healthy behaviour change.

Core Clinical Concept

The nervous system does not exist in isolation. Pain intensity is influenced by biological, psychological, and social factors including lifestyle, relationships, and beliefs.
When a patient improves sleep, manages stress, eats well, and feels supported, the nervous system becomes calmer and less reactive.

"You cannot separate the mind, body, and environment. Healing happens when all three align."

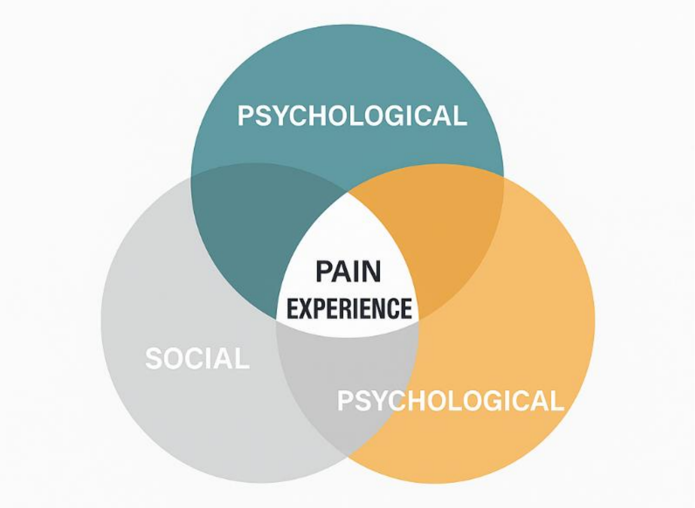

Pain emerges from the interaction of biological, psychological, and social factors.

Clinician Tip: Point to each ring - Biological, Psychological, Social - and say, “All of these talk to your pain system every day” “یہ سب ہر روز آپ کے درد کے نظام سے بات کرتے ہیں۔”.

Teaching Sleep as a Pain Treatment

Clinical script:

“When we don’t sleep well, the brain’s alarm system stays active. Restful sleep resets the system.”

Educate patients on:

- Fixed bedtime and wake time.
- Avoiding heavy meals and screens before sleep.
- Using brief prayer, deep breathing, or light reading to relax the nervous system.

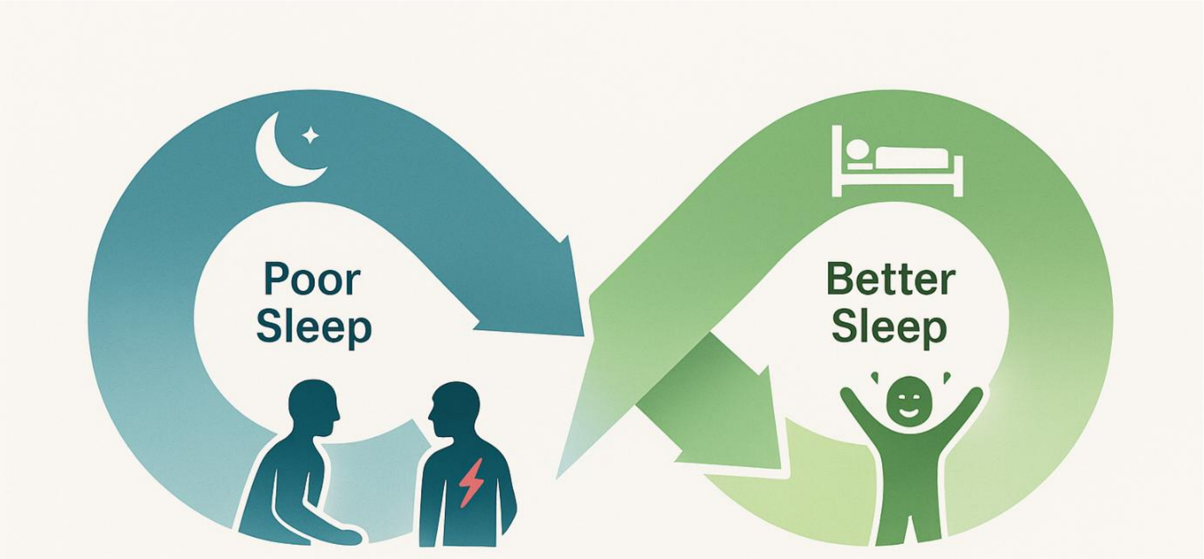

Breaking the cycle restores balance.

“Even improving sleep by one hour a night can make pain less severe — this is real, measurable neurochemistry.”

Explaining Stress to Patients

Stress hormones (adrenaline, cortisol) amplify pain sensitivity. In Pakistan, chronic stress is often tied to family responsibility, financial pressure, and illness anxiety. Clinicians should reframe stress reduction as strength, not weakness.

Say: “ٹینشن کم کرنا کمزوری نہیں، علاج ہے۔” (Reducing stress is not weakness, it is treatment.)

Demonstrate:

- The 3-3-3 breathing technique (3 breaths in, 3 seconds hold, 3 seconds out).
- Gentle shoulder and neck mobility.
- Pair relaxation with prayer or mindful dhikr.

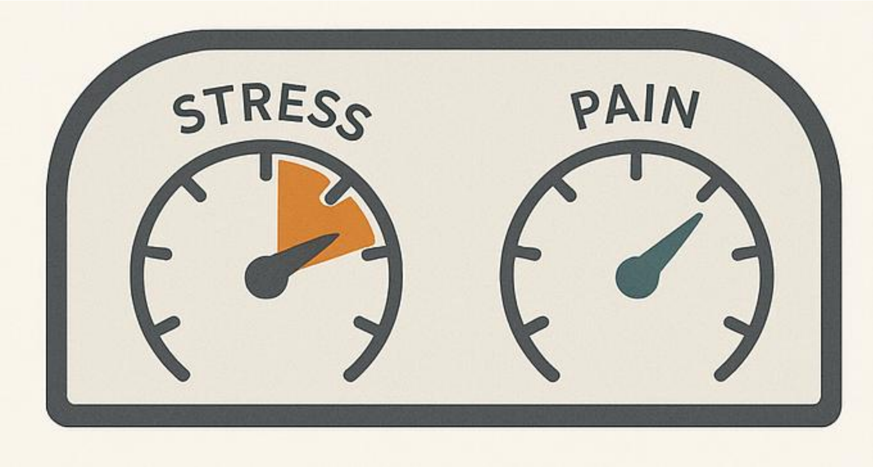

Your pain dashboard can be tuned through daily choices.

Nutrition as Inflammation Control

Explain that diet affects inflammation, energy, and muscle recovery. Encourage patients to choose moderation, not restriction.

Cultural examples:

- Replace fried parathas with lightly cooked versions in olive oil.
- Add daal and vegetables daily.
- Avoid skipping meals when stressed or fasting: plan light, balanced suhoor/iftar meals.

- Encourage hydration: "پانی کا ٹھنڈا ہونا ضروری نہیں ہے، لیکن یہ ضروری ہے۔" *(Water doesn't have to be cold, but it's essential.)*

<u>The Power of Language</u>

Words from clinicians and families shape pain expectations. Negative or fearful language ("Your back is gone" "آپ کی کمر ختم ہوگئی") increases fear and pain. Reassuring language ("Your spine is strong and healing" "آپ کی ریڑھ کی ہڈی مضبوط ہے اور خود ٹھیک ہو رہی ہے۔") increases safety and confidence.

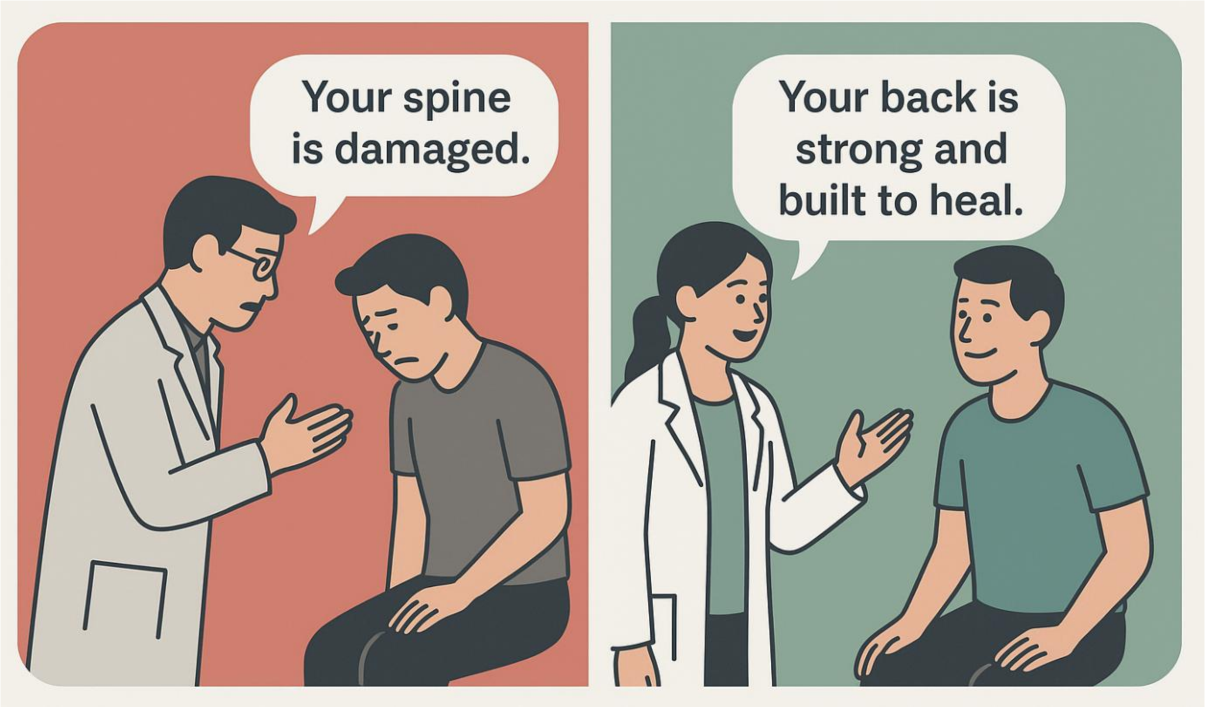

Language can heal or harm.

Encourage family members to speak with confidence and calm:

"آپ بہتر ہو رہے ہیں" (You are improving.) instead of "یہ درد کبھی نہیں جائے گا" (This pain will never go away.)

Faith and Recovery

Faith can be a biopsychosocial healing tool. Clinicians may safely integrate faith-based framing to motivate behavioural change:

- Prayer as mindfulness: Encourage mindful movement and breath focus during prayer as a calming routine.

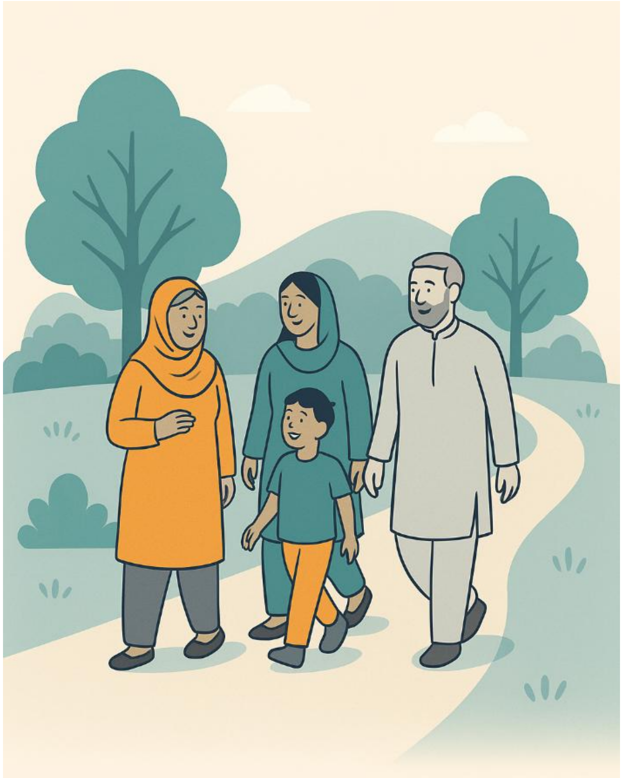

Healing grows through connection.

Engaging Family and Community

In Pakistan, recovery is collective, not individual. Family members often decide treatment plans, control rest, and influence emotion. Instead of challenging this, train them to be allies.

Clinician strategies:

1. Invite one family member into the education session.
2. Ask them: “What can you say or do that makes them feel safe to move?”
3. Praise supportive behaviours e.g., walking together, speaking positively.
4. Encourage small group activities: evening walks, gentle household chores shared together.

Key Phrases to Use

Helpful Language	Purpose
“Healing is a team effort.”<br>"شفا یابی ایک ٹیم کی کوشش ہے۔"	Normalises family involvement.
“Small daily choices train the system.”<br>"روزانہ رویے نظام کو تربیت دے سکتے ہیں۔"	Emphasises behavioural change.
“Faith and knowledge work together.”<br>"ایمان اور علم ایک ساتھ کام کرتے ہیں۔"	Integrates spirituality.
“You are not broken; you are rebuilding.”<br>"آپ ٹوٹے نہیں ہیں، آپ دوبارہ تعمیر کر رہے ہیں."	Reframes recovery positively.

Phrases to Avoid

Unhelpful Language	Why It’s Harmful
“Lifestyle doesn’t matter.”<br>"طرز زندگی سے کوئی فرق نہیں پڑتا۔"	Devalues patient agency.
“This is all stress.”<br>"یہ سب تناؤ ہے۔"	Oversimplifies, invalidates pain.
“Stop thinking too much.”<br>"زیادہ سوچنا بند کرو۔"	Blames patient.
“You just need to rest.”<br>"آپ کو صرف آرام کرنے کی ضرورت ہے۔"	Reinforces fear-avoidance.

Mini Clinical Activity: “The Daily Routine Check” (5 minutes)

Objective: Identify one small, achievable habit to support recovery.

Instructions:

1. Ask: “What does your morning look like?”
2. Identify one target: e.g., walking after prayer, reducing phone time before sleep.
3. Say: “This small change will calm your system like a reset button.”
4. Record it and review next session.

Session Summary for Clinicians

**Concept**	**Clinician Takeaway**
Lifestyle changes calm the nervous system.	Teach sleep, food, and stress as medicine.
Language and support shape pain recovery.	Train families to “speak safety.”
Faith reinforcing motivation and calm.	Integrate belief-based encouragement.
Connection and community sustain healing.	Use group or family-based follow-ups.

Sample Clinician Script

“Pain is not only in the back, it lives in the whole system. When sleep improves, stress reduces, and the family supports you; your alarm becomes calm. This is not just lifestyle advice; it’s part of your treatment. Your home, words, and habits can become your medicine.”

“ درد صرف کمر میں نہیں ہوتا، یہ پورے نظام میں رہتا ہے۔ جب نیند بہتر ہوتی ہے، تناؤ کم ہوتا ہے، اور خاندان آپ کی مدد کرتا ہے۔ آپ کا الارم پرسکون ہو جاتا ہے۔ یہ صرف طرز زندگی کا مشورہ نہیں ہے۔ یہ آپ کے علاج کا حصہ ہے. آپ کا گھر، الفاظ اور عادتیں آپ کی دوا بن سکتی ہیں۔ ”

End-of-Session Reflection

“Did I address the patient’s life, not just their body?”
“Did my language create a plan, or just advice?”

Key Teaching Summary for Cluster 4

**Learning Goal**	**Patient Message**	**Clinician Action**
Teach that lifestyle influences pain	“Daily choices calm the alarm.”	Use Sleep-Pain and Dashboard Dials visuals.
Emphasise supportive communication	“Words can heal or harm.”	Engage family in language change.
Integrate faith and social strength	“Create calm through faith.”	Use Cultural Strengths visuals and phrases.

Section 6 – Cluster 5: Recovery and Hope

Theme for Patients: "You can retrain your system."

Clinical Learning Objectives

By the end of this section, clinicians will be able to:

1. Explain neuroplasticity (the brain's capacity to change) in simple, visual terms.
2. Teach hope and self-efficacy as *biological treatments*, not just emotions.
3. Coach patients in relapse prevention through pacing, reflection, and social support.
4. Integrate faith-based reassurance while maintaining scientific accuracy.

Core Clinical Concept

Recovery is not a single event. It is a process of retraining the nervous system through repetition, learning, and confidence. Neuroplasticity means that the brain's pain networks can "quiet down" and rebuild stronger, calmer connections when patients move, learn, and feel safe.

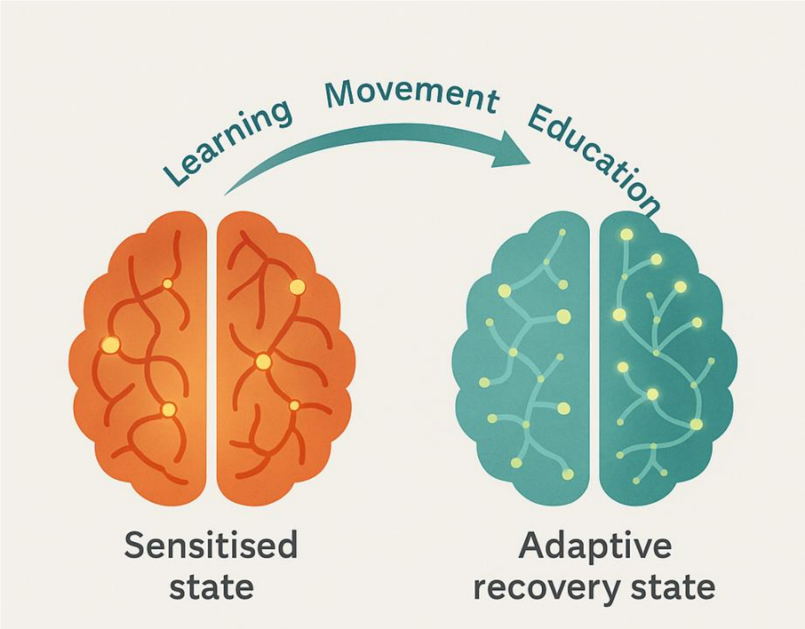

Clinician Tip: Trace from the red side (sensitised brain) to the green side (adaptive brain).

Teaching the Recovery Pyramid

Explain that recovery occurs in three overlapping phases, each building on the other.

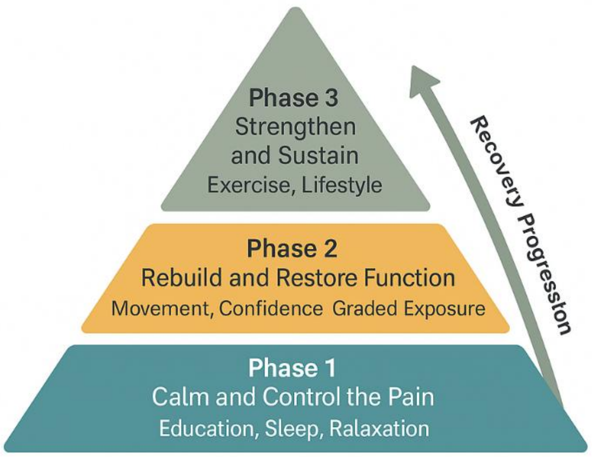

Progress from safety to strength.

Phase	Focus	Patient Message	Clinician Strategy
**1. Calm & Control**	Reassurance, breathing, sleep	"You are safe."	Teach education + calm techniques.
**2. Rebuild & Restore**	Gentle movement, pacing	"Your body is learning."	Guide graded exposure, track confidence.
**3. Strengthen & Sustain**	Exercise, connection, lifestyle	"You are capable."	Reinforce self-management, relapse prevention.

"Each phase is not separate, they blend like steps in prayer: calm, movement, strength."

Active vs Passive Recovery

Clinicians should help patients shift from dependency to participation.

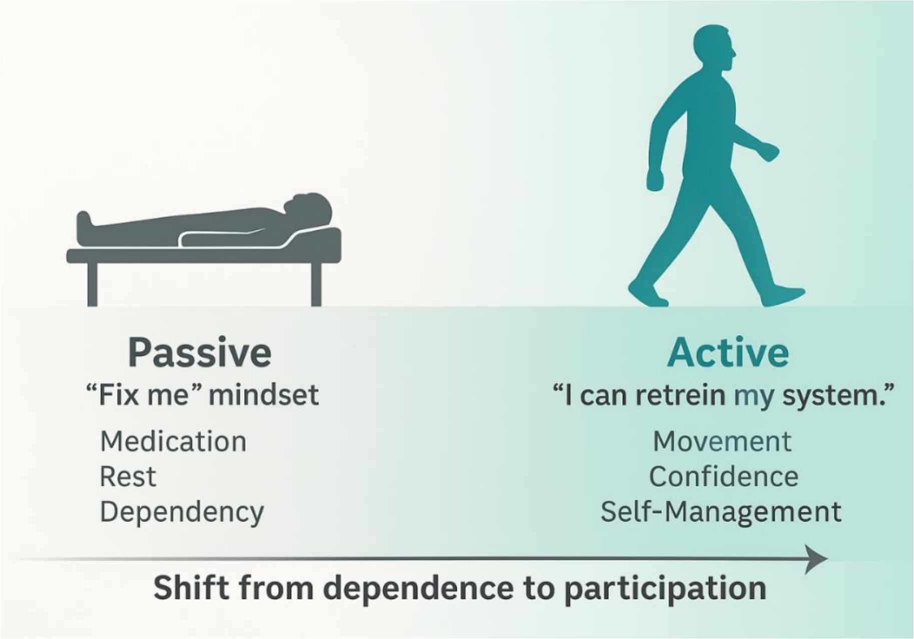

Shift from dependence to participation.

Explain:

“Treatments like medication or massage can help, but they can’t replace your own participation. The brain changes when *you* move, learn, and act. That’s how neuroplasticity works.”

“ ادویات یا مساج جیسے علاج مدد کر سکتے ہیں، لیکن وہ علاج میں آپ کی اپنی شرکت کی جگہ نہیں لے سکتے۔ جب آپ حرکت کرتے، سیکھتے اور عمل کرتے ہیں تو دماغ بدل جاتا ہے۔ اس طرح نیوروپلاسٹیٹی کام کرتی ہے۔”

**Passive Mindset**	**Active Mindset**
“Doctor will fix me.”<br>"ڈاکٹر مجھے ٹھیک کر دیں گے۔"	“I can retrain my body.”<br>"میں اپنے جسم کو دوبارہ تربیت دے سکتا ہوں۔"
“I need complete rest.”<br>‘‘مجھے مکمل آرام کی ضرورت ہے۔’’	“I’ll start small and build.”<br>" میں چھوٹے قدموں سے شروع کروں گا اور بہتر ہو جاؤں گا۔"
“I’ll never be the same.”<br>"میں پہلے جیسا نہیں رہوں گا۔"	“I’m learning to adapt.”<br>"میں خود کو ڈھالنا سیکھ رہا ہوں۔"

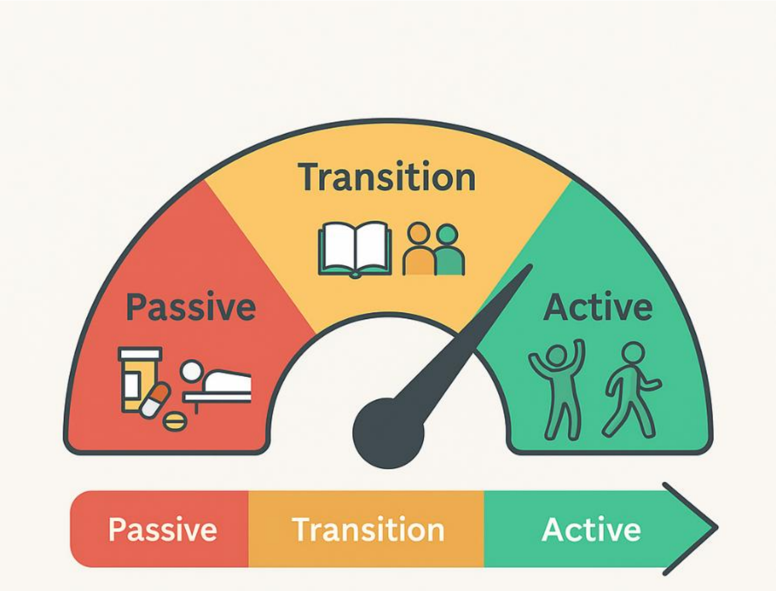

Preventing Relapse

Clinician education message:

“Pain will fluctuate. That’s biology, not failure.” "درد میں اتار چڑھاؤ آئے گا۔ یہ حیاتیات ہے، ناکامی نہیں۔"

Teach patients that flare-ups are opportunities for reinforcement, not relapse.

Flare-up plan:

1. Pause, breathe, remind: “My system is safe.” “میرا سسٹم محفوظ ہے۔”
2. Return to calm routines (sleep, pacing, stretching).
3. Reconnect with supportive people.
4. Avoid fear-driven rest. Instead, modify activity and continue gently.

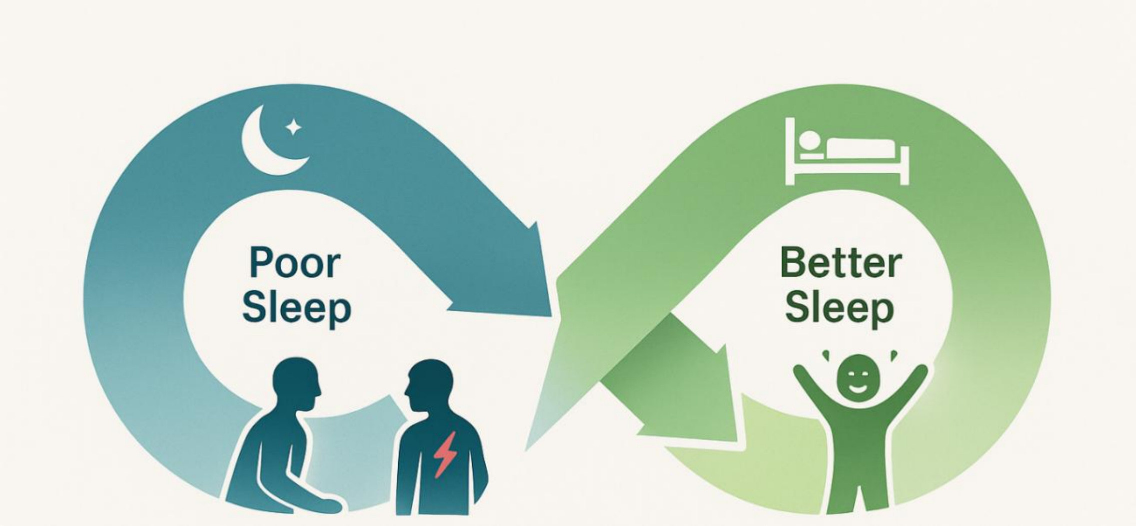

Key Phrases to Use

Helpful Language	Purpose
“Your brain can change.” "آپ کا دماغ بدل سکتا ہے۔"	Simplifies neuroplasticity.
“Flare-ups are normal; they don’t mean failure.”<br>"درد کے بھڑک اٹھنا معمول کی بات ہے؛ ان کا مطلب ناکامی نہیں ہے۔"	Builds resilience.
“You’ve learned calm, now your system remembers it.”<br>"آپ نے سکون سیکھ لیا ہے - اب آپ کا سسٹم اسے یاد رکھتا ہے۔"	Reinforces progress.

Phrases to Avoid

Unhelpful Language	Why It’s Harmful
“You’ll have to live with it.”<br>"آپ کو اس درد کے ساتھ رہنا پڑے گا۔"	Destroys hope.
“It’s all in your mind.”<br>"یہ سب آپ کے ذہن میں ہے۔"	Stigmatises and confuses.
“You’re cured now.”<br>’’اب تم ٹھیک ہو گئے ہو۔‘‘	Creates unrealistic expectation.
“Don’t ever do that again.”<br>"دوبارہ ایسا مت کرنا۔"	Reinforces fear.

Mini Clinical Activity: “Rebuilding the Story” (10 minutes)

Objective: Reframe patient narrative from fear to recovery.

Instructions:

1. Ask: “What have you learned about your pain so far?”
2. Note their beliefs.
3. Write new statements together:
    - From “My back is weak” → to “My back is adapting.”
    - From “I’ll never recover” → to “My body learns every day.”
4. Ask: “Who helps you remember this?” (Engage family/community support.)

Session Summary for Clinicians

Concept	Clinician Takeaway
Movement and belief reshape pain networks.	Active recovery = adaptive brain.
Faith can reinforce motivation.	Align science with purpose.
Relapse prevention is education, not fear.	Teach calm re-engagement.

Sample Clinician Script

“Pain doesn’t mean your body is weak. It means your system is learning. The more you move, rest wisely, and stay hopeful, the calmer your brain becomes. Each prayer, breath, and step teaches safety.”

" درد کا مطلب یہ نہیں ہے کہ آپ کا جسم کمزور ہے۔ اس کا مطلب ہے کہ آپ کا نظام سیکھ رہا ہے۔ آپ جتنا زیادہ حرکت کرتے ہیں، عقلمندی سے آرام کرتے ہیں، اور پر امید رہتے ہیں، آپ کا دماغ اتنا ہی پرسکون ہوتا جاتا ہے۔ ہر دعا، سانس اور قدم حفاظت کا درس دیتے ہیں۔"

End-of-Session Reflection

“Did I leave my patient with a plan or just reassurance?”
“Did my language create fear or faith in their ability to recover?”

Key Teaching Summary for Cluster 5

Learning Goal	Patient Message	Clinician Action
Prevent relapse	“Flare-ups are learning moments.”	Create written flare-up plans.
Align faith and science	“Patience + effort = recovery.”	Use faith-framed encouragement.

Closing Message for Clinicians

“Pain is not a signal to stop. It is an invitation to understand.”
“درد رکنے کا اشارہ نہیں ہے، یہ سمجھنے کی دعوت ہے۔ "

References

1. Louw, A.; Zimney, K.; O'Hotto, C.; Hilton, S. The clinical application of teaching people about pain. *Physiotherapy Theory and Practice* **2016**, *32* (5), 385–395. https://doi.org/10.1080/09593985.2016.1194652.
2. O'Sullivan, P. B.; Caneiro, J.; O'Sullivan, K.; Lin, I.; Bunzli, S.; Wernli, K.; O'Keeffe, M. Back to basics: 10 facts every person should know about back pain. *British Journal of Sports Medicine* **2019**, *54* (12), 698–699. https://doi.org/10.1136/bjsports-2019-101611.
3. Traeger, A. C.; Lee, H.; Hübscher, M.; Skinner, I. W.; Moseley, G. L.; Nicholas, M. K.; Henschke, N.; Refshauge, K. M.; Blyth, F. M.; Main, C. J.; Hush, J. M.; Lo, S.; McAuley, J. H. Effect of Intensive Patient Education vs Placebo Patient Education on Outcomes in Patients With Acute Low Back Pain. *JAMA Neurology* **2018**, *76* (2), 161. https://doi.org/10.1001/jamaneurol.2018.3376.
4. Moseley, G. L.; Butler, D. S. *Explain pain supercharged*; 2017.
5. Butler, D. S.; Moseley, G. L. *Explain pain 2nd EdN.*; Noigroup Publications, 2013.
6. Vlaeyen, J. W. S.; Linton, S. J. Fear-avoidance and its consequences in chronic musculoskeletal pain: a state of the art. *Pain* **2000**, *85* (3), 317–332. https://doi.org/10.1016/s0304-3959(99)00242-0.
7. Ramond-Roquin, A.; Bouton, C.; Bègue, C.; Petit, A.; Roquelaure, Y.; Huez, J.-F. Psychosocial Risk Factors, Interventions, and Comorbidity in Patients with Non-Specific Low Back Pain in Primary Care: Need for Comprehensive and Patient-Centered Care. *Frontiers in Medicine* **2015**, *2*, 73. https://doi.org/10.3389/fmed.2015.00073.
8. Kamper, S. J.; Apeldoorn, A. T.; Chiarotto, A.; Smeets, R. J. E. M.; Ostelo, R. W.; Guzman, J.; Van Tulder, M. W. Multidisciplinary biopsychosocial rehabilitation for chronic low back pain. *Cochrane Database of Systematic Reviews* **2014**, *2014* (9), CD000963. https://doi.org/10.1002/14651858.cd000963.pub3.
9. Crombez, G.; Eccleston, C.; Van Damme, S.; Vlaeyen, J. W. S.; Karoly, P. Fear-Avoidance model of chronic pain. *Clinical Journal of Pain* **2012**, *28* (6), 475–483. https://doi.org/10.1097/ajp.0b013e3182385392.
10. Apkarian, A. V. The Brain in Chronic Pain: clinical implications. *Pain Management* **2011**, *1* (6), 577–586. https://doi.org/10.2217/pmt.11.53.
11. Su, Q.; Song, Y.; Zhao, R.; Liang, M. A review on the ongoing quest for a pain signature in the human brain. *Brain Science Advances* **2019**, *5* (4), 274–287. https://doi.org/10.26599/bsa.2019.9050024.
12. McGill, S. M. *Low Back Disorders, 3E*; Human Kinetics, 2015.
13. Geneen, L. J.; Moore, R. A.; Clarke, C.; Martin, D.; Colvin, L. A.; Smith, B. H. Physical activity and exercise for chronic pain in adults: an overview of Cochrane Reviews. *Cochrane Database of Systematic Reviews* **2017**, *2020* (2), CD011279. https://doi.org/10.1002/14651858.cd011279.pub3.

14. Hodges, P. W.; Tucker, K. Moving differently in pain: A new theory to explain the adaptation to pain. *Pain* **2010**, *152* (3), S90–S98. https://doi.org/10.1016/j.pain.2010.10.020.
15. López-De-Uralde-Villanueva, I.; Muñoz-García, D.; Gil-Martínez, A.; Pardo-Montero, J.; Muñoz-Plata, R.; Angulo-Díaz-Parreño, S.; Gómez-Martínez, M.; La Touche, R. A Systematic Review and Meta-Analysis on the Effectiveness of Graded activity and Graded Exposure for chronic Nonspecific low back pain. *Pain Medicine* **2015**, *17* (1), 172–188. https://doi.org/10.1111/pme.12882.
16. Shimada, S.; Doorenbos, A. Z.; Goldstein, E.; Wi, D. A Systematic review of Pain Catastrophizing and Chronic Musculoskeletal pain. *Pain Management Nursing* **2025**. https://doi.org/10.1016/j.pmn.2025.07.014.
